# Evaluating the Effectiveness of a Mobile HIV Prevention App to Increase HIV and Sexually Transmitted Infection Testing and Pre-Exposure Prophylaxis Initiation Among Rural Men Who Have Sex With Men in the Southern United States: Protocol for a Randomized Controlled Trial

**DOI:** 10.2196/69540

**Published:** 2025-07-23

**Authors:** Jeb Jones, Georgia Manley, Tiffany R Glynn, Kristin M Wall, Stefan D Baral, E Danny Harris, David Benkeser, Patrick S Sullivan

**Affiliations:** 1 Department of Epidemiology Emory University Atlanta, GA United States; 2 Department of Psychiatry Massachusetts General Hospital Harvard Medical School Boston, MA United States; 3 Department of Emergency Medicine Brigham and Women's Hospital Boston, MA United States; 4 Department of Epidemiology Johns Hopkins School of Public Health Baltimore, MD United States; 5 Engaging Arkansas Communities Little Rock, AR United States; 6 Department of Biostatistics and Bioinformatics Emory University Atlanta, GA United States

**Keywords:** HIV, rural, men who have sex with men, testing, pre-exposure prophylaxis

## Abstract

**Background:**

Gay and bisexual men who have sex with men (MSM) in the rural United States are less likely to test for HIV and sexually transmitted infections (STIs) and use pre-exposure prophylaxis (PrEP) compared to urban MSM. Given the challenges in building brick-and-mortar facilities serving the sexual health needs of MSM in rural communities, there is a need to evaluate digital engagement strategies. Combine is a smartphone app designed to provide HIV prevention information and resources to MSM that may be particularly well suited to support rural MSM. HealthMindr, an app from which Combine is adapted, has previously been shown to increase HIV testing and PrEP uptake among urban MSM. Because rural MSM face additional barriers to accessing HIV prevention services, a motivational interview (MI) focused on HIV prevention strategies might enhance the effectiveness of Combine to increase uptake of HIV testing, STI testing, and PrEP.

**Objective:**

This study aims to determine the effectiveness of the Combine app to increase HIV testing, STI testing, and PrEP initiation among rural cisgender MSM. We will also assess the effectiveness of 2 adjunctive interventions: the availability of free HIV and STI self-test kits and the offering of an MI.

**Methods:**

In this type 2 hybrid effectiveness-implementation randomized controlled trial, we will assess the effectiveness of Combine to increase HIV testing, STI testing, and PrEP initiation among rural MSM across the Southern United States. A total of 464 men will be recruited and randomized to 1 of the 4 arms. Participants in all 4 arms will have access to most app features (eg, health resources, quizzes, health care provider locators, and ordering free condoms and lubricants). Using a 2×2 factorial design, half (232/464, 50%) of the participants will be randomized to receive access to free at-home HIV and STI self-test kits and half (232/464, 50%) will be randomized to receive an MI. Participants will complete surveys every 6 months to allow for assessment of self-reported outcomes: app use, HIV testing, STI testing, and PrEP initiation over the 24-month follow-up period. Self-reported PrEP uptake will be verified by dried blood spot testing or uploading a photograph of a PrEP prescription.

**Results:**

Participant recruitment began in March 2024. As of July 2025, 395 participants have been enrolled and randomized. Recruitment is expected to be completed by December 2025.

**Conclusions:**

This trial will determine whether the Combine app increases HIV testing, STI testing, and PrEP uptake among rural MSM in the Southern United States. It will also provide critical information about the most effective strategies for implementing digital health interventions for rural MSM.

**Trial Registration:**

ClinicalTrials.gov NCT06205368; https://clinicaltrials.gov/study/NCT06205368

**International Registered Report Identifier (IRRID):**

DERR1-10.2196/69540

## Introduction

### Background

Gay, bisexual, and other men who have sex with men (MSM) are disproportionately affected by HIV and other sexually transmitted infections (STIs), including in the rural Southern United States. The Southern United States represents just 37% of the US population [1], but in 2022, 47% of all new HIV infections attributable to male-to-male sexual contact occurred there [2]. The Southern United States is also home to 6 of 7 states identified as priority jurisdictions for the Ending the HIV Epidemic campaign due to the number of new diagnoses in these states occurring in rural areas [3]. The seventh state, Missouri, is geographically contiguous with the Southern United States. Many of the rural counties in Alabama, Georgia, Mississippi, and South Carolina are in the top decile of HIV prevalence in the United States [4].

The HIV epidemic in rural areas resembles the epidemic in urban areas in that the unmet HIV prevention and treatment needs among MSM account for most new diagnoses. In 2018, 77% of new HIV diagnoses in rural areas were among men, and among those, 77% were attributable to male-to-male sexual contact, similar to the proportions attributable to male-to-male sexual contact in urban (83%) and suburban (79%) areas [5]. Moreover, Black people in the Southern United States bear a disproportionate burden of new HIV diagnoses, accounting for 53% of all new diagnoses in 2017; 60% of these diagnoses were among Black MSM [6]. New HIV diagnoses have been increasing steadily among Hispanic MSM and decreasing among non-Hispanic White MSM [6]. Overall, in the United States, 80% of rural residents are non-Hispanic White [7], whereas the overall US population is 60% non-Hispanic White [1]. However, southern states have much larger populations of rural residents of color [8]. Of the 17 states that comprise the Southern United States, 13 have >20% rural populations of color; 3 states (South Carolina, Mississippi, and Texas) have rural populations comprising >40% people of color [8]. Rural MSM, particularly rural MSM of color, represent a key group to prioritize in order to improve HIV and STI health equity across multiple dimensions.

Uptake of HIV testing, STI testing, and pre-exposure prophylaxis (PrEP) among MSM in rural areas is low. MSM in rural areas face a number of challenges in accessing these services, including limited access to culturally competent care, increased experiences of stigma, and lower self-perceived risk of HIV and STI infections compared to MSM in suburban and urban areas [9,10]. HIV testing is the foundation of the National HIV/AIDS Strategy [11] because it serves as the entry point to prevention programs, such as HIV PrEP, among those who test negative and HIV treatment initiation among those who test positive. The Centers for Disease Control and Prevention recommends that sexually active MSM be tested for HIV, *Neisseria gonorrhoeae*, *Chlamydia trachomatis*, and syphilis at least annually [12,13]; more frequent STI screening is recommended for MSM at elevated risk [13]. However, only 40% of rural MSM have been tested for HIV in the past 12 months [14]. STI testing uptake is even lower, particularly for rectal and pharyngeal STIs; <25% of rural MSM have had an STI test in the past 12 months, and <10% have been tested at any extragenital sites. Sexual behavior is remarkably consistent across levels of rurality; in a recent study, approximately 71% of MSM had indications for PrEP, irrespective of rurality [14]. Despite similar levels of awareness and willingness to use PrEP, PrEP use is drastically lower in rural compared to nonrural MSM [15].

Mobile health (mHealth) interventions are effective tools for increasing HIV and STI testing uptake among urban MSM [16-18], and using technologies to support HIV prevention is acceptable to rural MSM [19,20]. Evidence is needed to demonstrate that the acceptability of mHealth interventions among rural MSM translates to intervention effectiveness. Rural MSM have been found to have a strong interest in accessing health care remotely, including at-home HIV and STI testing and tele-PrEP, to assuage concerns about confidentiality and stigma [21,22]. Smartphones are widely accessible to MSM who are most at risk. Overall, among Americans, 96% of people aged 18 to 29 years, 80% of African American people, and 80% of rural residents own smartphones [23]. The internet has also been proven effective in reaching MSM in rural areas to promote HIV prevention [24].

In this type 2 hybrid effectiveness-implementation randomized controlled trial (RCT), we will test an adaptation of HealthMindr, a mobile HIV prevention platform for MSM [25]. HealthMindr is grounded in social cognitive theory [26]. Its development has been described elsewhere [25,27,28], and multiple adaptations of the HealthMindr platform have been tested among different priority populations [17,29,30]. M-Cubed, an adaptation of HealthMindr that provides pop-up HIV prevention messaging, demonstrated efficacy to increase HIV testing and PrEP uptake among urban MSM [17].

There is a need to understand which *components* of app-based HIV prevention are necessary to promote prevention to inform future implementation. A standard feature of most app-based HIV prevention interventions is access to free HIV and STI self-test kits [30-32]. These may be particularly attractive to MSM in rural areas for whom options for HIV testing and extragenital STI screening are limited. Because mail-out test kits have been universally available in previous studies, it is not clear if they are a necessary component of these interventions, as an app-only control condition without self-test kits is not available for comparison. Future implementation will be logistically more complicated if test kits need to be provided to ensure the effectiveness of the app, and some public health officials are hesitant to implement at-home testing programs. We need to know whether self-test kits are necessary to promote testing uptake and whether the kits are necessary for the success of other aspects of the interventions (eg, PrEP uptake). For example, HealthMindr has been found to increase PrEP uptake [17], but it is not clear if this effect would persist in the absence of free self-testing.

Brief behavioral motivational interviewing–based interventions are effective in preventing HIV and STI transmission [33], and motivational interviews (MIs) might be an effective adjunctive intervention to increase engagement with an app-based intervention and improve outcomes. Engagement with app content and maintenance of use is another challenge for technology-delivered HIV prevention interventions [16]. In the pilot study of the HealthMindr app, only 25% of the participants continued to use the app throughout the full 4 months of follow-up [25]. Other studies of app-based interventions have encountered similar drop-offs in use over similar follow-up periods [16]. Sustained use over longer follow-up periods will almost certainly be lower. Although alterations to app design and aesthetics might improve user engagement, adjunctive interventions, such as motivational interviewing, might increase user motivation to use app-based interventions on a sustained basis while also providing an opportunity to increase users’ ability to overcome barriers related to privacy, confidentiality, and stigma. Motivational interviewing is an effective tool for reducing HIV risk behavior [33]; however, additional data are needed to understand the effectiveness of motivational interviewing among rural MSM, particularly in the context of mHealth interventions.

Apps are an effective method to increase HIV and STI prevention service uptake among MSM in urban areas. Rural MSM face additional barriers that might mitigate the effectiveness of app-based interventions, and there are outstanding questions about the most effective implementation strategies for these interventions. To address these knowledge gaps, we adapted an existing evidence-based app, HealthMindr, into a new app, Combine, specifically focused on rural MSM. We conducted online focus group discussions with rural MSM about their health care experiences and obtained feedback on Combine. Participants had a wide range of backgrounds with respect to accessing HIV prevention services, and an overwhelming majority expressed interest in using Combine to access information and services related to HIV prevention and expected that others in their peer group felt the same [19]. On the basis of participant reports of limited formal knowledge about sex and sexual health, we have expanded the frequently asked questions to provide more detailed information about sex, HIV, and STIs, including options for reducing risks, common symptoms of HIV and different STIs, and treatment options, and tips for negotiating prevention options with a sex partner. We also added enhanced service navigation support, including the option to connect with study staff directly through the app via in-app messaging to receive personalized support.

We conducted an online survey of rural MSM and asked participants about the availability and reliability of cellular service where they lived and their willingness to participate in a research study of a mobile HIV prevention app [34]. Cellular coverage and data access were widely available. A total of 80.3% (147/183) of the participants reported reliable coverage, indicating they could obtain a cellular signal whenever they needed one. Only 3.8% (7/183) of the respondents classified their coverage as unreliable. Large proportions of respondents reported conducting data-intensive activities (unrelated to HIV prevention activities) on their phones, including watching videos (159/183, 86.9%), and 89.1% (163/183) of the participants reported never experiencing difficulties downloading apps due to bandwidth issues. Overall, 89% (162/182) of the participants were willing to download a mobile app as part of an HIV prevention research study.

### This Study

Using a 2×2 factorial RCT, we will assess the effectiveness of the Combine app to increase HIV testing, STI testing, and PrEP use among rural MSM and evaluate the effect of 2 specific implementation approaches on testing uptake and app engagement: the availability of HIV and STI self-test kits and an MI intervention to enhance the existing app features. We hypothesize that HIV testing, STI testing, and PrEP uptake will be higher in conditions that include HIV and STI self-test kits or the MI and that uptake of all 3 will be highest in the condition that includes both test kits and the MI.

## Methods

### Overview

We will conduct a type 2 hybrid implementation-effectiveness, 2×2 factorial RCT to evaluate the effectiveness of the Combine app to increase HIV testing, STI testing, and PrEP initiation among rural cisgender MSM and to assess the effectiveness of 2 adjunctive interventions: an MI and the availability of HIV and STI self-test kits. A detailed description of the components included in the Combine app is provided subsequently. After randomization, participants in intervention arms have access to additional features. Those in intervention arms 1 and 3 have the option to participate in an MI (described subsequently) with a trained study staff member. Those who are in intervention arms 2 and 3 have access to order free at-home STI and HIV testing kits. Participants in the control arm (ie, arm 0) will have access to the app but not to the MI or the HIV and STI test kits. We chose to provide participants in this arm with the app because this app platform has been previously demonstrated to increase HIV prevention service uptake among nonrural MSM [17]; therefore, we did not deem it ethical to withhold the app from participants in the control arm.

**Figure 1:**
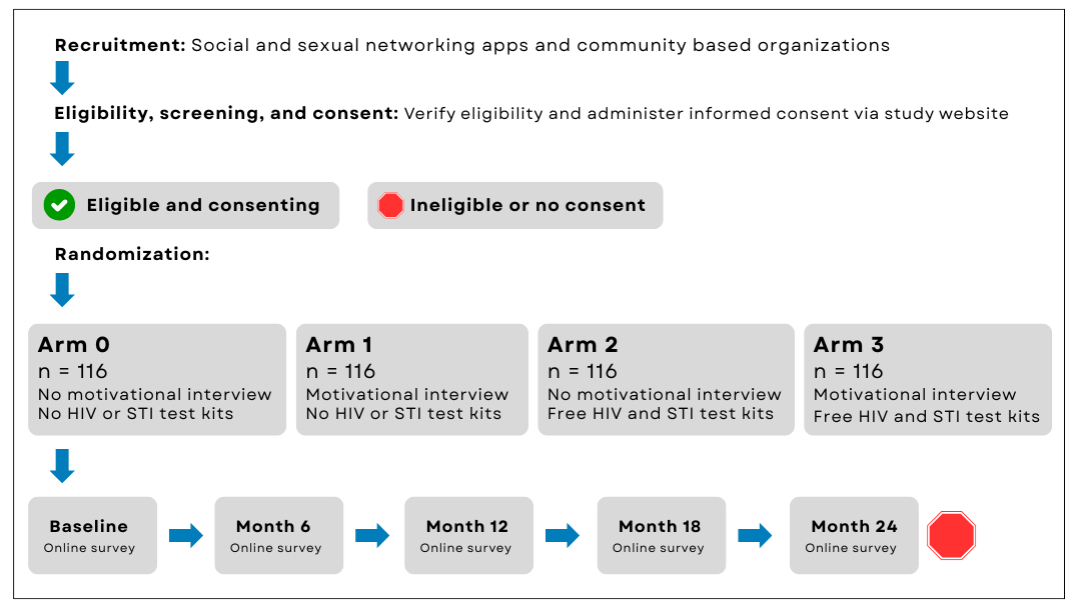
Flow of recruitment, enrollment, and follow-up in a randomized controlled trial of a mobile health intervention among rural, gay, bisexual, and other men who have sex with men in the Southern United States. STI: sexually transmitted infection.

Participants will be followed for 24 months as shown in Figure 1. Primary and secondary outcome measurements will be conducted via online self-administered surveys every 6 months using a Health Insurance Portability and Accountability Act (HIPAA)–compliant survey platform. Surveys will take approximately 30 to 45 minutes to complete. To limit survey duration, some nonlabile survey elements (eg, race, age) will not be assessed at every time point. There will be minimal differences in follow-up surveys depending on intervention assignment; otherwise, surveys will be identical for all those enrolled. All surveys will be optimized for mobile devices.

### Study App

#### Overview

The Combine app is embedded within the Study Management and Retention Toolkit (SMaRT) web participant application. SMaRT has been used to promote participant retention and management in multiple previous studies [29,30,35,36]. The SMaRT web application automates regularly scheduled participant contacts (eg, prompts to complete follow-up surveys); allows staff and study participants to exchange secure messages, including return of HIV and STI test results upon request through a HIPAA-compliant file exchange; and allows participants to see their personalized study timeline. The SMaRT app also contains all the Combine intervention content.

**Figure 2:**
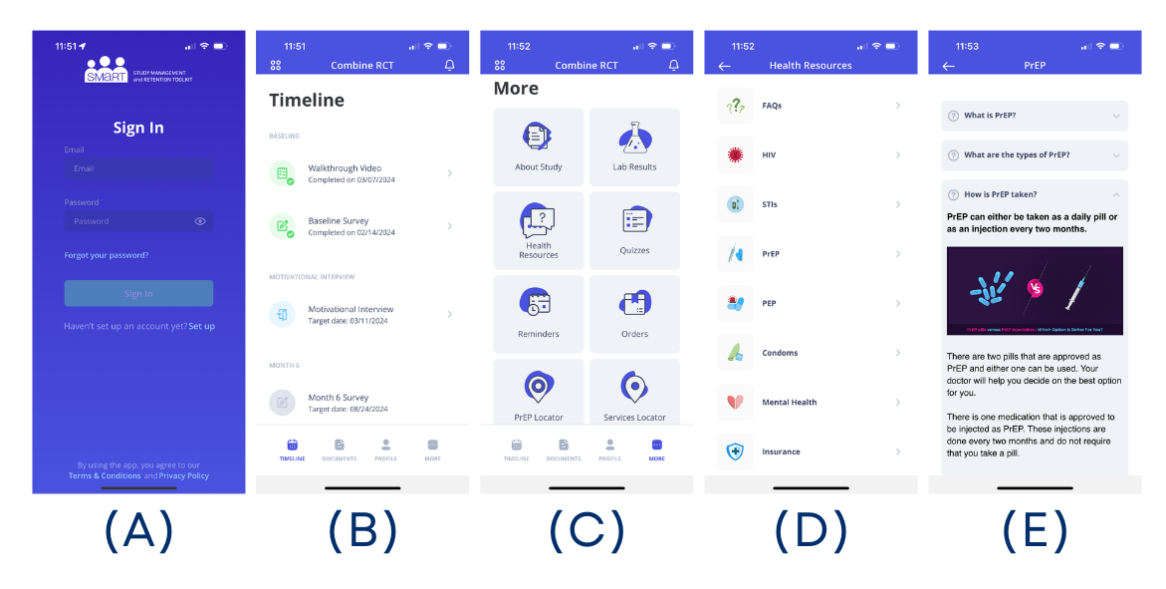
Example screenshots of the Study Management and Retention Tool (SMaRT) web application, including the different Combine intervention content sections: (A) log in screen, (B) study timeline, (C) intervention content home screen, (D), health resources section, (E) frequently asked questions about pre-exposure prophylaxis.

Example screenshots are provided in Figure 2. Once logged in, participants will select the Combine study and will be brought to their unique timeline. From here, they will navigate through the app using the bar across the bottom of the screen to get to other documents, their profile, and more resources. This is where all the intervention content is located. We developed videos and infographics to demonstrate app functionality for participants. Each app section is subsequently described briefly.

#### Timeline

This section includes the major time points of the study (baseline, month 6, month 12, month 18, and month 24). It allows participants to see upcoming and previously completed surveys. A walk-through video of the app is pinned at the top of the timeline so that it can be revisited at any point during the study period.

#### About Study

This section provides participants with a summary of the study’s purpose and scope. Contact information for study staff is provided in this section.

#### Health Resources

This section details app-specific and study-specific information as well as sexual, mental, and medical health information. To engage participants, information is presented as text, graphics, videos, and links to other resources. Resources are broken down into 8 categories and include the following sections:

- Frequently asked questions—general information about the study and app
- HIV—information about HIV transmission, risk factors, testing, symptoms of infection, and treatment options
- STIs—information about STIs, including transmission, risk factors, symptoms, testing, and treatment options
- PrEP—information about PrEP modalities, effectiveness, side effects, cost, payment assistance, and how to find a PrEP provider
- Postexposure prophylaxis—information about postexposure prophylaxis, including indications for use, cost, and how to find a PrEP provider
- Condoms—information about how to use condoms, condom-compatible lubricants, how to make condoms more comfortable, condom use and PrEP, and how to get condoms, including through the Combine app
- Mental health—information about mental health, including definitions of mental health, prevalence of mental health conditions, and how to seek mental health services
- Insurance—information about how to obtain health insurance coverage

#### Reminders

Participants can set custom reminders to prompt them to complete HIV and STI testing on a regular schedule.

#### Locators

Participants have access to 4 service locators within the app:

- PrEPLocator [37]
- HIV and STI testing provider locator
- Substance Abuse and Mental Health Services Administration’s mental health and substance use service provider locator
- US Department of Health and Human Services and Minority HIV/AIDS Fund HIV services locator

#### Orders

Participants can order sexual health resources for free through the app. All participants can order condoms and condom-compatible lubricants. Participants in arms 2 and 3 are also able to order HIV and STI self-test kits.

#### Quizzes

Brief self-assessments are provided to allow participants to monitor their sexual behaviors associated with HIV and STI acquisition and transmission, mental health, and substance use and help determine which testing options are best for them.

#### Laboratory Results

This section provides a secure portal for study staff to deliver STI test results or for participants to upload test results obtained outside of the study.

#### Access to App Content

Most app content is the same for all participants. However, participants in arms 1 and 3 can schedule an MI through the app, and participants in arms 2 and 3 can order free HIV and STI self-test kits through the app.

### Participants and Recruitment

We plan to enroll 464 participants (n=116, 25% per arm). The inclusion criteria are as follows: participants should be cisgender male (ie, assigned male sex at birth and currently identify as male), be aged ≥18 to 45 years, reside in the Southern United States as defined by the US Census Bureau or in Missouri (an Ending the HIV Epidemic rural priority state not in the Southern United States), live in a county classified as rural per the Index of Relative Rurality (IRR [38]; refer to subsequent sections for details), own an Android or iOS phone with active service, be willing to download a study app to their phone, be able to read and understand English, report anal sex with a man in the past 12 months, be of unknown serostatus or tested negative for HIV, and not be currently using PrEP.

Participants who are not able to provide informed consent will be ineligible. We plan to enroll a diverse sample comprising at least 50% (232/464) men of color, with an emphasis on enrolling non-Hispanic Black and Hispanic MSM.

The IRR is a continuous metric used to assign a level of rurality to counties in the United States [38]. The IRR is bounded from 0.0 to 1.0 (inclusive), with higher scores indicating greater rurality. IRR scores for counties in the southern US and the state of Missouri from the year 2020 [39] were used to determine participant rurality. Counties with an IRR score of >0.4 are typically considered to be rural [38]; we used a cutoff of 0.35. We have previously shown that an IRR score of >0.4 is a meaningful threshold with respect to access to and uptake of HIV prevention services [14]. To determine a threshold for enrollment into this study, we also explored disparities in HIV prevention service uptake using an IRR threshold of 0.35 to increase the population of potential participants. We observed similar disparities in HIV testing, STI testing, and PrEP uptake using an IRR threshold of 0.35, so we opted to use this threshold to define rurality. We assessed participant geography using self-reported zip code. IRR is determined at the county level, so we use a validated method [40] to crosswalk self-reported participant ZIP code to county to determine participant rurality for eligibility.

Recruitment will occur primarily online and include various approaches given the constantly evolving nature of online venues and platforms. Common approaches include social networking (eg, Instagram [Meta Platforms, Inc] and Snapchat [Snap Inc]), sexual networking and dating apps (eg, Jack’d [Perry Street Software], Scruff [Perry Street Software], and Grindr), or other websites frequented by MSM (eg, Queerty). These online methods have had previous success in recruiting tens of thousands of MSM into HIV prevention research studies [41-43].

In-person recruitment will occur in collaboration with community-based organizations (CBOs) throughout the recruitment period and include the distribution of recruitment materials (eg, palm cards and flyers) during events or in clinic settings.

Unique tracking links will be used so that we will be able to identify the recruitment source for each participant.

### Screening and Consent

The online screening process begins once a potential participant has clicked on an advertisement and is redirected to our screener, hosted on a HIPAA-compliant platform, Alchemer. This screener landing page includes a brief overview of the study and a screening consent (Multimedia Appendix 1). Potential participants are also able to leave contact information if they do not have time to take the screener or wish to take the screener at another time. Study staff sends each of these individuals a link to the screener so they can complete it at their convenience. The brief screening consent covers points, such as protected health information, what types of questions will be on the screener, and whom to contact regarding questions about the study. Potential participants must agree to the screening consent to move on to the screening survey, which will determine their eligibility status. The screener should not take more than 5 minutes to complete.

After completing the screener, eligible participants are prompted to complete the enrollment consent form (Multimedia Appendix 2). Included in this enrollment consent is a video consent that highlights key aspects of the consent form in an audio and visual format. Once eligible participants have completed the enrollment consent and provided their contact information, they are able to immediately take their baseline survey. If eligible participants prefer to take their baseline survey later, they will be emailed a link instead. Those who are ineligible are provided with links to HIV prevention information and locators to identify testing providers. They are also able to provide their contact information to be contacted about future opportunities to participate in research conducted by Emory University.

### Baseline Survey

The baseline survey is the first of 5 surveys that participants will complete. Because it is the most comprehensive of the surveys, the estimated time to complete the survey is approximately 30 to 45 minutes. Participants receive a US $50 electronic gift card to their preference of Amazon, Target, or Walmart for completing the baseline survey and fraud check (described subsequently). Domains in the baseline survey include demographics, health care access and use, HIV and STI testing history, sexual behavior, PrEP awareness and use, substance use, stigma, and mental health. Select questions are repeated across the screener and baseline surveys for comparison. Any discrepancies in participants’ answers across the 2 surveys are resolved during the fraud check (described in the Fraud Prevention, Enrollment, and Randomization section). The entire instrument is included as Multimedia Appendix 3.

### Fraud Prevention, Enrollment, and Randomization

To avert fraudulent registrations, details of eligibility criteria will not be reported before screening, and screening surveys will not have a back button to discourage users from manipulating the screener for eligibility purposes. The screener survey also includes reCAPTCHA to filter out fraudulent responses. Once an eligible participant has completed their baseline survey, a fraud check is initiated that requires potential participants to complete a fraud check phone call with study staff. During the fraud check call, study staff review any discrepancies between the screener and baseline surveys, verify that participants have downloaded the mySMaRTmobile app, and assist in any troubleshooting for the participants upon logging into the app or creating their account. If participants are unable to adequately explain any discrepancies in their responses across the screener and baseline survey, they are determined to be ineligible. After successfully passing the fraud check, the study staff randomizes and enrolls the participant. Any participants identified as fraudulent after enrollment will be removed from the study and excluded from analyses; enrollment totals will be increased to account for the fraudulently enrolled participants.

Arm assignments were generated using random permuted block randomization. Assignments were unmasked. Those who are randomized to a study arm with one or both of the adjunctive interventions are able to see that content following randomization.

### Follow-Up Surveys

Follow-up surveys are conducted at months 6, 12, 18, and 24, and each survey is expected to take approximately 25 minutes for participants to complete. Participants are compensated US $50 upon completion of each survey with their choice of an Amazon, Target, or Walmart electronic gift card. Domains in follow-up surveys are similar to the baseline survey, with the addition of questions assessing use of HIV tests, STI tests, and PrEP in the previous 6 months and arm-specific questions regarding the acceptability and use of adjunctive interventions. The complete instrument can be found in Multimedia Appendix 4.

### Retention

A preplanned schedule of contact attempts that consists of a variety of follow-up methods will be used to increase retention. Survey reminders are sent 14, 7, and 2 days before the closing of the follow-up surveys. Study staff can record in SMaRT when each activity is completed. Reminders are also sent to participants in the MI arms (arms 1 and 3) if they have not scheduled their interview yet, with instructions on how to do so. Regular messages will also be automatically sent to participants to remind them about different features in the app (eg, locators and product ordering).

Video walk-throughs are available to orient participants to the app and are customized based on individual arm assignment. These videos are easily accessible at the top of the participant’s timeline, beginning the day the participant is enrolled in the study, and will stay there for the entirety of the study period. These brief walk-through videos contain important information about how to access follow-up surveys and the different sections available in the SMaRT web application, including the different Combine intervention content sections.

### Primary Outcomes

The primary effectiveness outcomes are HIV testing, STI testing, and PrEP initiation. All primary outcomes will be measured via self-report on each follow-up survey. The first time a participant reports PrEP use on a follow-up survey, they will be asked to provide a photo upload of their prescription for a US $15 incentive or submit a self-collected dried blood spot sample for a US $40 incentive to verify PrEP use.

The primary implementation outcome is maintenance. App-based interventions provide the opportunity to obtain detailed metrics on participant engagement [44,45]. Even over relatively short time periods, there tends to be a substantial decrease in the use of HIV prevention apps [44]. However, it is difficult to know whether the reduced app use reflects intervention failure [46]. If a user initiates and sustains a regular HIV testing regimen following app use, the testing intervention would be maintained over time despite a lack of engagement with the app. Thus, to fully understand and describe maintenance of the intervention, both app use and effectiveness data are needed over a prolonged time frame. The Reach, Effectiveness, Adoption, Implementation, and Maintenance (RE-AIM) framework defines maintenance at the individual level as sustained intervention use 6 months after initiation [47]. However, this time frame was arbitrary [48] and is too short for an outcome of HIV and STI testing, which are recommended at least annually for most MSM [49,50]. This need for prolonged follow-up motivated the 2-year follow-up time frame for our study. Thus, maintenance of HIV testing will be defined as at least 1 HIV test in the first 12 months and at least 1 test in the final 12 months of follow-up. Similar definitions will be used for maintenance of STI testing and PrEP.

### Statistical Analysis

We will examine the main effects of the motivational interviewing intervention and availability of test kits on the clinical outcomes of HIV and STI testing uptake and PrEP initiation in each year of study follow-up. We will use a multivariable generalized linear mixed model with a log link and binomial outcome distribution to estimate the relative probability of HIV testing uptake comparing those who had the ability to order HIV test kits compared to those who did not and among those who received an MI compared to those who did not. An analogous method will be used for the outcomes of STI testing and PrEP initiation. We hypothesize that there will be main effects of both the MI and access to free test kits. Participants will be analyzed based on their arm assignment (ie, intention-to-treat analysis). Although we will not have the statistical power to assess the interaction between the 2 intervention components, we will conduct exploratory analyses to assess evidence of any synergistic effects.

The final, deidentified dataset will be shared through the Inter-university Consortium for Political and Social Research [51].

### Power Analyses

Power calculations are based on simulations using a binomial model of the form as follows:


log(P(uptake|kit,mi)) = α + β1kit + β2mi

where kit and mi are binomial variables representing the availability of self-test kits and an MI, respectively. We simulated 10,000 trials representing the following underlying assumptions of HIV testing uptake in each arm: 44.8% (52/116) in arm 1 (a slight increase over the baseline estimate of 38% from the American Men’s Internet Survey) and 76.7% (89/116) uptake in arms 2 to 4 (ie, similar effect of kits and MI without interaction). The 76.7% (89/116) uptake (a relative increase of 71% over control) is based on the results of a 4-month pilot of HealthMindr [25], so it is likely an underestimate of expected uptake over 24 months. Assuming α of .05 and a sample size of 376 (n=94, 25% per arm), we will have 93% power to detect an effect of each of the intervention components (ie, self-test kits and MI). The smallest detectable difference (ie, 80% power) will be an uptake of 72.4% (84/116) in the intervention arms. Assuming the aforementioned sample size of 94 per arm, and control uptake of 25% (29/116; a slight increase in the baseline estimate of 22% from the American Men’s Internet Survey), we will have 80% power to detect a relative increase of 112% in STI testing (uptake: 61/116, 52.6% in arms 2-4). The uptake of STI testing in the HealthMindr pilot was 47% without STI self-testing [25], so this is also likely an underestimate. Less reliable data are available for the prevalence of PrEP use among rural MSM; however, only 11% of rural MSM in the Southern United States have ever used PrEP [15]. Assuming the current prevalence of PrEP use is 5% (ie, half of those who have ever used PrEP are currently using PrEP), we will have 80% power to detect an absolute difference in PrEP use of 29% (assumptions: 6/116, 5.2% in arm 1 and 34/116, 29.3% in arms 2-4; risk ratio: 5.1). With respect to maintenance of the intervention, in the HealthMindr pilot study, 22.9% (25/109) of the participants used the app in the fourth (and final) month of follow-up [25]. We used the simulation-based method described earlier to determine power to detect a similar absolute increase in intervention maintenance as we proposed for the effectiveness outcome (ie, 28 percentage point increase; 127% relative increase). We will have 80% power to detect continued engagement with the app (ie, maintenance) of ≥50%. We anticipate that the hypothesized referent maintenance level of 22% is high because it is based on only 4 months of follow-up, so this is a conservative estimate of power. These power calculations assume a single observation per participant; however, our longitudinal design will result in increased power to detect a difference between treatment arms [52]; therefore, our estimates of power are conservative. To allow for 10% attrition per year, we will enroll a total sample of 464 (n=116, 25% per arm) participants.

### Cost and Cost-Effectiveness Analyses

We will conduct comprehensive analyses to assess the cost of developing and implementing the interventions using a microcosting approach. We will use standard economic evaluation methods as recommended by the US Panel on Cost-effectiveness in Health and Medicine [53-55] and adapted for HIV and AIDS programs by Jain et al [56]. These methods have been used for economic analyses for a variety of recruitment, HIV testing, and linkage to care interventions, including for MSM in the United States [57-59]. The economic analysis will be conducted from the societal perspective (the cost to the party implementing the program plus the cost to the men participating in the program) to estimate the cost and cost-effectiveness of the innovative strategies to promote repeat HIV testing among MSM using the Combine app relative to the comparison strategy. The societal perspective accounts for costs to all parties, acknowledges the value of competing uses for society’s resources, and maximizes comparability with other cost-effectiveness analyses. Data will be entered into a standardized Microsoft Excel spreadsheet with embedded formulas to calculate costs similar to publicly available data collection and costing spreadsheets used in similar studies [57-59]. The implementation strategy costs will not be discounted to aid in budget impact analyses. Cost information will be abstracted from accounting records, study surveys, published literature reports, and electronic time stamps.

Cost-effectiveness analyses will include calculating the incremental cost-effectiveness ratio (ICER) for each arm compared to standard of care as follows:


[(Cost_Intervention – Cost_StandardofCare)] / [(Outcome_Intervention – Outcome_StandardofCare)]

ICERs will be calculated for each primary outcome (ie, HIV testing, STI testing, and PrEP initiation). These comparisons will help implementers weigh the relative costs and benefits of each strategy. Costs and effects will be discounted at 3% per year [53,54]. One-way sensitivity analyses for all input variables will determine which inputs the ICERs are most sensitive to. We will also conduct a sensitivity analysis excluding development costs under the assumption that the implementation strategies could be rolled out without significant investment in development. Intervention cost-effectiveness will be estimated over the 2-year time horizon of the trial.

### MI Session

#### Overview

Participants in arms 1 and 3 will have the opportunity to participate in an MI. The MI session, delivered via HIPAA-compliant Zoom (Zoom Communications, Inc), is designed to promote engagement with HIV and STI testing and other prevention behaviors using the Combine app. A systematic review identified 8 behavioral change techniques that have demonstrated impact on HIV and STI behavioral change, including problem solving, feedback on behavior, social support, instructions on how to perform the behavior, information about health consequences, information about social and environmental consequences, demonstration of the behavior, and a credible source. These 8 behavioral change techniques form the foundation of the MI session. Specifically, the MI aims to achieve the following: (1) increase insight into the Combine app utility and personal HIV risk and prevention behaviors, (2) enhance motivation and empowerment to make sexual health behavior decisions, (3) align participants’ values and behavior goals to increase commitment for potential change, and (4) operationalize an HIV and STI prevention plan based in individual values and goals and leveraging the study app. A comprehensive MI procedure and quality assurance manual was prepared by a licensed clinical health psychologist and study team member with expertise in MI and HIV (TRG).

The Combine MI manual is used to guide each interview to ensure that the developed curriculum is provided to each participant according to protocol. The MI comprises 4 stages, outlined in Textbox 1.

**The 4 stages of the motivational interview (MI).**

**Phase 0: consent and agenda setting**

- The purpose of this stage is to mitigate any anticipatory distress by presenting what the discussion will cover. Verbal consent for recording is documented at this stage, as consent for the MI itself has already been discussed.

**Phase 1: overview and demonstration of the Combine app**

- The purpose of this stage is to orient the user to the app, highlight key HIV prevention functions, and provide a foundation for the discussion of HIV prevention planning.
- Behavioral change techniques include instructions on how to perform the behavior and demonstration of health behavior.

**Phase 2: HIV prevention counseling with MI-based problem-solving**

- The purpose of this stage is to help the participant identify recent HIV risk behaviors, identify potential HIV prevention strategies, reinforce recent HIV prevention efforts, solve problems concerning barriers to HIV prevention, and point out which app features can be used to help their HIV prevention efforts. Grounding the discussion in the app both encourages app use and makes it clear to the participant that they have a resource in their hand that they can use to enact the prevention plan they are building with the interventionist.
- Behavioral change techniques include feedback on behavior, information about health consequences, information about social and environmental consequences, credible source, problem-solving, and social support.

**Phase 3: action plan**

- The purpose of this stage is to build an individualized plan for engagement in HIV prevention (by picking 2 specific behaviors aligned with personal values and goals) within the context of the app to increase the likelihood of follow-through with their action plan.
- Behavioral change techniques include feedback on behavior and instructions on how to perform the behavior.

Participants who complete the MI will earn a US $25 electronic gift card incentive upon completion of the interview. The interview should occur within the first 60 days of participant enrollment, with reminders sent to participants via text and email. If the participant fails to schedule their interview, staff will continue to send monthly reminders for 12 months. Participants can schedule their interview at any point during their enrollment. The calendar function within the timeline of the Combine app will allow participants to self-select times for their MI, which are updated monthly and monitored daily by study staff based on their availability. Once a participant selects an available time slot, the SMaRT system will notify staff of the appointment selection, and staff can then approve or deny the appointment and send a confirmation message, which includes the Zoom link to the meeting.

Interviewers complete a training program, including readings, a multiday seminar, mock interviews with feedback, and, once assessed to be competent, individual supervision with feedback on 2 initial MI sessions. Quality assurance shall be performed on 19.8% (46/232) of all sessions by trained staff by completing data collection forms and listening to audio recordings for any issues, concerns, or questions. Sessions that will undergo review will be randomly selected on an ongoing basis from batches of approximately 10 visits. Quality assurance documents include a “session fidelity checklist,” which ensures that topics from the Combine MI manual are discussed, and global scales from the *Motivational Interviewing Treatment Integrity Coding Manual 4.2.1* [60], which cover themes of cultivating change talk, softening sustain talk, and fostering partnership and empathy. Forms must score an interrater reliability of >80%. If agreement is <80%, remediation (eg, rerate under supervision, conduct group discussion, and retrain the rater) will occur.

#### In-Depth Interviews

At the end of the follow-up period, study staff will conduct in-depth interviews (IDIs) with some participants. IDI participants will be randomly selected from a sampling frame of all participants that is stratified by app engagement. In this way, we will purposefully sample from participants who used the app at least once in each 6-month follow-up period and those who used the app less frequently. These IDIs will focus on the features of the app that participants found most and least useful and the factors that might influence them to use an HIV prevention app outside the context of a research study. The IDI will begin by asking the participant to walk the interviewer through a “typical use session” of the app during which the interviewer will probe into key decisions (eg, why one function was used over other functions). The interview will examine the participant’s views on the positive and negative elements of the app. Specifically focusing on the RE-AIM domain of maintenance, the interviewer will probe reasons for continued use among those with high engagement, reasons for not using the app among those with low engagement, and strategies for improving maintenance. The goal of these interviews will be to develop actionable items that we can incorporate with quantitative data from follow-up surveys, app paradata, and qualitative data from stakeholders to further refine implementation strategies specifically tailored for rural MSM.

During IDIs with key stakeholders at health departments and CBOs, we will explore considerations around implementation within the domains of RE-AIM and strategies from the Expert Recommendations for Implementing Change project [61]. The goal of these discussions will be to identify common themes and actionable items to support future implementation of app-based HIV prevention interventions in community settings. Interviewees will first be asked to describe current programs to support HIV testing among rural MSM and their experience with mHealth interventions in general and rural MSM specifically. Depending on their previous experience, interviewees will be asked to describe actual or hypothetical barriers to implementing mHealth interventions for rural MSM. Interviewees will also be probed for strategies they have successfully used to reach rural MSM in the past and ideas for adapting those strategies in mHealth interventions. Other key aspects of implementation and maintenance that will be probed include technological capabilities of the implementers, additional capacity building that might be necessary to support an mHealth intervention, and strategies for supporting sustained use. Example IDI questions for stakeholders are included in Table 1.

**Table 1:** Sample in-depth interview questions for stakeholders.

Domain	Questions
Reach	How do you engage clients in HIV and STI^a^ testing interventions? What proportion of your target population are you able to engage in HIV and STI testing? How could an app-based intervention affect your ability to reach MSM^b^ for HIV and STI testing?
Effectiveness	What type of effectiveness data would convince your organization to adopt a new intervention?
Adoption	What strategies have you used in the past to encourage adoption of interventions among your health care providers and staff? Would cost data be useful for justifying allocating resources to an app-based intervention? What type of cost data are used to make decisions in your organization?
Implementation	What types of changes might be needed in your infrastructure to support the implementation of an app-based intervention? What type of support might you need to increase capacity to support app-based interventions?
Maintenance	What is your capacity to support the prolonged use of app-based interventions among your clients? What type of support would you need to build on this capacity?

^a^STI: sexually transmitted infection.

^b^MSM: men who have sex with men.

### Qualitative Analysis

All IDIs will be digitally recorded, transcribed verbatim, and deidentified. Transcripts will be entered into MAXQDA (VERBI Software), which facilitates the processes of coding, annotating, and retrieving text such that analysts may note patterns in the textual data across themes. Data analyses will be conducted using a phenomenological inquiry framework [62,63]. Phenomenology is focused on describing what people within a group have in common as they experience a phenomenon and is an inductive analytic approach that allows the patterns, themes, and categories of analysis to emerge from data [62,63]. After close readings of the text, the lead analysts will create a preliminary codebook to capture emergent themes. We anticipate developing a codebook consisting of 12 to 15 codes representing key themes, including explicit domains from the interview guides and the RE-AIM domains (deductive themes) and pervasive, unanticipated themes that were emergent across various transcripts (inductive themes). Provisional definitions will be given to each code. The preliminary codebook will be presented to other analysts (per the phenomenological approach), and a group discussion will occur. After edits are made to the codebook, all analysts will apply the preliminary codebook to a single transcript. The coded transcripts will be merged for comparison, and code definitions will be revised based on an examination of coding disagreements. This process will be repeated until consistent agreement is attained among all coders. A finalized codebook will be created, and the trained analysts will start coding (from scratch) all the textual data for the project. The goal of this analysis will be to describe the key factors anticipated to affect future implementation of the Combine app in real-world settings and inform the development of implementation strategies to support optimal implementation of the Combine app for rural MSM.

### Dissemination Plan

Trial results will be disseminated through publications in scholarly journals and direct communications with research participants and community partners. The study team has developed an authorship agreement that outlines expected roles on primary and secondary result papers.

### Ethical Considerations

This study has been reviewed and approved by the institutional review board at Emory University (STUDY00006280) and has been registered at ClinicalTrials.gov (NCT06205368). This is a low-risk trial, and adverse events are not expected; however, any adverse events that arise will be reported to the sponsor and funding agency as required. All protocol modifications will be approved by the institutional review board before implementation and will be reported to the funding agency and updated on ClinicalTrials.gov.

All participants will provide informed consent to complete the eligibility screener and enroll in the study. Data are not deidentified or anonymized. All data are maintained on secure servers that require password access. All participants are compensated for completing study activities. Participants are compensated US $50 for each of the baseline and follow-up surveys. Participants who report PrEP initiation are invited to submit a photo of their prescription (US $15 incentive) or a self-collected dried blood spot sample (US $40 incentive) as verification. Those who participate in MIs will receive US $25 and those who participate in IDIs will receive US $60. All compensation is provided in the form of an electronic gift card.

## Results

Participant enrollment began in March 2024. As of July 2025, 395 participants have been enrolled and randomized (arm 0: n=100, 25.3%; arm 1: n=97, 24.6%; arm 2: n=98, 24.8%; arm 3: n=100, 25.3%). Of those who have been eligible to complete a 6-month follow-up survey so far, 81.3% (122/150) have done so. Recruitment is projected to be completed by December 2025; participant follow-up will be completed 2 years later. Study results are expected to be available in 2028.

## Discussion

### Anticipated Findings

MSM in the rural Southern United States face numerous obstacles to accessing HIV prevention services, including limited to no access to culturally competent care, enhanced experiences of stigma, and concerns of sexual identity disclosure [22,64-66]. Although rural MSM have expressed interest in and willingness to use mobile technology to access HIV testing, STI testing, and PrEP [19,20], large-scale studies have not yet demonstrated the effectiveness of mHealth interventions to increase access to these services among rural MSM. Thus, the goal of this study is to assess the effectiveness of the Combine intervention, based on the HealthMindr platform, to increase uptake of these services while also assessing the modifying effects of 2 adjunctive interventions—free, at-home self-test kits and an MI. Using a 2×2 factorial randomized design, we will be able to answer questions not only about intervention effectiveness but also the necessary components of the intervention to achieve optimal HIV prevention outcomes.

Specifically, the factorial design will allow us to assess the independent effects of including free HIV and STI test kits and an MI to increase HIV prevention service uptake. Although financial constraints prohibit enrolling a study population large enough to facilitate assessing the interaction between test kit availability and the MI, we will assess the interaction effects in secondary analyses. This knowledge will not only inform future intervention implementation for HIV prevention among rural MSM but also will likely generalize to nonrural settings.

### Limitations

This study has limitations. Due to cost constraints, the app is only available in English. Although a Spanish-language version of the app would be ideal, it is currently cost prohibitive. There is a substantial Latino and Hispanic population in the rural Southern United States. However, we expect most Hispanic and Latino MSM will be eligible because, as of 2014, 76% of Latino people in the United States in the proposed age range for this study were proficient in English [67]. The primary outcomes are measured via self-report and are therefore subject to recall and reporting bias. To mitigate social desirability bias, participants complete surveys online and are encouraged to complete the survey in a private place. The study is also limited to cisgender men. Although there are other priority populations who could benefit from a similar intervention, the language in the app is tailored to cisgender MSM. Engaging other communities in this study without properly adapting and modifying the app would lead to potentially stigmatizing experiences. We have previously found an adapted version of HealthMindr to be acceptable among transmasculine people [68], and future research should continue to explore adaptations among other priority groups. Participants will be followed for 2 years to allow for multiple opportunities to test under current recommendations from the Centers for Disease Control and Prevention [49], which presents challenges with respect to participant retention. We have incorporated multiple touchpoints along the way to keep participants engaged and believe that the incentive structure for surveys will encourage retention. In addition, the same barriers that prevent HIV prevention service uptake among rural MSM might also limit willingness to participate in an HIV prevention study. We have successfully recruited rural MSM in the past [34], and we will work diligently with CBOs to successfully meet recruitment goals. Finally, mHealth interventions pose unique challenges with respect to the ever-evolving technology landscape. We have an ongoing relationship with our software developer and have budgeted for anticipated modifications to ensure that the app continues to work with future releases of the Android and iOS operating systems.

This study will provide critical data on the effectiveness of and key implementation factors necessary to support mHealth interventions to increase HIV testing, STI testing, and PrEP use among rural MSM in the Southern United States.

## Appendix

Multimedia Appendix 1 Screening informed consent form.

Multimedia Appendix 2 Enrollment informed consent form.

Multimedia Appendix 3 Baseline survey.

Multimedia Appendix 4 The 6-month follow-up survey.

Multimedia Appendix 5 SPIRIT (Standard Protocol Items: Recommendations for Interventional Trials) checklist.

## Abbreviations

CBO: community-based organization
HIPAA: Health Insurance Portability and Accountability Act
ICER: incremental cost-effectiveness ratio
IDI: in-depth interview
IRR: Index of Relative Rurality
mHealth: mobile health
MI: motivational interview
MSM: men who have sex with men
PrEP: pre-exposure prophylaxis
RCT: randomized controlled trial
RE-AIM: Reach, Effectiveness, Adoption, Implementation, and Maintenance
SMaRT: Study Management and Retention Tool
STI: sexually transmitted infection

## Data Availability

The datasets generated or analyzed during this study are available from the corresponding author on reasonable request.
