# Supplementary material for: Evaluating the Effectiveness of a Mobile HIV Prevention App to Increase HIV and Sexually Transmitted Infection Testing and Pre-Exposure Prophylaxis Initiation Among Rural Men Who Have Sex With Men in the Southern United States: Protocol for a Randomized Controlled Trial
Source: JMIR Res Protoc. 2025 Jul 23;14:e69540. doi: 10.2196/69540 (PMC12329388; doi:10.2196/69540)
Supplement: Multimedia Appendix 1 [file resprot_v14i1e69540_app1.docx]

**Emory University**

**Screening Consent Information Sheet**

**For Research Study Screening**

**Title**: Evaluating the effectiveness of a mobile HIV prevention app to increase HIV and STI testing and PrEP initiation among rural men who have sex with men

**IRB #:** STUDY00006280

**Principal Investigator:** Jeb Jones, PhD, MPH, MS, Department of Epidemiology, Rollins School of Public Health, Emory University

**Study-Supporter:** National Institute of Mental Health

## Introduction and Study Overview

Thank you for your interest in The Combine Study. To see whether you may be a candidate for this study, we need to ask you for some information about yourself. But first, let me tell you about this screening interview and what we will do with your information.

1. This screening interview will take about 5 minutes.
2. You can also stop the screening interview at any time. This is completely voluntary.
3. We can send you an information sheet about this screening, along with the screening questions, if you would like. We will also give you a form you can send in later if you change your mind and want us to remove your information from our records.
4. We will ask you about your demographic background, sexual behavior, and history of HIV testing and will record this information in a database containing information from others who have also shown interest in the study.
5. This information will only be used for the research study you are interested in.
6. The only risk to you in this online screening is a potential loss of privacy. However, your privacy is very important to us, and we will be very careful with your information.
7. Your health information that identifies you is your “protected health information” or “PHI.” We will use your PHI to screen you for our research study.
8. The PHI we will use includes your HIV testing history.
9. To protect your PHI, we will follow federal and state privacy laws, including the Health Insurance Portability and Accountability Act (called HIPAA or “the Privacy Rule” for short).
10. The following persons or groups may use and/or disclose your PHI for this study:

- The Principal Investigator and the research staff
- National Institutes of Health, who funds this Research, and people or companies they use to carry out the study
- Emory offices who are part of the Human Research Participant Protection Program
- Any government agencies who regulate the research including the Office of Human Subjects Research Protections

1. We will disclose your PHI when required to do so by law in the case of reporting child abuse or elder abuse.
2. You may revoke your authorization at any time by calling the principal investigator, Jeb Jones.
3. If identifiers (like your name, address, and telephone number) are removed from your PHI, then the remaining information will not be subject to the Privacy Rules. This means that the information may be used or disclosed with other people or organizations, and/or for other purposes.
4. We do not intend to share your PHI with other groups who do not have to follow the Privacy Rule, but if we did, then they could use or disclose your PHI to others without your authorization. Let me know if you have questions about this.
5. Your authorization will not expire because your PHI will need to be kept indefinitely for research purposes.
6. We can send you a copy of this information, if you would like.

## Contact Information

## If you have questions about this study, your part in it, or if you have questions, or concerns about the research you may contact the following:

## Jeb jones, Principal Investigator: 404-712-2275

##

This study has been reviewed by an ethics committee to ensure the protection of research participants. If you have questions about your **rights as a research participant**, or if you have **complaints** about the research or an issue you would rather discuss with someone outside the research team, contact the Emory Institutional Review Board at 404-712-0720 or 877-503-9797 or [irb@emory.edu](mailto:irb@emory.edu).

You can also stop the screening interview at any time. This is completely voluntary.

## Consent

Do you agree to participate in the screening process, and authorize the use and disclosure of your protected health information as described above?

Participant agrees to participate: Yes No

If Yes:

Thank you for your interest. Please answer the following questions to determine if you are eligible for this study. [Go to eligibility screener.]

OR

Thank you for taking the time to complete this survey. We are sorry you are not interested in our study. If you have any concerns or questions, or would like to be considered for our study, please email us at [jeb.jones@emory.edu](mailto:jeb.jones@emory.edu) or call us at 404-712-2275.
