# Supplementary material for: Evaluating the Effectiveness of a Mobile HIV Prevention App to Increase HIV and Sexually Transmitted Infection Testing and Pre-Exposure Prophylaxis Initiation Among Rural Men Who Have Sex With Men in the Southern United States: Protocol for a Randomized Controlled Trial
Source: JMIR Res Protoc. 2025 Jul 23;14:e69540. doi: 10.2196/69540 (PMC12329388; doi:10.2196/69540)
Supplement: Multimedia Appendix 2 [file resprot_v14i1e69540_app2.docx]

**You Are Being Asked to Be in a Research Study**

**Concise presentation of key concepts**

You are being asked to be in a research study. A research study is designed to answer a scientific question. If you agree to be in the study, you will be one of 464 people who are being studied at Emory.

**Why is this study being done?**

This study is being done to answer the question: What is the most effective way to increase testing for HIV and sexually transmitted infections among gay and bisexual men? You are being asked to be in this research study because you are a man who has sex with other men and you live in a rural area of the southern United States.

**Do you have to be in the study?**

It is your choice to join this research study. You do not have to be in it. Before you choose, take time to learn about the study.

**What do you have to do if you choose to join this study?**

If you qualify and choose to join the study, you will participate for 2 years. All study activities will be completed online or by phone. There will be a total of 5 surveys for you to complete. The researchers will ask you to do the following: Download an app to your smartphone and take occasional online surveys. Some participants will also be asked to participate in an interview. If chosen, you will be asked to consent separately for the interview. The purpose of the interview will be to collect more information on your experiences as a participant on the study and the app.

**How is this study going to help you?**

If you are in the study, you will be helping the researchers answer the study question. This study is not intended to benefit you directly. However, you might get access to free HIV and STI test kits based on your participation in the study.

**What are the risks or discomforts you should know about before deciding?**

The study will take time. All studies have some risks. Some risks are relatively small, like being bored or losing time. Some are more serious. Risks for this study include:

- loss of privacy
- breach of confidentiality

You can find a full list of expected risks, their frequency and severity in the section titled “What are the possible risks and discomforts?”

**Alternatives to Joining This Study**

Since this is not a treatment study, the alternative is not to participate.

**Costs**

There will be no costs to you for participating in this study. You will not be charged for any of the research activities.

There is more information in the “Costs” section further below.

**What Should You Do Next?**

Read this form, or have it read to you. Make sure the study staff explains the study to you. Ask questions such as how much time you will have to spend on the study, any words you do not understand and more details about study procedures. Take time to think about this and talk about it with your family and friends.

**Emory University**

**Consent to be a Research Subject**

**Title**: Evaluating the effectiveness of a mobile HIV prevention app to increase HIV and STI testing and PrEP initiation among rural men who have sex with men

**IRB #:** STUDY00006280

**Principal Investigator:** Jeb Jones, PhD, MPH, MS, Department of Epidemiology, Rollins School of Public Health, Emory University

**Study-Supporter:** National Institute of Mental Health

## Introduction

You are being asked to be in a medical research study. This form tells you what you need to think about before you choose if you want to join the study. **It is your choice. If you choose to join, you can change your mind later and leave the study.** Your choice will not cause you to lose any medical benefits. If you choose not to join this study, your doctor will still treat you.

Before you decide:

- Read this form or have it read to you
- Listen carefully to the study staff explain the study to you in the online video
- Ask questions about anything that is not clear

You will get a copy of this form. Take your time to think about joining the study. You may wish to discuss it with family or friends. Do not sign this form if you still have questions or something does not make sense to you. By signing this form, you will not give up any legal rights.

A description of this clinical trial will be available on http://www.ClinicalTrials.gov, as required by U.S. law. This Web site will not include information that can identify you. At most the Web site will include a summary of the results. You can search this Web site at any time.

This trial will be registered and may report results on www.ClinicalTrials.gov, a publicly available registry of clinical trials.

## What is the purpose of this study?

The purpose of this study is to understand the best ways to increase testing for HIV and other sexually transmitted infections (STIs) among gay and bisexual men who live in rural areas of the southern United States. All participants in the study will download a smartphone app that will encourage them to get tested for HIV and STIs. Some participants will also have access to free test kits to test for HIV and STIs at home and some participants will have access to an online counseling session to help them develop a plan to test for HIV and STIs.

**How will we determine eligibility and prevent fraud?**

Eligibility is determined based on key criteria related to the study. Participants determined to be eligible are not yet enrolled into the study and will need to complete a brief verification step with a study member after completion of the baseline survey. You may be asked to complete the verification step through a brief phone call or zoom call (with a staff member using a HIPAA-compliant Emory zoom account). You may also be asked to show or upload a government-issued or school-issued photo ID through our secure survey platform. All of your data is protected under a Certificate of Confidentiality (see below). Successful completion of the verification process is necessary to be enrolled in the study and receive compensation for the baseline survey.

## What will you be asked to do?

If you choose to be in this study, we will ask you to pass a brief verification process (as mentioned above) and then participate in a series of activities over the course of 2 years. These activities will include downloading and interacting with a smartphone app and filling out surveys about your sexual health and behavior. The surveys will take place every six months and will take approximately 30-45 minutes to complete each time.

You may also be asked to participate in a virtual video chat with a study team member. The purpose of the video chat will be to help you develop strategies to deal with any challenges that might prevent you from testing for HIV or sexually transmitted infections (STIs).

You will be asked to participate in a randomized control trial. Randomization means that you will randomly be placed into one of four groups, like flipping a coin. Each group will have access to the smartphone app. The app will have resources related to HIV and STI testing. Some groups will also be able to order free HIV and STI self-test kits. Some groups will have access to video chat with study staff to help them develop a plan to regularly test for HIV and STIs.

You will be able to use the app to order condoms and lube at no cost to you. Some participants will also be able to order HIV and STI test kits at no cost. The STI home testing kits screen for syphilis and oral, urethral, and rectal chlamydia and gonorrhea. Ordering these services is optional. If you do choose to order an STI testing kit, study staff will contact you to deliver results and refer you to treatment services if you test positive for an STI. If you have a positive test for HIV or an STI, state law requires us to report that positive test to the state health department for purposes of statistics and service planning. It is possible that the Health Department could contact you to offer referrals for care or help with getting your partners tested. These procedures are the same as if you were tested at a doctor’s office or a clinic outside of this research study.

Specimen collection kits: If you choose to order HIV and/or STI test kits, you will also receive printed and video instructions on self-administered specimen collection methods, including urine collection, rectal and throat swabs, and finger-prick blood collection. You will be provided with a 24/7 call-in line to receive help with any unexpected problems. Collecting the specimens will take you at most one hour.

After collecting the samples, you will mail them to a certified laboratory in a prepaid mailer included in the kit. Your samples will be used to conduct tests for gonorrhea, chlamydia, and syphilis. Your STI test results will be returned by study staff. Urine samples you provide may be stored and used for additional tests for research purposes only. We will not sell your samples.

Our syphilis test is performed with a standard syphilis test and determines a preliminary positive result. It does not provide detailed results like a similar test from a doctor’s office or health department. Therefore, if you test preliminary positive for syphilis, we will recommend that you seek additional testing from either your doctor or health department.

## Who owns your study data and samples?

## If you join this study, you will be donating your samples and data. You will not be paid if your samples or data are used to make a new product. If you leave the study, the data and samples that were already collected may still be used for this study. If you choose to withdraw from the study at any time, you may request that we destroy any data that we have already collected from you.

## What are the possible risks and discomforts?

The most common risks and discomforts expected in this study are:

You may be uncomfortable answering personal questions, or feel uncomfortable with the HIV prevention topics, on the online surveys. You will have the option to not answer any question that makes you uncomfortable.

The less common risks and discomforts expected in this study are:

There is some risk that you could be observed if you complete the surveys in public, or that data could be at risk during transmission. At the beginning of each survey we will remind you to complete the survey in a private place, where your responses cannot be observed by others. We will also encourage you to delete any emails or text messages received as part of the study to protect them from being seen by anyone else.

Researchers may learn something new during the study that may affect your choice to be in the study. If this happens, they will tell you about it. Then you can choose if you want to stay in this study. You may be asked to sign a new form if you choose to stay in the study.

## Will you benefit from the study?

You may not benefit from joining the study. This study is designed to learn more about how to increase HIV and STI testing among gay and bisexual men in the southern United States. The study results may be used to help others in the future.

##### Will you be paid for your time and effort?

You will get $50 for each completed survey, for your time and effort. If you do not finish the study, we will compensate you for the surveys you have completed. You will get $250 total, if you complete all study visits.

If you are assigned to a group that receives a motivational interview, you will earn $25 for your time and effort if you complete the motivational interview with study staff. This is a one-time activity within the first 60 days of enrollment for those assigned to this group.

Some participants might be asked to complete additional steps based on their responses to the surveys. Participants might be asked to submit photo verification of HIV and STI testing or prescriptions for pre-exposure prophylaxis (PrEP).

A one-time compensation of $15 will be given for a photo upload.

Additionally, participants will have the opportunity to receive $40 for successfully completing and submitting a DBS test card if PrEP is initiated or re-initiated. This incentive is only available up to two times across the participant’s entire study period.

##### What are your other options?

If you decide not to enter this study, there are services available to you outside of this research study. You should discuss this with the researchers if you have concerns or want to know about other options. You may wish to research other study options at websites like clinicaltrials.gov and ResearchMatch.org.

**How will your private information be protected?**

Whenever possible, a study number, rather than your name, will be used on study records. Your name and other identifying information will not appear when we present or publish the study results.

# Certificate of Confidentiality

There is a Certificate of Confidentiality from the National Institutes of Health for this Study. The Certificate of Confidentiality helps us to keep others from learning that you participated in this study. Emory will rely on the Certificate of Confidentiality to refuse to give out study information that identifies you. For example, if Emory received a subpoena for study records, it would not give out information that identifies you.

The Certificate of Confidentiality does not stop you or someone else, like a member of your family, from giving out information about your participation in this study. For example, if you let your insurance company know that you are in this study, and you agree to give the insurance company research information, then the investigator cannot use the Certificate to withhold this information. This means you and your family also need to protect your own privacy.

The Certificate does not stop Emory from making the following disclosures about you:

- Giving state public health officials information about certain infectious diseases,
- Giving law officials information about abuse of a child, elderly person or disabled person.
- Giving out information to prevent harm to you or others.

Giving the study sponsor or funders information about the study, including information for an audit or evaluation.

**Storing and Sharing your Information**

We will store all the data that you provide using a code. We need this code so that we can keep track of your data over time. This code will not include information that can identify you (identifiers). Specifically, it will not include your name, initials, date of birth, or medical record number. We will keep a file that links this code to your identifiers in a secure location separate from the data.

We will not allow your name and any other fact that might point to you to appear when we present or publish the results of this study.

Your data may be useful for other research being done by investigators at Emory or elsewhere. We may share the data, linked by the study code, with other researchers at Emory, or with researchers at other institutions that maintain at least the same level of data security that we maintain at Emory. We will not share the link between the study code and your identity.

We may also place data in public databases accessible to researchers who agree to maintain data confidentiality, if we remove the study code and make sure the data are anonymized to a level that we believe that it is highly unlikely that anyone could identify you. Despite these measures, we cannot guarantee anonymity of your personal data.

**Returning Results to Participants/Incidental Findings**

**Depending on which study group you are assigned to, you might have the ability to order free HIV and STI self-test kits. If you order and return these kits, we will inform you of the results of your test.**

## Costs

There will be no costs to you for participating in this study, other than basic expenses like transportation. You will not be charged for any of the research activities.

## Withdrawal from the Study

You have the right to leave a study at any time without penalty.

If you leave the study before the last planned study visit, the researchers may ask you to complete some of the final steps.

The researchers also have the right to take you out of the study without your consent for any reason. They may do this if they believe it is in your best interest or if you do not agree to changes that may be made in the study.

**Contact Information**

If you have questions about the study procedures, appointments, or other questions or concerns about the research or your part in it, contact Jeb Jones at 404-712-2275.

This study has been reviewed by an ethics committee to ensure the protection of research participants. If you have questions about your **rights as a research participant**, or if you have **complaints** about the research or an issue you would rather discuss with someone outside the research team, contact the Emory Institutional Review Board at 404-712-0720 or 877-503-9797 or [irb@emory.edu](mailto:irb@emory.edu).

To tell the IRB about your experience as a research participant, fill out the Research Participant Survey at [**https://tinyurl.com/ycewgkke**](https://tinyurl.com/ycewgkke)**.**
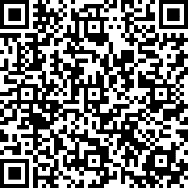


## Consent and Authorization

Being in this study is entirely your choice. You have the right to refuse to participate or to stop taking the survey at any time. Please print a copy of this form for your records.

I understand that checking this box constitutes a legal signature confirming that I have read the consent form and agree to participate in the Combine RCT study. *

- Legally sign document
- Do NOT legally sign document
