# Supplementary material for: Evaluating the Effectiveness of a Mobile HIV Prevention App to Increase HIV and Sexually Transmitted Infection Testing and Pre-Exposure Prophylaxis Initiation Among Rural Men Who Have Sex With Men in the Southern United States: Protocol for a Randomized Controlled Trial
Source: JMIR Res Protoc. 2025 Jul 23;14:e69540. doi: 10.2196/69540 (PMC12329388; doi:10.2196/69540)
Supplement: Multimedia Appendix 3 [file resprot_v14i1e69540_app3.docx]

# COMBINE RCT Baseline Survey

## Dates and Pulled Variables

## SGUID Check

## Landing Page / Intro

### Welcome to the Combine study! Please complete the following survey. It will take you about 30-60 minutes to complete. Once we verify that your survey is valid, you will receive a $50 electronic gift card to your choice of Amazon, Target, or Walmart. If you have any questions, please email [combine_rct@emory.edu](mailto:combine_rct@emory.edu) or call 470-870-6809. We are going to be asking questions about various topics, including your background, life experience, healthcare, your sexual partners and relationships, and substance use. You are not required to answer any questions in this survey. If you do not answer a question, you may receive a prompt to be sure that you meant to leave the question unanswered before you move forward. This is a forward-only survey. When you finish a page, continue to the next page by clicking the "Next" button. You may not go backwards to pages you have already completed. We understand that having a survey without a “back button” can be difficult because it does not allow you to make corrections to your answers. There will be a question at the end of the survey to let us know of any corrections you would like to make. If you feel like you have made a mistake, you can also contact the study team ([combine_rct@emory.edu](mailto:combine_rct@emory.edu)). Your privacy is important to us. All information you provide in this survey will be held confidentially. Your answers will be used only for research purposes.

## GIF1

### ****Let's get to it!****

###

## Demographics

Validation: Min = 12 Max = 120 Must be numeric Whole numbers only Positive numbers only Max character count = 3

Shortname / Alias: age

### ****How old are you?*****

_________________________________________________

**Logic: Show/hide trigger exists.**

Shortname / Alias: country_born

#### ****Where were you born?****

( ) In the United States

( ) Outside the United States - U.S. Territory (e.g., Puerto Rico, U.S. Virgin Islands, Guam) or foreign country

**Logic: Hidden unless: #2 Question "Where were you born?" is one of the following answers ("Outside the United States - U.S. Territory (e.g., Puerto Rico, U.S. Virgin Islands, Guam) or foreign country")**

Shortname / Alias: outsideus_country_born

#### ****In what country or territory where you born?****

( ) U.S. Virgin Islands

( ) Puerto Rico

( ) Guam

( ) Mexico

( ) Haiti

( ) Dominican Republic

( ) Cuba

( ) Other

( ) Prefer not to answer

( ) Don't know

Shortname / Alias: race_

#### ****What is your race or ethnicity? Select all that apply.*****

[ ] White, for example: German, Irish, English, Italian, Polish, French, etc.

[ ] Hispanic, Latino, or Spanish, for example: Mexican or Mexican American, Puerto Rican, Cuban, Salvadoran, Dominican, Colombian, etc.

[ ] Black or African American, for example: African American, Jamaican, Haitian, Nigerian, Ethiopian, Somali, etc.

[ ] Asian, for example: Chinese, Filipino, Asian Indian, Vietnamese, Korean, Japanese, etc.

[ ] American Indian or Alaska Native, for example: Navajo Nation, Blackfeet Tribe, Mayan, Aztec, Native Village of Barrow Inupiat Traditional Government, Tlingit, etc.

[ ] Middle Eastern or North African, for example: Lebanese, Iranian, Egyptian, Syrian, Moroccan, Algerian, etc.

[ ] Native Hawaiian or Other Pacific Islander, for example: Native Hawaiian, Samoan, Chamorro, Tongan, Fijian, Marshallese, etc.

[ ] Some other race, ethnicity, or origin:: _________________________________________________*

[ ] Prefer not to answer

[ ] Don't know

Shortname / Alias: sexual_id

#### ****How do you describe your current sexual identity?*****

( ) Gay/Homosexual

( ) Lesbian

( ) Heterosexual or Straight

( ) Bisexual

( ) Pansexual

( ) Queer

( ) Another sexual identity, please specify:: _________________________________________________*

( ) Prefer not to answer

( ) Don't know

Shortname / Alias: pronoun_

#### ****What are your pronouns?****

[ ] He/Him/His

[ ] She/Her/Hers

[ ] They/Them/Theirs

[ ] Something else, specify:: _________________________________________________*

[ ] Prefer not to answer

[ ] Don't know

## Demographics - PART 2

Shortname / Alias: hlevedu

#### ****What is the highest level of education you have completed?****

( ) Never attended school

( ) Less than high school

( ) Some high school

( ) High school diploma or GED

( ) Some college, Associate's Degree, or Technical Degree

( ) College graduate

( ) Post graduate or professional school

( ) Prefer not to answer

( ) Don't know

**Logic: Show/hide trigger exists.**

Shortname / Alias: curr_enroll

#### ****Are you currently enrolled in school?****

( ) No

( ) Yes, full time

( ) Yes, part time

( ) Prefer not to answer

( ) Don't know

**Logic: Hidden unless: #8 Question "Are you currently enrolled in school?" is one of the following answers ("Yes, full time","Yes, part time")**

Shortname / Alias: curr_enroll_type

#### ****Which type of school are you currently enrolled in?****

( ) Technical school/trade school

( ) Undergraduate (two-year or four-year program)

( ) Graduate, post graduate or professional school

( ) Other:: _________________________________________________*

( ) Prefer not to answer

( ) Don't know

## Demographics - PART 3

**Logic: Show/hide trigger exists.**

Shortname / Alias: curr_employ

#### ****Which best describes your current employment status?****

( ) Employed for wages full-time

( ) Employed for wages part-time

( ) Self-employed

( ) A homemaker

( ) Retired

( ) Not employed but searching for work

( ) Not employed and not actively seeking work

( ) Unable to work (disabled)

( ) Full-time student

( ) Prefer not to answer

( ) Don't know

**Logic: Hidden unless: #10 Question "Which best describes your current employment status?" is one of the following answers ("Employed for wages full-time","Employed for wages part-time","Self-employed","A homemaker")**

Shortname / Alias: curr_occupation

### ****What is your main occupation?****

_________________________________________________

**Logic: Hidden by default**

### ****ERROR: The household income that you entered is less than your total income.****

Validation: Min = 0 Max = 9999999 Must be currency

Shortname / Alias: total_income

### ****What was your total income last year (including income from all sources such as wages, salaries, Social Security or retirement benefits) before taxes?**** Please provide your best estimate.

_________________________________________________

**Logic: Show/hide trigger exists.**

Shortname / Alias: hh_income_rely

#### ****Do you rely on income from anyone else in your household or family?****

( ) Yes

( ) No

( ) Prefer not to answer

( ) Don't know

Validation: Min = 1 Max = 99 Must be numeric Whole numbers only Positive numbers only Max character count = 2

**Logic: Hidden unless: #13 Question "Do you rely on income from anyone else in your household or family?" is one of the following answers ("No")**

Shortname / Alias: income_support

### ****Including yourself, how many people depend on your income?****

_________________________________________________

Validation: Min = 0 Max = 9999999 Must be currency

**Logic: Show/hide trigger exists. Hidden unless: #13 Question "Do you rely on income from anyone else in your household or family?" is one of the following answers ("Yes")**

Shortname / Alias: hh_income

### ****What was your household income last year from all sources before taxes? That is, the total amount of money earned and shared by all people living in your household.**** Please provide your best estimate.

_________________________________________________

Validation: Min = 1 Max = 99 Must be numeric Whole numbers only Positive numbers only Max character count = 2

**Logic: Hidden unless: #15 Question "What was your household income last year from all sources before taxes? That is, the total amount of money earned and shared by all people living in your household. Please provide your best estimate."**

Shortname / Alias: hhincome_support

### ****Including yourself, how many people depend on this household income?****

_________________________________________________

Shortname / Alias: transport

#### ****Do you have access to a car, truck, or other form of transportation to get wherever you need to go?****

( ) Yes

( ) No

( ) Prefer not to answer

( ) Don't know

## Income Validation

## Demographics - PART 4

Shortname / Alias: state

#### ****What state do you live in?****

( ) Alabama

( ) Alaska

( ) Arizona

( ) Arkansas

( ) California

( ) Colorado

( ) Connecticut

( ) Delaware

( ) Florida

( ) Georgia

( ) Hawaii

( ) Idaho

( ) Illinois

( ) Indiana

( ) Iowa

( ) Kansas

( ) Kentucky

( ) Louisiana

( ) Maine

( ) Maryland

( ) Massachusetts

( ) Michigan

( ) Minnesota

( ) Mississippi

( ) Missouri

( ) Montana

( ) Nebraska

( ) Nevada

( ) New Hampshire

( ) New Jersey

( ) New Mexico

( ) New York

( ) North Carolina

( ) North Dakota

( ) Ohio

( ) Oklahoma

( ) Oregon

( ) Pennsylvania

( ) Rhode Island

( ) South Carolina

( ) South Dakota

( ) Tennessee

( ) Texas

( ) Utah

( ) Vermont

( ) Virginia

( ) Washington

( ) Washington, D.C.

( ) West Virginia

( ) Wisconsin

( ) Wyoming

Validation: %s format expected Using custom RegEx pattern

Shortname / Alias: zip_code

### ****What is your ZIP code?****

_________________________________________________

## Rural Zipcode Check

**Page entry logic:** This page will show when: #18 Question "**What state do you live in?**" is not one of the following answers ("Alabama","Arkansas","Delaware","Florida","Georgia","Kentucky","Louisiana","Maryland","Mississippi","Missouri","North Carolina","Oklahoma","South Carolina","Tennessee","Texas","Virginia","Washington, D.C.","West Virginia")

## State Flag

## Demographics - PART 5

Shortname / Alias: p12m_unstable_house

#### ****In the past 12 months (since [question('value'), id='4']), did you stay overnight with friends, relatives, or someone you didn’t know well because you didn’t have a regular, adequate, and safe place to stay at night?****

( ) Yes

( ) No

( ) Prefer not to answer

( ) Don't know

Shortname / Alias: p12m_homeless

#### ****In the past 12 months, were you ever homeless? That is, were you living on the street, in a shelter, in a hotel room, or in a car?****

( ) Yes

( ) No

( ) Prefer not to answer

( ) Don't know

Shortname / Alias: current_living

#### ****What is your living situation today?****

( ) I have a steady place to live

( ) I have a place to live today but am worried about losing it in the future

( ) I do not have a steady place to live (I am temporarily staying with others, in a hotel, in a shelter, living outside on the street, on a beach, in a car, abandoned building, bus or train station, or in a park)

( ) Prefer not to answer

( ) Don't know

**Logic: Show/hide trigger exists.**

Shortname / Alias: p12m_food_instability

#### ****In the past 12 months, did you or other adults in the household ever cut the size of your meals or skip meals because there wasn't enough money for food?****

( ) Yes

( ) No

( ) Prefer not to answer

( ) Don't know

**Logic: Hidden unless: #23 Question "In the past 12 months, did you or other adults in the household ever cut the size of your meals or skip meals because there wasn't enough money for food?" is one of the following answers ("Yes")**

Shortname / Alias: p12m_food_ins_freq

#### ****In the past 12 months, how often did you or other adults in the household ever cut the size of your meals or skip meals because there wasn't enough money for food?****

( ) Almost every month

( ) Some months but not every month

( ) Only 1 or 2 months

( ) Prefer not to answer

( ) Don't know

Shortname / Alias: p12m_jail

#### ****In the past 12 months, how many different times were you in jail, detention, or prison for longer than 24 hours?****

( ) 0

( ) 1

( ) 2

( ) 3

( ) 4

( ) 5+

( ) Prefer not to answer

( ) Don't know

## Health Care Access and Use

Shortname / Alias: health_ins_

#### ****What kind of health insurance or health care coverage do you currently have?**** Check all that apply.

[ ] I don't currently have any health insurance

[ ] A private health plan purchased through an employer or school

[ ] A private health plan purchased through an exchange (i.e. Obamacare)

[ ] Covered on parent's insurance policy

[ ] Medicaid or Medicare

[ ] Some other Medical Assistance program

[ ] TRICARE (CHAMPUS)

[ ] Veterans Administration coverage

[ ] Some other health care plan:: _________________________________________________*

[ ] Prefer not to answer

[ ] Don't know

**Logic: Show/hide trigger exists.**

Shortname / Alias: p12m_nurse

#### ****In the past 12 months (since [question('value'), id='4']), have you seen a doctor, nurse, or other health care provider about your own health?****

( ) Yes

( ) No

( ) Prefer not to answer

( ) Don't know

**Logic: Hidden unless: #27 Question "In the past 12 months (since [question('value'), id='4']), have you seen a doctor, nurse, or other health care provider about your own health?" is one of the following answers ("Yes")**

Shortname / Alias: provider_type_

#### ****What type of healthcare provider have you visited?**** Please check all that apply

[ ] Primary care provider

[ ] Urgent care clinic

[ ] Emergency room

[ ] Sexual health clinic

[ ] Mental health provider

[ ] Other:: _________________________________________________*

**Logic: Hidden unless: #27 Question "In the past 12 months (since [question('value'), id='4']), have you seen a doctor, nurse, or other health care provider about your own health?" is one of the following answers ("Yes")**

Shortname / Alias: visit_reason_

#### ****What was the reason for your visit(s)?**** Please check all that apply.

[ ] Physical exam/regular wellness visit

[ ] Sick visit

[ ] Sexual health/STI testing/HIV testing

[ ] Other:: _________________________________________________*

## Health Care Access and Use - PART 2

Shortname / Alias: reg_provider

#### ****Do you have a regular healthcare provider? That is, someone you see when you are sick or for routine physical exams.****

( ) Yes, I have a regular provider

( ) Yes, I have a regular clinic like an urgent care or CVS Minute Clinic

( ) No regular provider

( ) Prefer not to answer

( ) Don't know

Shortname / Alias: p12m_care_avoid

#### ****In the past 12 months (since [question('value'), id='4']), have you ever avoided visiting a healthcare provider because you were concerned about cost or your ability to pay?****

( ) Yes

( ) No

( ) Prefer not to answer

( ) Don't know

Shortname / Alias: told_provider_msm

#### ****Have you ever told a healthcare provider that you are attracted to or have sex with men?****

( ) Yes

( ) No

( ) Prefer not to answer

( ) Don't know

## HIV Testing History

**Logic: Show/hide trigger exists.**

Shortname / Alias: HIV_evertest

#### ****Have you ever been tested for HIV? An HIV test checks whether some has the virus that causes AIDS.****

( ) Yes

( ) No

( ) Prefer not to answer

( ) Don't know

**Logic: Hidden unless: #33 Question "Have you ever been tested for HIV? An HIV test checks whether some has the virus that causes AIDS. " is one of the following answers ("No")**

Shortname / Alias: reason_notest_

#### ****Which of these best describes the reason(s) you have not had an HIV test?**** Select all that apply.

[ ] I feel at low risk for HIV infection

[ ] Afraid of finding out that you had HIV

[ ] Haven't had the time

[ ] Haven't had the opportunity

[ ] Not sure where to get tested

[ ] Always know my partner's status, so never needed to get tested

[ ] No particular reason

[ ] Some other reason, please specify:: _________________________________________________*

[ ] Prefer not to answer

[ ] Don't know

**Logic: Hidden unless: #33 Question "Have you ever been tested for HIV? An HIV test checks whether some has the virus that causes AIDS. " is one of the following answers ("Yes")**

Shortname / Alias: p2yrs_num_tests

#### ****In the past 2 years (since [question('value'), id='5']), how many times have you been tested for HIV?**** Please provide your best estimate.

( ) 1

( ) 2

( ) 3

( ) 4

( ) 5

( ) 6

( ) 7

( ) 8

( ) 9

( ) 10+

( ) I did not test for HIV in the past 2 years

( ) Prefer not to answer

( ) Don't know

**Logic: Hidden unless: #33 Question "Have you ever been tested for HIV? An HIV test checks whether some has the virus that causes AIDS. " is one of the following answers ("Yes")**

Shortname / Alias: p12m_hivtest

#### ****Have you had an HIV test in the past 12 months (since [question('value'), id='4'])?****

( ) Yes

( ) No

( ) Prefer not to answer

( ) Don't know

**Page entry logic:** This page will show when: #33 Question "**Have you ever been tested for HIV? An HIV test checks whether some has the virus that causes AIDS.** " is one of the following answers ("Yes")

## HIV Testing - PART 2

Shortname / Alias: HIV_wheretest

#### ****When you most recently got tested, where did you get tested?****

( ) Private doctor's office

( ) HIV counseling and testing site

( ) Public health clinic/community health clinic

( ) Street outreach program/mobile unit

( ) Sexually transmitted disease clinic

( ) Hospital (inpatient)

( ) Correctional facility (jail or prison)

( ) Emergency room

( ) At home

( ) Other, please specify:: _________________________________________________*

( ) Prefer not to answer

( ) Don't know

Shortname / Alias: distance_hivtest

#### ****How far did you travel to obtain your most recent HIV test?****

( ) Less than 10 miles

( ) 10 - 20 miles

( ) More than 20 miles

( ) N/A (e.g., at-home test shipped to home)

( ) Prefer not to answer

( ) Don't know

Shortname / Alias: time_hivtest

#### ****How long did it take you to travel to obtain your most recent HIV test?****

( ) 15 minutes or less

( ) 16 - 30 minutes

( ) 31 - 60 minutes

( ) More than one hour

( ) N/A (e.g., at-home test shipped to home)

( ) Prefer not to answer

( ) Don't know

Shortname / Alias: result_test

#### ****What was the result of your most recent HIV test?****

( ) Negative

( ) Positive

( ) Never obtained results

( ) Indeterminate

( ) Prefer not to answer

( ) Don't know

**Page entry logic:** This page will show when: #40 Question "**What was the result of your most recent HIV test?**" is one of the following answers ("Positive")

## Positive HIV Test Email Action and Flag

**Page entry logic:** This page will show when: #33 Question "**Have you ever been tested for HIV? An HIV test checks whether some has the virus that causes AIDS.** " is one of the following answers ("Yes")

## HIV Testing - PART 3

**Logic: Show/hide trigger exists.**

Shortname / Alias: ever_rapid_test

#### ****FDA-approved rapid self-tests allow people to complete an HIV test anonymously at home and receive their results in as little as 20 minutes. Have you ever used a self-test to test for HIV?****

( ) Yes

( ) No

( ) Prefer not to answer

( ) Don't know

**Logic: Hidden unless: #41 Question "FDA-approved rapid self-tests allow people to complete an HIV test anonymously at home and receive their results in as little as 20 minutes. Have you ever used a self-test to test for HIV?" is one of the following answers ("Yes")**

Shortname / Alias: where_self_test

#### ****Where did you get the most recent HIV self-test that you used?****

( ) Pharmacy

( ) A sex partner

( ) A friend or family member

( ) Research study or government program

( ) Online

( ) Other, please specify:: _________________________________________________*

Shortname / Alias: HIV_testoften

#### ****Do you currently test for HIV regularly (after a given amount of time has passed)?****

( ) Yes

( ) No

( ) Prefer not to answer

( ) Don't know

Shortname / Alias: HIV_testfreq

#### ****About how often do you test for HIV? Please choose the option that best represents how often you test.****

( ) Every month

( ) Every 3 months

( ) Every 6 months

( ) Once a year

( ) Once every 2 years

( ) Other, please specify:: _________________________________________________*

## HIV Testing History - PART 4

Shortname / Alias: recom_test

#### ****As far as you know, what is the current recommendation for how often gay, bisexual, and other men who have sex with men should test for HIV?****

( ) Every month

( ) Every 3-6 months

( ) Once a year

( ) Less often than once a year

( ) Prefer not to answer

( ) Don't know

Shortname / Alias: HIV_tf_

#### ****Please read the statements below about HIV. Click the button to indicate if you think the statement is true or false.****

|  | **True** | **False** | **Don't know** |
| --- | --- | --- | --- |
| A person who is living with HIV can look healthy. | ( ) | ( ) | ( ) |
| If a person is living with HIV, they can show symptoms within a month of acquiring HIV. | ( ) | ( ) | ( ) |
| There is a vaccine that can stop you from getting HIV. | ( ) | ( ) | ( ) |
| Even if your partner has HIV, the risk for getting HIV is very low when deep kissing (tongue in partner's mouth). | ( ) | ( ) | ( ) |
| Nearly all HIV transmission comes from having lots of boyfriends or hook-ups. | ( ) | ( ) | ( ) |
| The risk for getting HIV is very low when having oral sex. | ( ) | ( ) | ( ) |
| A person is more likely to get HIV from receptive sex (bottom) than insertive sex (top). | ( ) | ( ) | ( ) |
| Showering or washing your genitals/private parts after having sex will make you less likely to get HIV. | ( ) | ( ) | ( ) |

## HIV Testing History - PART 5

Shortname / Alias: info_

#### ****Do you feel you have all the information you need about each of the following, or would you like to have more information?****

|  | **Have all the information you need** | **Would like to have more information** |
| --- | --- | --- |
| Where to get tested for HIV | ( ) | ( ) |
| How to bring up the topic of getting an HIV test with your partner | ( ) | ( ) |
| How often you should get tested for HIV | ( ) | ( ) |
| How to talk with a health care provider about HIV | ( ) | ( ) |
| How effective condoms are in preventing HIV | ( ) | ( ) |
| Pre-exposure prophylaxis (PrEP), medication taken to prevent HIV infection amount at risk HIV-negative people | ( ) | ( ) |
| Post-exposure prophylaxis (PEP), medication taken by HIV-negative people within 72 hours of being exposed to HIV to reduce the chance of becoming infected | ( ) | ( ) |
| Treatment as prevention (TasP), antiretroviral treatment (ART) taken by people living with HIV to decrease the risk of HIV transmission to those that are HIV-negative | ( ) | ( ) |
| When to begin treatment if living with HIV | ( ) | ( ) |
| How risky different sexual behaviors are in terms of HIV transmission | ( ) | ( ) |

## Perceived HIV Risk

Shortname / Alias: agdisag_

#### ****Please indicate how much you agree or disagree with the following statements.****

|  | **Strongly agree** | **Agree** | **Disagree** | **Strongly disagree** |
| --- | --- | --- | --- | --- |
| If one is destined to acquire HIV, there's nothing you can do about it. | ( ) | ( ) | ( ) | ( ) |
| Most of my friends think that getting HIV sooner or later is unavoidable. | ( ) | ( ) | ( ) | ( ) |
| Most of my friends believe they can do something to prevent HIV transmission. | ( ) | ( ) | ( ) | ( ) |
| Concerns about HIV make me anxious about having sex. | ( ) | ( ) | ( ) | ( ) |

## Perceived HIV Risk - PART 2

Shortname / Alias: HIV_chance

#### ****I think my chances of getting infected with HIV are:****

( ) Almost zero

( ) Small

( ) Moderate

( ) Large

( ) Very large

Shortname / Alias: HIV_concern

#### ****Getting an HIV infection is something I am:****

( ) Not concerned about

( ) A little concerned about

( ) Moderately concerned about

( ) Concerned about a lot

( ) Extremely concerned about

Shortname / Alias: HIV_rate_concern

#### ****I am concerned about high rates of HIV infection among people like me:****

( ) Agree

( ) Undecided

( ) Disagree

Shortname / Alias: HIV_life_hard

#### ****Becoming HIV infected would make my life harder.****

( ) Strongly disagree

( ) Disagree

( ) Neutral

( ) Agree

( ) Strongly agree

Shortname / Alias: sti_hivrisk

#### ****Having a sexually transmitted infection (e.g., gonorrhea, chlamydia, syphilis) can increase my risk for getting HIV.****

( ) Strongly disagree

( ) Disagree

( ) Neutral

( ) Agree

( ) Strongly agree

Shortname / Alias: change_prev_hiv

#### ****I would be willing to make a change in my daily life to prevent HIV.****

( ) Strongly disagree

( ) Disagree

( ) Neutral

( ) Agree

( ) Strongly agree

## HIV Stigma

Shortname / Alias: stigma_agdisag_

#### ****Please indicate how much you agree or disagree with the following statements.****

|  | **Strongly agree** | **Agree** | **Neither agree nor disagree** | **Disagree** | **Strongly disagree** | **Prefer not to answer** | **Don't know** |
| --- | --- | --- | --- | --- | --- | --- | --- |
| Most people in my area would discriminate against someone with HIV. | ( ) | ( ) | ( ) | ( ) | ( ) | ( ) | ( ) |
| Most people in my area would support the rights of a person with HIV to live and work wherever they wanted to. | ( ) | ( ) | ( ) | ( ) | ( ) | ( ) | ( ) |
| Most people in my area would not be friends with someone with HIV. | ( ) | ( ) | ( ) | ( ) | ( ) | ( ) | ( ) |
| Most people in my area would think that people who got HIV through sex or drug use have gotten what they deserve. | ( ) | ( ) | ( ) | ( ) | ( ) | ( ) | ( ) |

## Barrier to Care Scale (BACS)

Shortname / Alias: bacs_

#### ****Please indicate to what extent each of the following circumstances makes it difficult for you to receive the care, services, or opportunities you wish to obtain.****

|  | **No problem at all** | **Very slight problem** | **Somewhat of a problem** | **Major problem** |
| --- | --- | --- | --- | --- |
| Long distances to medical facilities and personnel. | ( ) | ( ) | ( ) | ( ) |
| Medical personnel (e.g. physicians, nurses) who decline to provide direct care to persons with HIV/AIDS. | ( ) | ( ) | ( ) | ( ) |
| The lack of health care professionals who are adequately trained and competent in AIDS care. | ( ) | ( ) | ( ) | ( ) |
| The lack of transportation to access the services I need. | ( ) | ( ) | ( ) | ( ) |
| The shortage of psychologists, social workers, and mental health counsellors who can help address mental health issues. | ( ) | ( ) | ( ) | ( ) |
| The lack of psychological support groups for persons with HIV/AIDS. | ( ) | ( ) | ( ) | ( ) |
| The level of knowledge about HIV/AIDS among residents in the community. | ( ) | ( ) | ( ) | ( ) |
| Community residents’ stigma against persons living with HIV/AIDS. | ( ) | ( ) | ( ) | ( ) |
| The lack of employment opportunities for people living with HIV/AIDS. | ( ) | ( ) | ( ) | ( ) |
| The lack of supportive and understanding work environments for people living with HIV/AIDS. | ( ) | ( ) | ( ) | ( ) |
| My personal financial resources. | ( ) | ( ) | ( ) | ( ) |
| Lack of adequate and affordable housing. | ( ) | ( ) | ( ) | ( ) |

## STI Testing History

### ****Now we're going to ask you about testing for sexually transmitted infections (STIs).****

**Logic: Show/hide trigger exists.**

Shortname / Alias: sti_evertest

#### ****Have you ever been tested for the sexually transmitted infections gonorrhea, chlamydia, or syphilis?****

( ) Yes

( ) No

( ) Prefer not to answer

( ) Don't know

**Logic: Hidden unless: #57 Question "Have you ever been tested for the sexually transmitted infections gonorrhea, chlamydia, or syphilis?" is one of the following answers ("Yes")**

Shortname / Alias: distance_stitest

#### ****How far did you travel to obtain your most recent STI test?****

( ) Less than 10 miles

( ) 10 - 20 miles

( ) More than 20 miles

( ) Prefer not to answer

( ) Don't know

**Logic: Hidden unless: #57 Question "Have you ever been tested for the sexually transmitted infections gonorrhea, chlamydia, or syphilis?" is one of the following answers ("Yes")**

Shortname / Alias: time_stitest

#### ****How long did it take you to travel to obtain your most recent STI test?****

( ) 15 minutes or less

( ) 16 - 30 minutes

( ) 31 - 60 minutes

( ) More than one hour

( ) Prefer not to answer

( ) Don't know

**Logic: Show/hide trigger exists. Hidden unless: #57 Question "Have you ever been tested for the sexually transmitted infections gonorrhea, chlamydia, or syphilis?" is one of the following answers ("Yes")**

Shortname / Alias: p12m_stitest

#### ****In the past 12 months (since [question('value'), id='4']), were you tested for the sexually transmitted infections gonorrhea, chlamydia, or syphilis?****

( ) Yes

( ) No

( ) Prefer not to answer

( ) Don't know

Validation: Min = 1 Max = 999 Must be numeric Whole numbers only Positive numbers only Max character count = 3 Min character count = 1

**Logic: Hidden unless: #60 Question "In the past 12 months (since [question('value'), id='4']), were you tested for the sexually transmitted infections gonorrhea, chlamydia, or syphilis?" is one of the following answers ("Yes")**

Shortname / Alias: p12m_totalstitest

### ****In the past 12 months, how many times have you been tested for the sexually transmitted infections gonorrhea, chlamydia, or syphilis?****

_________________________________________________

**Logic: Hidden unless: #60 Question "In the past 12 months (since [question('value'), id='4']), were you tested for the sexually transmitted infections gonorrhea, chlamydia, or syphilis?" is one of the following answers ("Yes")**

Shortname / Alias: sti_test_

#### ****In the past 12 months, when you were tested for the sexually transmitted infections gonorrhea, chlamydia, or syphilis, what samples did you provide for testing?**** Check all that apply.

[ ] I had my blood drawn

[ ] I gave a urine sample

[ ] I had my rectum (butt) swabbed

[ ] I had my throat swabbed

[ ] Prefer not to answer

[ ] Don't know

**Logic: Hidden unless: #60 Question "In the past 12 months (since [question('value'), id='4']), were you tested for the sexually transmitted infections gonorrhea, chlamydia, or syphilis?" is one of the following answers ("Yes")**

Shortname / Alias: pref_stitest_

#### ****When you were tested for the sexually transmitted infections gonorrhea, chlamydia, or syphilis in the past 12 months, how were you tested?**** Check all that apply.

[ ] I went to an STI clinic, healthcare center, or doctor's office where the provider collected the samples

[ ] I picked up the testing kit at an STI clinic, healthcare center, or doctor's office and I swabbed myself while at home or at another location

[ ] An STI clinic, healthcare center, or doctor's office arranged to have a testing kit mailed to me and I swabbed myself while at home or at another location

[ ] I ordered a testing kit online (such as from Amazon) and I swabbed myself at while at home or at another location

[ ] Prefer not to answer

[ ] Don't know

Shortname / Alias: sti_pref_test

#### ****Many STI clinics and healthcare centers provide in-person STI testing. Looking ahead, what is your preferred approach to getting tested for sexually transmitted infections?**** Choose one option to select your preferred location.

( ) Going to an STI clinic, healthcare center, or doctor’s office where the provider collects the sample

( ) Picking up the testing kit at an STI clinic, healthcare center, or doctor’s office and swabbing myself while at home or at another location

( ) Having an STI clinic, healthcare center, or doctor’s office mail me a testing kit and swabbing myself while at home or at another location

( ) Ordering a testing kit online (such as from Amazon) and swabbing myself while at home or at another location

( ) Something else:: _________________________________________________*

( ) Prefer not to answer

( ) Don't know

## Bacterial STI Diagnoses

**Logic: Show/hide trigger exists. Hidden unless: #57 Question "Have you ever been tested for the sexually transmitted infections gonorrhea, chlamydia, or syphilis?" is not one of the following answers ("No")**

Shortname / Alias: sti_diag_

#### ****Has a doctor, nurse, or other health care provider ever told you that you had any of the following?**** Check all that apply.

[ ] Gonorrhea

[ ] Chlamydia

[ ] Syphilis

[ ] None of the above

[ ] Prefer not to answer

[ ] Don't know

**Logic: Hidden unless: #65 Question "Has a doctor, nurse, or other health care provider ever told you that you had any of the following? Check all that apply." is one of the following answers ("Gonorrhea")**

Shortname / Alias: p12m_gon

#### ****Was your gonorrhea diagnosed in the past 12 months (since [question('value'), id='4'])?****

( ) Yes

( ) No

( ) Prefer not to answer

( ) Don't know

**Logic: Hidden unless: #65 Question "Has a doctor, nurse, or other health care provider ever told you that you had any of the following? Check all that apply." is one of the following answers ("Chlamydia")**

Shortname / Alias: p12m_chla

#### ****Was your chlamydia diagnosed in the past 12 months (since [question('value'), id='4'])?****

( ) Yes

( ) No

( ) Prefer not to answer

( ) Don't know

**Logic: Hidden unless: #65 Question "Has a doctor, nurse, or other health care provider ever told you that you had any of the following? Check all that apply." is one of the following answers ("Syphilis")**

Shortname / Alias: p12m_syph

#### ****Was your syphilis diagnosed in the past 12 months (since [question('value'), id='4'])?****

( ) Yes

( ) No

( ) Prefer not to answer

( ) Don't know

Shortname / Alias: mpox_vax

#### ****Have you ever received a vaccine to prevent mpox (previously known as monkeypox)?****

( ) Yes

( ) No

( ) Prefer not to answer

( ) Don't know

## Sexual Behavior

### ****Next, we're going to ask you some questions about having sex. Please remember your answers will be kept private. While we acknowledge that people may have distinct names or ways of referring to their sexual body parts, we will be using standard terms because we need to be sure we are collecting the same information from everyone who takes the survey. It's important for the study that your answers be as accurate as possible. You may skip any questions you are not comfortable answering. Please let us know which questions are not applicable to you. We need to ask you all of these questions, even if some may not apply to your situation.**** Let's get started!

Shortname / Alias: sexlife_satis

#### ****How satisfied are you with your current sex life?****

( ) Very satisfied

( ) Satisfied

( ) Neutral

( ) Dissatisfied

( ) Very dissatisfied

( ) Prefer not to answer

( ) Don't know

## Sexual Behavior - Cis Male

### ****Please be aware of the time period we are asking about before you answer the question.****

**Logic: Show/hide trigger exists.**

Shortname / Alias: p12msex_men_

#### ****In the past 12 months (since [question('value'), id='4']), what types of sex have you had with cisgender men (assigned male sex at birth and identifies as male)?****

[ ] Oral sex (mouth on the penis)

[ ] Anal sex (penis in the butt)

[ ] I have not had any type of sex with a cisgender man in the past 12 months

Validation: Min = 1 Max = 999 Must be numeric Whole numbers only Positive numbers only Max character count = 3

**Logic: Show/hide trigger exists. Hidden unless: #71 Question "In the past 12 months (since [question('value'), id='4']), what types of sex have you had with cisgender men (assigned male sex at birth and identifies as male)?" is one of the following answers ("Oral sex (mouth on the penis)","Anal sex (penis in the butt)")**

Shortname / Alias: p12m_male_partnum

### ****During the last 12 months, with how many cisgender male partner(s) did you have anal or oral sex?*****

_________________________________________________

**Page entry logic:** This page will show when: (#71 Question "**In the past 12 months (since [question('value'), id='4']), what types of sex have you had with cisgender men (assigned male sex at birth and identifies as male)?**" is one of the following answers ("Oral sex (mouth on the penis)","Anal sex (penis in the butt)") AND #72 Question "**During the last 12 months, with how many cisgender male partner(s) did you have anal or oral sex?**" is greater than or equal to "1")

## Additional Questions - Cis Male Partners

**Logic: Hidden by default**

### ****The number of cisgender male partners without condom use you reported is greater than the total number of cisgender male partners you reported in the past 12 months. Please correct this.****

Validation: Min = 0 Max = 999 Must be numeric Whole numbers only Positive numbers only Max character count = 3

**Logic: Hidden unless: #72 Question "During the last 12 months, with how many cisgender male partner(s) did you have anal or oral sex?"**

Shortname / Alias: p12m_m_analsex

### ****Of the [question('value'), id='116'] cisgender male partner(s) you had sex with in the last 12 months (since [question('value'), id='4']), how many did you have anal sex with?****

_________________________________________________

Shortname / Alias: cism_exchange

#### ****Were any of the cisgender male sex partners an exchange partner - this is, a partner that you have sex with in exchange for money, drugs, food, or something else of value?****

( ) Yes

( ) No

( ) Prefer not to answer

( ) Don't know

**Page entry logic:** This page will show when: (#71 Question "**In the past 12 months (since [question('value'), id='4']), what types of sex have you had with cisgender men (assigned male sex at birth and identifies as male)?**" is one of the following answers ("Oral sex (mouth on the penis)","Anal sex (penis in the butt)") AND #72 Question "**During the last 12 months, with how many cisgender male partner(s) did you have anal or oral sex?**" is greater than or equal to "1")

## Cis Male Validation

## Sexual Behavior - Cis Female

### ****Please be aware of the time period we are asking about before you answer the question.****

**Logic: Show/hide trigger exists.**

Shortname / Alias: p12msex_women_

#### ****In the past 12 months (since [question('value'), id='4']), what types of sex have you had with cisgender women (assigned female sex at birth and identifies as female)?****

[ ] Oral sex (mouth on the penis)

[ ] Anal sex (penis in the butt)

[ ] Vaginal sex (penis in the vagina)

[ ] I have not had any type of sex with a cisgender woman in the past 12 months

Validation: Min = 1 Max = 999 Must be numeric Whole numbers only Positive numbers only Max character count = 3

**Logic: Show/hide trigger exists. Hidden unless: #75 Question "In the past 12 months (since [question('value'), id='4']), what types of sex have you had with cisgender women (assigned female sex at birth and identifies as female)?" is one of the following answers ("Anal sex (penis in the butt)","Vaginal sex (penis in the vagina)")**

Shortname / Alias: p12m_female_partnum

### ****During the last 12 months, with how many cisgender female partner(s) did you have vaginal or anal sex?****

_________________________________________________

**Page entry logic:** This page will show when: (#75 Question "**In the past 12 months (since [question('value'), id='4']), what types of sex have you had with cisgender women (assigned female sex at birth and identifies as female)?**" is one of the following answers ("Anal sex (penis in the butt)","Vaginal sex (penis in the vagina)") AND #76 Question "**During the last 12 months, with how many cisgender female partner(s) did you have vaginal or anal sex?**" is greater than or equal to "1")

## Additional Questions - Cis Female Partners

**Logic: Hidden by default**

### ****The number of cisgender female partners without condom use you reported is greater than the total number of cisgender female partners you reported in the past 12 months. Please correct this.****

Validation: Min = 0 Max = 999 Must be numeric Whole numbers only Positive numbers only Max character count = 3

**Logic: Hidden unless: #76 Question "During the last 12 months, with how many cisgender female partner(s) did you have vaginal or anal sex?"**

Shortname / Alias: p12m_fm_analsex

### ****Of the [question('value'), id='119'] cisgender female partner(s) you had sex with in the last 12 months (since [question('value'), id='4']), how many did you have vaginal or anal sex with without using a condom?****

_________________________________________________

**Logic: Hidden unless: #76 Question "During the last 12 months, with how many cisgender female partner(s) did you have vaginal or anal sex?"**

Shortname / Alias: p12m_fm_anal_sex_nocondom

#### ****When you had sex with your cisgender female partner(s) in the last 12 months (since [question('value'), id='4']), did you ever have vaginal or anal sex without a condom?****

( ) Yes

( ) No

( ) Prefer not to answer

( ) Don't know

Shortname / Alias: cisf_exchange

#### ****Were any of these cisgender female sex partners an exchange partner - that is, a partner that you have sex with in exchange for money, drugs, food, or something else of value?****

( ) Yes

( ) No

( ) Prefer not to answer

( ) Don't know

**Page entry logic:** This page will show when: (#75 Question "**In the past 12 months (since [question('value'), id='4']), what types of sex have you had with cisgender women (assigned female sex at birth and identifies as female)?**" is one of the following answers ("Anal sex (penis in the butt)","Vaginal sex (penis in the vagina)") AND #76 Question "**During the last 12 months, with how many cisgender female partner(s) did you have vaginal or anal sex?**" is greater than or equal to "1")

## Cis Female Validation

## Sexual Behavior - Trans Female

### ****Please be aware of the time period we are asking about before you answer the question.****

**Logic: Show/hide trigger exists.**

Shortname / Alias: p12msex_twomen_

#### ****In the past 12 months (since [question('value'), id='4']), what types of sex have you had with transgender female or transfeminine people (assigned male sex at birth and identifies as transfeminine or female)?****

[ ] Oral sex (mouth on the penis)

[ ] Anal sex (penis in the butt)

[ ] Vaginal sex (penis in the vagina)

[ ] I have not had any type of sex with a transgender woman in the past 12 months

Validation: Min = 1 Max = 999 Must be numeric Whole numbers only Positive numbers only Max character count = 3

**Logic: Show/hide trigger exists. Hidden unless: #80 Question "In the past 12 months (since [question('value'), id='4']), what types of sex have you had with transgender female or transfeminine people (assigned male sex at birth and identifies as transfeminine or female)?" is one of the following answers ("Anal sex (penis in the butt)","Vaginal sex (penis in the vagina)")**

Shortname / Alias: p12m_tfemale_partnum

### ****During the last 12 months, with how many trans feminine individuals did you have vaginal or anal sex?****

_________________________________________________

**Page entry logic:** This page will show when: (#80 Question "**In the past 12 months (since [question('value'), id='4']), what types of sex have you had with transgender female or transfeminine people (assigned male sex at birth and identifies as transfeminine or female)?**" is one of the following answers ("Anal sex (penis in the butt)","Vaginal sex (penis in the vagina)") AND #81 Question "**During the last 12 months, with how many trans feminine individuals did you have vaginal or anal sex?**" is greater than or equal to "1")

## Additional Questions - Trans Female

**Logic: Hidden by default**

### ****The number of trans feminine partners without condom use you reported is greater than the total number of trans feminine partners you reported in the past 12 months. Please correct this.****

Validation: Min = 0 Max = 999 Must be numeric Whole numbers only Positive numbers only Max character count = 3

**Logic: Hidden unless: #81 Question "During the last 12 months, with how many trans feminine individuals did you have vaginal or anal sex?"**

Shortname / Alias: p12m_tfm_analsex

### ****Of the [question('value'), id='120'] trans feminine partner(s) you had sex with in the last 12 months (since [question('value'), id='4']), how many did you have vaginal or anal sex with without using a condom?****

_________________________________________________

**Logic: Hidden unless: #81 Question "During the last 12 months, with how many trans feminine individuals did you have vaginal or anal sex?"**

Shortname / Alias: p12m_tf_anal_sex_nocondom

#### ****When you had sex with your trans feminine partner(s) in the last 12 months (since [question('value'), id='4']), did you ever have vaginal or anal sex without a condom?****

( ) Yes

( ) No

( ) Prefer not to answer

( ) Don't know

Shortname / Alias: transf_exchange

#### ****Were any of these trans feminine individuals an exchange partner - that is, a partner that you have sex with in exchange for money, drugs, food, or something else of value?****

( ) Yes

( ) No

( ) Prefer not to answer

( ) Don't know

**Page entry logic:** This page will show when: (#80 Question "**In the past 12 months (since [question('value'), id='4']), what types of sex have you had with transgender female or transfeminine people (assigned male sex at birth and identifies as transfeminine or female)?**" is one of the following answers ("Anal sex (penis in the butt)","Vaginal sex (penis in the vagina)") AND #81 Question "**During the last 12 months, with how many trans feminine individuals did you have vaginal or anal sex?**" is greater than or equal to "1")

## Trans Female Validation

## Sexual Behavior - Trans Male

### ****Please be aware of the time period we are asking about before you answer the question.****

**Logic: Show/hide trigger exists.**

Shortname / Alias: p12msex_tmen_

#### ****In the past 12 months (since [question('value'), id='4']), what types of sex have you had with transgender male or transmasculine people (assigned female sex at birth and identifies as transmasculine or male)?****

[ ] Oral sex (mouth on the penis)

[ ] Anal sex (penis in the butt)

[ ] Vaginal sex (penis in the vagina)

[ ] I have not had any type of sex with a transgender man in the past 12 months

Validation: Min = 1 Max = 999 Must be numeric Whole numbers only Positive numbers only Max character count = 3

**Logic: Show/hide trigger exists. Hidden unless: #85 Question "In the past 12 months (since [question('value'), id='4']), what types of sex have you had with transgender male or transmasculine people (assigned female sex at birth and identifies as transmasculine or male)?" is one of the following answers ("Anal sex (penis in the butt)","Vaginal sex (penis in the vagina)")**

Shortname / Alias: p12m_tmale_partnum

### ****During the last 12 months, with how many trans masculine individuals did you have penetrative sex?****

_________________________________________________

**Page entry logic:** This page will show when: (#85 Question "**In the past 12 months (since [question('value'), id='4']), what types of sex have you had with transgender male or transmasculine people (assigned female sex at birth and identifies as transmasculine or male)?**" is one of the following answers ("Anal sex (penis in the butt)","Vaginal sex (penis in the vagina)") AND #86 Question "**During the last 12 months, with how many trans masculine individuals did you have penetrative sex?**" is greater than or equal to "1")

## Additional Questions - Trans Male

**Logic: Hidden by default**

### ****The number of trans masculine partners without condom use you reported is greater than the total number of trans masculine partners you reported in the past 12 months. Please correct this.****

Validation: Min = 0 Max = 999 Must be numeric Whole numbers only Positive numbers only Max character count = 3

**Logic: Hidden unless: #86 Question "During the last 12 months, with how many trans masculine individuals did you have penetrative sex?"**

Shortname / Alias: p12m_tm_analsex

### ****Of the [question('value'), id='121'] trans masculine partner(s) you had sex with in the last 12 months (since [question('value'), id='4']), how many did you have penetrative sex with without using a condom?****

_________________________________________________

**Logic: Hidden unless: #86 Question "During the last 12 months, with how many trans masculine individuals did you have penetrative sex?"**

Shortname / Alias: p12m_tm_anal_sex_nocondom

#### ****When you had sex with your trans masculine partner(s) in the last 12 months (since [question('value'), id='4']), did you ever have vaginal or anal sex without a condom?****

( ) Yes

( ) No

( ) Prefer not to answer

( ) Don't know

Shortname / Alias: transm_exchange

#### ****Were any of these trans masculine individuals an exchange partner - that is, a partner that you have sex with in exchange for money, drugs, food, or something else of value?****

( ) Yes

( ) No

( ) Prefer not to answer

( ) Don't know

**Page entry logic:** This page will show when: (#85 Question "**In the past 12 months (since [question('value'), id='4']), what types of sex have you had with transgender male or transmasculine people (assigned female sex at birth and identifies as transmasculine or male)?**" is one of the following answers ("Anal sex (penis in the butt)","Vaginal sex (penis in the vagina)") AND #86 Question "**During the last 12 months, with how many trans masculine individuals did you have penetrative sex?**" is greater than or equal to "1")

## Trans Male Validation

## Sexual Behavior - Non-Binary

### ****Please be aware of the time period we are asking about before you answer the question.****

**Logic: Show/hide trigger exists.**

Shortname / Alias: p12msex_nonbin_

#### ****In the past 12 months (since [question('value'), id='4']), what types of sex have you had with non-binary/gender non-conforming/genderqueer/two-spirit people?****

[ ] Oral sex (mouth on the penis)

[ ] Anal sex (penis in the butt)

[ ] Vaginal sex (penis in the vagina)

[ ] I have not had any type of sex with a non-binary/gender non-conforming/genderqueer/two-spirit person in the past 12 months

Validation: Min = 1 Max = 999 Must be numeric Whole numbers only Positive numbers only Max character count = 3

**Logic: Show/hide trigger exists. Hidden unless: #90 Question "In the past 12 months (since [question('value'), id='4']), what types of sex have you had with non-binary/gender non-conforming/genderqueer/two-spirit people?" is one of the following answers ("Anal sex (penis in the butt)","Vaginal sex (penis in the vagina)")**

Shortname / Alias: p12m_nonbin_partnum

### ****During the last 12 months, with how many non-binary/genderqueer/two-spirit individuals did you have penetrative sex?****

_________________________________________________

**Page entry logic:** This page will show when: (#90 Question "**In the past 12 months (since [question('value'), id='4']), what types of sex have you had with non-binary/gender non-conforming/genderqueer/two-spirit people?**" is one of the following answers ("Anal sex (penis in the butt)","Vaginal sex (penis in the vagina)") AND #91 Question "**During the last 12 months, with how many non-binary/genderqueer/two-spirit individuals did you have penetrative sex?**" is greater than or equal to "1")

## Additional Questions - Non-Binary

**Logic: Hidden by default**

### ****The number of non-binary/genderqueer/two-spirit partners without condom use you reported is greater than the total number of non-binary/genderqueer/two-spirit partners you reported in the past 12 months. Please correct this.****

Validation: Min = 0 Max = 999 Must be numeric Whole numbers only Positive numbers only Max character count = 3

**Logic: Hidden unless: #91 Question "During the last 12 months, with how many non-binary/genderqueer/two-spirit individuals did you have penetrative sex?"**

Shortname / Alias: p12m_nb_analsex

### ****Of the [question('value'), id='122'] non-binary/genderqueer/two-spirit partner(s) you had sex with in the last 12 months (since [question('value'), id='4']), how many did you have penetrative sex with without using a condom?****

_________________________________________________

**Logic: Hidden unless: #91 Question "During the last 12 months, with how many non-binary/genderqueer/two-spirit individuals did you have penetrative sex?"**

Shortname / Alias: p12m_nb_anal_sex_nocondom

#### ****When you had sex with your non-binary/genderqueer/two-spirit partner(s) in the last 12 months (since [question('value'), id='4']), did you ever have vaginal or anal sex without a condom?****

( ) Yes

( ) No

( ) Prefer not to answer

( ) Don't know

Shortname / Alias: nb_exchange

#### ****Were any of these non-binary/genderqueer/two-spirit individuals an exchange partner - that is, a partner that you have sex with in exchange for money, drugs, food, or something else of value?****

( ) Yes

( ) No

( ) Prefer not to answer

( ) Don't know

**Page entry logic:** This page will show when: (#90 Question "**In the past 12 months (since [question('value'), id='4']), what types of sex have you had with non-binary/gender non-conforming/genderqueer/two-spirit people?**" is one of the following answers ("Anal sex (penis in the butt)","Vaginal sex (penis in the vagina)") AND #91 Question "**During the last 12 months, with how many non-binary/genderqueer/two-spirit individuals did you have penetrative sex?**" is greater than or equal to "1")

## Non-Binary Validation

## Additional sex questions - Exchange

**Logic: Hidden unless: ((((#74 Question "Were any of the cisgender male sex partners an exchange partner - this is, a partner that you have sex with in exchange for money, drugs, food, or something else of value?" is one of the following answers ("Yes") OR #79 Question "Were any of these cisgender female sex partners an exchange partner - that is, a partner that you have sex with in exchange for money, drugs, food, or something else of value?" is one of the following answers ("Yes")) OR #84 Question "Were any of these trans feminine individuals an exchange partner - that is, a partner that you have sex with in exchange for money, drugs, food, or something else of value?" is one of the following answers ("Yes")) OR #89 Question "Were any of these trans masculine individuals an exchange partner - that is, a partner that you have sex with in exchange for money, drugs, food, or something else of value?" is one of the following answers ("Yes")) OR #94 Question "Were any of these non-binary/genderqueer/two-spirit individuals an exchange partner - that is, a partner that you have sex with in exchange for money, drugs, food, or something else of value?" is one of the following answers ("Yes"))**

Shortname / Alias: exchange_type

#### ****You said you exchanged sex for money, drugs, food, or something else of value. Do you receive goods/money, give goods/money or both?****

( ) I received goods and/or money

( ) I gave goods and/or money

( ) Both

( ) Prefer not to answer

( ) Don't know

## Partner calculating

**Page entry logic:** This page will show when: #72 Question "**During the last 12 months, with how many cisgender male partner(s) did you have anal or oral sex?**" is exactly equal to "1"

## 1 Cis Male Partner

Shortname / Alias: one_cism_main_part

#### ****Earlier you told us you have had one male partner in the past 12 months (since [question('value'), id='4']). Is/was this partner a main partner? A main partner is someone that you feel committed to above all others -- this is someone you might call your boyfriend, significant other, or life partner.****

( ) Yes

( ) No

( ) Prefer not to answer

( ) Don't know

Shortname / Alias: one_cism_ongoing

#### ****Next, think of whether you are currently in an active, ongoing sexual relationship with your male partner. By that, we mean that you generally have oral or anal sex at least once per month and you expect to continue doing so for some time. Is the relationship with this partner active and ongoing?****

( ) Yes

( ) No

( ) Prefer not to answer

( ) Don't know

**Logic: Hidden unless: (#97 Question "Next, think of whether you are currently in an active, ongoing sexual relationship with your male partner. By that, we mean that you generally have oral or anal sex at least once per month and you expect to continue doing so for some time. Is the relationship with this partner active and ongoing?" is one of the following answers ("No") AND #96 Question "Earlier you told us you have had one male partner in the past 12 months (since [question('value'), id='4']). Is/was this partner a main partner? A main partner is someone that you feel committed to above all others -- this is someone you might call your boyfriend, significant other, or life partner." is one of the following answers ("No"))**

Shortname / Alias: onepart_onetime

#### ****Was this a one-time partner?****

( ) Yes

( ) No

( ) Prefer not to answer

( ) Don't know

**Logic: Show/hide trigger exists. Hidden unless: ((#96 Question "Earlier you told us you have had one male partner in the past 12 months (since [question('value'), id='4']). Is/was this partner a main partner? A main partner is someone that you feel committed to above all others -- this is someone you might call your boyfriend, significant other, or life partner." is one of the following answers ("Yes") OR #97 Question "Next, think of whether you are currently in an active, ongoing sexual relationship with your male partner. By that, we mean that you generally have oral or anal sex at least once per month and you expect to continue doing so for some time. Is the relationship with this partner active and ongoing?" is one of the following answers ("Yes")) OR #98 Question "Was this a one-time partner?" is one of the following answers ("No"))**

Shortname / Alias: p12m_one_cism_freq

#### ****In the last 12 months, how often did you have anal sex with this partner? Please give us your best estimate.****

( ) No anal sex

( ) Less than once per month

( ) Once per month

( ) 2-3 times per month

( ) Once per week

( ) More than once per week

**Logic: Show/hide trigger exists. Hidden unless: #99 Question "In the last 12 months, how often did you have anal sex with this partner? Please give us your best estimate." is one of the following answers ("Less than once per month","Once per month","2-3 times per month","Once per week","More than once per week")**

Shortname / Alias: one_cism_sextype_

#### ****In the last 12 months, which of the following did you do with this partner?**** Check all that apply.

[ ] Receptive anal sex (you bottomed)

[ ] Insertive anal sex (you topped)

**Logic: Hidden unless: #100 Question "In the last 12 months, which of the following did you do with this partner? Check all that apply." is one of the following answers ("Receptive anal sex (you bottomed)")**

Shortname / Alias: one_cism_recept_cond

#### ****In the past 12 months, when you had receptive anal sex (you bottomed) with this partner, how often were you fully protected by a condom? This means you or your partner used a condom the entire time you had sex, and the condom did not break or fall off.****

( ) Never

( ) Rarely

( ) Sometimes

( ) Often

( ) Always

( ) Prefer not to answer

( ) Don't know

**Logic: Hidden unless: #100 Question "In the last 12 months, which of the following did you do with this partner? Check all that apply." is one of the following answers ("Insertive anal sex (you topped)")**

Shortname / Alias: one_cism_insert_cond

#### ****In the past 12 months, when you had insertive anal sex (you topped) with this partner, how often were you fully protected by a condom? This means you or your partner used a condom the entire time you had sex, and the condom did not break or fall off.****

( ) Never

( ) Rarely

( ) Sometimes

( ) Often

( ) Always

( ) Prefer not to answer

( ) Don't know

**Page entry logic:** This page will show when: #72 Question "**During the last 12 months, with how many cisgender male partner(s) did you have anal or oral sex?**" is greater than "1"

## >1 Cis Male Partner

**Logic: Hidden by default**

### ****The number of active and ongoing partners is greater than the total number of cisgender male partners.****

Validation: Min = 0 Max = 999 Must be numeric Whole numbers only Positive numbers only Max character count = 3 Min character count = 1

Shortname / Alias: mto_cism_active

### ****Earlier you told us you have had [question('value'), id='116'] cisgender male partners in the past 12 months (since [question('value'), id='4']). How many of your partners are active and ongoing? By that, we mean that you generally have oral or anal sex at least once per month and you expect to continue doing so for some time.****

_________________________________________________

**Logic: Show/hide trigger exists.**

Shortname / Alias: mto_cism_mainpart

#### ****Are/were any of these [question('value'), id='116'] partners a main partner? A main partner is someone that you feel committed to above all others - this is someone you might call your boyfriend, significant other, or spouse.****

( ) Yes

( ) No

( ) Prefer not to answer

( ) Don't know

**Logic: Hidden unless: #104 Question "Are/were any of these [question('value'), id='116'] partners a main partner? A main partner is someone that you feel committed to above all others - this is someone you might call your boyfriend, significant other, or spouse." is one of the following answers ("Yes")**

Shortname / Alias: mto_cism_caspart

#### ****Are/were any of these [question('value'), id='116'] partners a non-main (casual) partner?****

( ) Yes

( ) No

( ) Prefer not to answer

( ) Don't know

**Page entry logic:** This page will show when: #72 Question "**During the last 12 months, with how many cisgender male partner(s) did you have anal or oral sex?**" is greater than "1"

## Partner answer validation

**Page entry logic:** This page will show when: #104 Question "**Are/were any of these [question('value'), id='116'] partners a main partner? A main partner is someone that you feel committed to above all others - this is someone you might call your boyfriend, significant other, or spouse.**" is one of the following answers ("Yes")

## >1 Cis Male At Least 1 Main

### ****For the next few questions we are interested in hearing about your main partner(s) over the past 12 months (since [question('value'), id='4']).****

**Logic: Show/hide trigger exists.**

Shortname / Alias: p12m_main_anal

#### ****In the last 12 months, how often did you have anal sex with your main partner(s)?****

( ) No anal sex

( ) Less than once per month

( ) Once per month

( ) 2-3 times per month

( ) Once per week

( ) More than once per week

**Logic: Show/hide trigger exists. Hidden unless: #106 Question "In the last 12 months, how often did you have anal sex with your main partner(s)?" is one of the following answers ("Less than once per month","Once per month","2-3 times per month","Once per week","More than once per week")**

Shortname / Alias: main_cism_sextype_

#### ****In the last 12 months, which of the following did you do with your main partner(s)?**** Check all that apply.

[ ] Receptive anal sex (you bottomed with him)

[ ] Insertive anal sex (you topped with him)

**Logic: Hidden unless: #107 Question "In the last 12 months, which of the following did you do with your main partner(s)? Check all that apply." is one of the following answers ("Receptive anal sex (you bottomed with him)")**

Shortname / Alias: main_cism_recept_cond

#### ****In the past 12 months, when you had receptive anal sex (you bottomed) with your main partner(s), how often were you fully protected by a condom? This means you or your partner(s) used a condom the entire time you had sex, and the condom did not break or fall off.****

( ) Never

( ) Rarely

( ) Sometimes

( ) Often

( ) Always

( ) Prefer not to answer

( ) Don't know

**Logic: Hidden unless: #107 Question "In the last 12 months, which of the following did you do with your main partner(s)? Check all that apply." is one of the following answers ("Insertive anal sex (you topped with him)")**

Shortname / Alias: main_cism_insert_cond

#### ****In the past 12 months, when you had insertive anal sex (you topped) with your main partner(s), how often were you fully protected by a condom? This means you or your partner(s) used a condom the entire time you had sex, and the condom did not break or fall off.****

( ) Never

( ) Rarely

( ) Sometimes

( ) Often

( ) Always

( ) Prefer not to answer

( ) Don't know

**Page entry logic:** This page will show when: ((#105 Question "**Are/were any of these [question('value'), id='116'] partners a non-main (casual) partner?**" is not one of the following answers ("No","Prefer not to answer","Don't know") OR #104 Question "**Are/were any of these [question('value'), id='116'] partners a main partner? A main partner is someone that you feel committed to above all others - this is someone you might call your boyfriend, significant other, or spouse.**" is not one of the following answers ("Yes")) AND #72 Question "**During the last 12 months, with how many cisgender male partner(s) did you have anal or oral sex?**" is greater than "1")

## >1 Cis Male At Least 1 Casual

### ****For the next few questions we are interested in hearing about your non-main partners over the past 12 months (since [question('value'), id='4']).****

**Logic: Show/hide trigger exists.**

Shortname / Alias: p12m_cas_anal

#### ****In the last 12 months, how often did you have anal sex with your non-main partner(s)?****

( ) No anal sex

( ) Less than once per month

( ) Once per month

( ) 2-3 times per month

( ) Once per week

( ) More than once per week

( ) I did not have any non-main partners

**Logic: Show/hide trigger exists. Hidden unless: #110 Question "In the last 12 months, how often did you have anal sex with your non-main partner(s)?" is one of the following answers ("Less than once per month","Once per month","2-3 times per month","Once per week","More than once per week")**

Shortname / Alias: cas_cism_sextype_

#### ****In the last 12 months, which of the following did you do with your non-main partner(s)?**** Check all that apply.

[ ] Receptive anal sex (you bottomed with him)

[ ] Insertive anal sex (you topped with him)

**Logic: Hidden unless: #111 Question "In the last 12 months, which of the following did you do with your non-main partner(s)? Check all that apply." is one of the following answers ("Receptive anal sex (you bottomed with him)")**

Shortname / Alias: cas_cism_recept_cond

#### ****In the past 12 months, when you had receptive anal sex (you bottomed) with your non-main partner(s), how often were you fully protected by a condom? This means you or your partner used a condom the entire time you had sex, and the condom did not break or fall off.****

( ) Never

( ) Rarely

( ) Sometimes

( ) Often

( ) Always

( ) Prefer not to answer

( ) Don't know

**Logic: Hidden unless: #111 Question "In the last 12 months, which of the following did you do with your non-main partner(s)? Check all that apply." is one of the following answers ("Insertive anal sex (you topped with him)")**

Shortname / Alias: cas_cism_insert_cond

#### ****In the past 12 months, when you had insertive anal sex (you topped) with your non-main partner(s), how often were you fully protected by a condom? This means you or your partner(s) used a condom the entire time you had sex, and the condom did not break or fall off.****

( ) Never

( ) Rarely

( ) Sometimes

( ) Often

( ) Always

( ) Prefer not to answer

( ) Don't know

**Page entry logic:** This page will show when: (((((#71 Question "**In the past 12 months (since [question('value'), id='4']), what types of sex have you had with cisgender men (assigned male sex at birth and identifies as male)?**" is not one of the following answers ("Anal sex (penis in the butt)") OR #73 Question "**Of the [question('value'), id='116'] cisgender male partner(s) you had sex with in the last 12 months (since [question('value'), id='4']), how many did you have anal sex with?**" is exactly equal to "0") OR #99 Question "**In the last 12 months, how often did you have anal sex with this partner? Please give us your best estimate.**" is one of the following answers ("No anal sex")) OR ((((#106 Question "**In the last 12 months, how often did you have anal sex with your main partner(s)?**" is one of the following answers ("No anal sex") AND #110 Question "**In the last 12 months, how often did you have anal sex with your non-main partner(s)?**" is one of the following answers ("No anal sex")) AND #104 Question "**Are/were any of these [question('value'), id='116'] partners a main partner? A main partner is someone that you feel committed to above all others - this is someone you might call your boyfriend, significant other, or spouse.**" is one of the following answers ("Yes")) AND #105 Question "**Are/were any of these [question('value'), id='116'] partners a non-main (casual) partner?**" is one of the following answers ("Yes")) AND #72 Question "**During the last 12 months, with how many cisgender male partner(s) did you have anal or oral sex?**" is greater than "1")) OR ((((#106 Question "**In the last 12 months, how often did you have anal sex with your main partner(s)?**" is one of the following answers ("No anal sex") AND #110 Question "**In the last 12 months, how often did you have anal sex with your non-main partner(s)?**" ) AND #104 Question "**Are/were any of these [question('value'), id='116'] partners a main partner? A main partner is someone that you feel committed to above all others - this is someone you might call your boyfriend, significant other, or spouse.**" is one of the following answers ("Yes")) AND #105 Question "**Are/were any of these [question('value'), id='116'] partners a non-main (casual) partner?**" is not one of the following answers ("Yes")) AND #72 Question "**During the last 12 months, with how many cisgender male partner(s) did you have anal or oral sex?**" is greater than "1")) OR (((#110 Question "**In the last 12 months, how often did you have anal sex with your non-main partner(s)?**" is one of the following answers ("No anal sex") AND #106 Question "**In the last 12 months, how often did you have anal sex with your main partner(s)?**" ) AND #104 Question "**Are/were any of these [question('value'), id='116'] partners a main partner? A main partner is someone that you feel committed to above all others - this is someone you might call your boyfriend, significant other, or spouse.**" is not one of the following answers ("Yes")) AND #72 Question "**During the last 12 months, with how many cisgender male partner(s) did you have anal or oral sex?**" is greater than "1"))

## No Anal Sex Flag

**Page entry logic:** This page will show when: ((((#71 Question "**In the past 12 months (since [question('value'), id='4']), what types of sex have you had with cisgender men (assigned male sex at birth and identifies as male)?**" is one of the following answers ("Anal sex (penis in the butt)") OR #75 Question "**In the past 12 months (since [question('value'), id='4']), what types of sex have you had with cisgender women (assigned female sex at birth and identifies as female)?**" is one of the following answers ("Anal sex (penis in the butt)")) OR #80 Question "**In the past 12 months (since [question('value'), id='4']), what types of sex have you had with transgender female or transfeminine people (assigned male sex at birth and identifies as transfeminine or female)?**" is one of the following answers ("Anal sex (penis in the butt)")) OR #85 Question "**In the past 12 months (since [question('value'), id='4']), what types of sex have you had with transgender male or transmasculine people (assigned female sex at birth and identifies as transmasculine or male)?**" is one of the following answers ("Anal sex (penis in the butt)")) OR #90 Question "**In the past 12 months (since [question('value'), id='4']), what types of sex have you had with non-binary/gender non-conforming/genderqueer/two-spirit people?**" is one of the following answers ("Anal sex (penis in the butt)"))

## HIV-Positive Partners

**Logic: Show/hide trigger exists.**

Shortname / Alias: known_hiv_pos_analsex

#### ****In the past 12 months (since [question('value'), id='4']), have you had anal sex with any partners who you knew to be living with HIV?****

( ) Yes

( ) No

( ) Prefer not to answer

( ) Don't know

**Logic: Hidden unless: #114 Question "In the past 12 months (since [question('value'), id='4']), have you had anal sex with any partners who you knew to be living with HIV?" is one of the following answers ("Yes")**

Shortname / Alias: HIVpos_findout

#### ****When did you find out or when were you told that your sex partner was living with HIV?****

( ) Before having sex

( ) After having sex

**Logic: Hidden by default**

### ****The number of HIV-positive partners that you entered is greater than your total number of partners ([question('value'), id='420']) in the last 12 months.****

Validation: Min = 0 Max = 999 Must be numeric Whole numbers only Positive numbers only Max character count = 3 Min character count = 1

**Logic: Hidden unless: #114 Question "In the past 12 months (since [question('value'), id='4']), have you had anal sex with any partners who you knew to be living with HIV?" is one of the following answers ("Yes")**

Shortname / Alias: HIVpos_totpart

### ****In the past 12 months, how many of your partners were living with HIV?****

_________________________________________________

**Logic: Show/hide trigger exists. Hidden unless: #114 Question "In the past 12 months (since [question('value'), id='4']), have you had anal sex with any partners who you knew to be living with HIV?" is one of the following answers ("Yes")**

Shortname / Alias: hivpos_sextype_

#### ****In the last 12 months, which of the following did you do with partner(s) who were living with HIV?****Check all that apply.

[ ] Receptive anal sex (you bottomed)

[ ] Insertive anal sex (you topped)

**Logic: Hidden unless: #117 Question "In the last 12 months, which of the following did you do with partner(s) who were living with HIV? Check all that apply." is one of the following answers ("Receptive anal sex (you bottomed)")**

Shortname / Alias: hivpos_recept_cond

#### ****In the past 12 months, when you had receptive anal sex (you bottomed) with a partner who was living with HIV, how often were you fully protected by a condom? This means you or your partner used a condom the entire time you had sex, and the condom did not break or fall off.****

( ) Never

( ) Rarely

( ) Sometimes

( ) Often

( ) Always

( ) Prefer not to answer

( ) Don't know

**Logic: Hidden unless: #117 Question "In the last 12 months, which of the following did you do with partner(s) who were living with HIV? Check all that apply." is one of the following answers ("Insertive anal sex (you topped)")**

Shortname / Alias: hivpos_insert_cond

#### ****In the past 12 months, when you had insertive anal sex (you topped) with a partner who was living with HIV, how often were you fully protected by a condom? This means you or your partner used a condom the entire time you had sex, and the condom did not break or fall off.****

( ) Never

( ) Rarely

( ) Sometimes

( ) Often

( ) Always

( ) Prefer not to answer

( ) Don't know

**Logic: Show/hide trigger exists. Hidden unless: #114 Question "In the past 12 months (since [question('value'), id='4']), have you had anal sex with any partners who you knew to be living with HIV?" is one of the following answers ("Yes")**

Shortname / Alias: hivpart_untransmit

#### ****In the past 12 months, did any of your partner(s) living with HIV tell you that they were untransmittable or had an undetectable viral load (i.e., U=U)?****

( ) Yes

( ) No

( ) Prefer not to answer

( ) Don't know

**Logic: Hidden unless: #120 Question "In the past 12 months, did any of your partner(s) living with HIV tell you that they were untransmittable or had an undetectable viral load (i.e., U=U)?" is one of the following answers ("Yes")**

Shortname / Alias: all_hiv_part_untransmit

#### ****In the past 12 months, did all of your partner(s) living with HIV tell you that they were untransmittable or had an undetectable viral load (i.e., U=U)?****

( ) Yes

( ) No

( ) Prefer not to answer

( ) Don't know

**Page entry logic:** This page will show when: #114 Question "**In the past 12 months (since [question('value'), id='4']), have you had anal sex with any partners who you knew to be living with HIV?**" is one of the following answers ("Yes")

## HIV Partner Checking

**Page entry logic:** This page will show when: ((((#71 Question "**In the past 12 months (since [question('value'), id='4']), what types of sex have you had with cisgender men (assigned male sex at birth and identifies as male)?**" is not exactly equal to ("I have not had any type of sex with a cisgender man in the past 12 months") OR #75 Question "**In the past 12 months (since [question('value'), id='4']), what types of sex have you had with cisgender women (assigned female sex at birth and identifies as female)?**" is not exactly equal to ("I have not had any type of sex with a cisgender woman in the past 12 months")) OR #80 Question "**In the past 12 months (since [question('value'), id='4']), what types of sex have you had with transgender female or transfeminine people (assigned male sex at birth and identifies as transfeminine or female)?**" is not exactly equal to ("I have not had any type of sex with a transgender woman in the past 12 months")) OR #85 Question "**In the past 12 months (since [question('value'), id='4']), what types of sex have you had with transgender male or transmasculine people (assigned female sex at birth and identifies as transmasculine or male)?**" is not exactly equal to ("I have not had any type of sex with a transgender man in the past 12 months")) OR #90 Question "**In the past 12 months (since [question('value'), id='4']), what types of sex have you had with non-binary/gender non-conforming/genderqueer/two-spirit people?**" is not exactly equal to ("I have not had any type of sex with a non-binary/gender non-conforming/genderqueer/two-spirit person in the past 12 months"))

## Sexual Behavior: Unknown HIV Serostatus

**Logic: Show/hide trigger exists.**

Shortname / Alias: unknown_hiv_analsex

#### ****In the past 12 months (since [question('value'), id='4']), did you have anal sex WITHOUT a condom with a partner whose HIV status you did not know?****

( ) Yes

( ) No

( ) Prefer not to answer

( ) Don't know

**Logic: Show/hide trigger exists. Hidden unless: #122 Question "In the past 12 months (since [question('value'), id='4']), did you have anal sex WITHOUT a condom with a partner whose HIV status you did not know?" is one of the following answers ("Yes")**

Shortname / Alias: hivunk_sextype_

#### ****In the last 12 months, which of the following did you do with partner(s) whose HIV status you did not know?**** Check all that apply.

[ ] Receptive anal sex (you bottomed)

[ ] Insertive anal sex (you topped)

**Logic: Hidden unless: #123 Question "In the last 12 months, which of the following did you do with partner(s) whose HIV status you did not know? Check all that apply." is one of the following answers ("Receptive anal sex (you bottomed)")**

Shortname / Alias: hivunk_recept_cond

#### ****In the past 12 months, when you had receptive anal sex (you bottomed) with a partner whose HIV status you did not know, how often were you fully protected by a condom? This means you or your partner used a condom the entire time you had sex, and the condom did not break or fall off.****

( ) Never

( ) Rarely

( ) Sometimes

( ) Often

( ) Always

( ) Prefer not to answer

( ) Don't know

**Logic: Hidden unless: #123 Question "In the last 12 months, which of the following did you do with partner(s) whose HIV status you did not know? Check all that apply." is one of the following answers ("Insertive anal sex (you topped)")**

Shortname / Alias: hivunk_insert_cond

#### ****In the past 12 months, when you had insertive anal sex (you topped) with a partner whose HIV status you did not know, how often were you fully protected by a condom? This means you or your partner used a condom the entire time you had sex, and the condom did not break or fall off.****

( ) Never

( ) Rarely

( ) Sometimes

( ) Often

( ) Always

( ) Prefer not to answer

( ) Don't know

Shortname / Alias: alc_intercourse

#### ****In the past 12 months, how often did you drink alcohol or use drugs before you had sexual intercourse?****

( ) Never

( ) Rarely

( ) Sometimes

( ) Often

( ) Always

## GIF4

###

## PrEP Module

### ****Pre-exposure prophylaxis (PrEP) is taking an antiretroviral medicine, either as a pill (also known as Truvada or Descovy) or as an injection (also known as Apretude), to reduce a person's chance of getting HIV.****

**Logic: Show/hide trigger exists.**

Shortname / Alias: heard_prep

#### ****Before today, had you ever heard of PrEP?****

( ) Yes

( ) No

( ) Prefer not to answer

( ) Don't know

Shortname / Alias: prepstart_free_

#### ****If PrEP was available from your local doctor and you could access it for free, would you start PrEP in the next month?**** Check all that apply.

[ ] Yes, oral PrEP

[ ] Yes, injectable PrEP

[ ] No

[ ] Prefer not to answer

[ ] Don't know

**Logic: Show/hide trigger exists. Hidden unless: #127 Question "Before today, had you ever heard of PrEP?" is one of the following answers ("Yes")**

Shortname / Alias: takenprep_

#### ****Have you ever taken PrEP?**** Check all that apply.

[ ] Yes, oral PrEP

[ ] Yes, injectable PrEP

[ ] No

[ ] Prefer not to answer

[ ] Don't know

**Logic: Show/hide trigger exists. Hidden unless: #129 Question "Have you ever taken PrEP? Check all that apply." is one of the following answers ("Yes, oral PrEP","Yes, injectable PrEP")**

Shortname / Alias: current_prep

#### ****Are you currently taking PrEP?****

( ) Yes

( ) No

( ) Prefer not to answer

( ) Don't know

**Logic: Hidden unless: #130 Question "Are you currently taking PrEP?" is one of the following answers ("Yes")**

Shortname / Alias: prescript_curr_prep

#### ****Which PrEP prescription medication are you currently taking?****

( ) Truvada (name brand or generic)

( ) Descovy

( ) Apretude

( ) Prefer not to answer

( ) Don't know

**Logic: Show/hide trigger exists. Hidden unless: (#129 Question "Have you ever taken PrEP? Check all that apply." is one of the following answers ("Yes, oral PrEP","Yes, injectable PrEP") AND #130 Question "Are you currently taking PrEP?" is one of the following answers ("No","Prefer not to answer","Don't know"))**

Shortname / Alias: p12m_prep

#### ****In the past 12 months (since [question('value'), id='4']), have you taken PrEP?****

( ) Yes

( ) No

( ) Prefer not to answer

( ) Don't know

**Logic: Hidden unless: #132 Question "In the past 12 months (since [question('value'), id='4']), have you taken PrEP?" is one of the following answers ("Yes")**

Shortname / Alias: p12m_prep_type_

#### ****In the past 12 months, which PrEP prescription medications did you take?**** Check all that apply.

[ ] Truvada (name brand or generic)

[ ] Descovy

[ ] Apretude

[ ] Prefer not to answer

[ ] Don't know

**Logic: Hidden unless: (#129 Question "Have you ever taken PrEP? Check all that apply." is one of the following answers ("Yes, oral PrEP","Yes, injectable PrEP") AND #130 Question "Are you currently taking PrEP?" is one of the following answers ("No","Prefer not to answer","Don't know"))**

Shortname / Alias: prep_stop_reas_

#### ****Which of the following describes the reason(s) why you stopped using PrEP the last time you were on it?**** Check all that apply.

[ ] The cost was too high

[ ] I lost my job and/or insurance

[ ] I experienced and/or was concerned about side-effects

[ ] I could not remember to take the pill every day

[ ] I started a monogamous relationship with an HIV-negative partner

[ ] I stopped being sexually active

[ ] I prefer to use other methods to protect myself from HIV

[ ] I was worried that people will think that I have HIV when they see me taking the pill

[ ] I was worried that people will know that I have sex with men or transgender people

[ ] I was worried people will think I am very sexually active because I am on PrEP

[ ] I was worried about my privacy with someone that I live with

[ ] I was worried about my privacy on my parent’s health insurance plan

[ ] I was unable or did not want to participate in the recommended sexually transmitted infection (gonorrhea, chlamydia, and syphilis) testing during clinic visits for PrEP care

[ ] Another reason, please specify:: _________________________________________________*

[ ] Prefer not to answer

[ ] Don't know

**Page entry logic:** This page will show when: #130 Question "**Are you currently taking PrEP?**" is one of the following answers ("Yes")

## Current PrEP Flag

## PrEP Opinion

### ****Please indicate how much you agree or disagree with the following statements.****

Shortname / Alias: prep_opinion_gay

#### ****Taking PrEP is a sign of being gay.****

( ) Disagree

( ) Somewhat disagree

( ) Neutral

( ) Somewhat agree

( ) Agree

( ) Don't know

Shortname / Alias: prep_opinion_top

#### ****I do not need PrEP if I am a "top".****

( ) Disagree

( ) Somewhat disagree

( ) Neutral

( ) Somewhat agree

( ) Agree

( ) Don't know

Shortname / Alias: prep_opinion_partners

#### ****I would feel comfortable telling my sexual partners if I take PrEP.****

( ) Disagree

( ) Somewhat disagree

( ) Neutral

( ) Somewhat agree

( ) Agree

( ) Don't know

Shortname / Alias: prep_opinion_providers

#### ****I would feel comfortable telling my medical providers if I take PrEP.****

( ) Disagree

( ) Somewhat disagree

( ) Neutral

( ) Somewhat agree

( ) Agree

( ) Don't know

Shortname / Alias: prep_opinion_friends

#### ****I would feel comfortable telling my friends if I used PrEP.****

( ) Disagree

( ) Somewhat disagree

( ) Neutral

( ) Somewhat agree

( ) Agree

( ) Don't know

## PrEP Adherence Self-Efficacy Scale

### ****We are going to ask you about situations that could occur while taking PrEP. For the following questions we will ask you to tell us how confident you are that you could to do the following things.****

### ****Use this response scale ranging from 0 ("could not do at all") to 10 ("certainly could do").****

Shortname / Alias: prep_scale_

### ****How confident are you that you could:****

Validation: Min = 0 Max = 10

Shortname / Alias: prep_scale_1

#### Stick to taking PrEP even when side effects begin to interfere with daily activities? Common side effects can include headache, nausea, stomach pain, and fatigue.

0 ________________________[__]_____________________________ 10

Validation: Min = 0 Max = 10

Shortname / Alias: prep_scale_2

#### Integrate taking PrEP into your daily routine?

0 ________________________[__]_____________________________ 10

Validation: Min = 0 Max = 10

Shortname / Alias: prep_scale_3

#### Integrate taking PrEP into your daily routine even if it means taking medications or doing other things in front of people who don't know you are taking PrEP?

0 ________________________[__]_____________________________ 10

Validation: Min = 0 Max = 10

Shortname / Alias: prep_scale_4

#### Stick to your PrEP schedule even when your daily routine is disrupted?

0 ________________________[__]_____________________________ 10

Validation: Min = 0 Max = 10

Shortname / Alias: prep_scale_5

#### Stick to your PrEP schedule when you aren't feeling well?

0 ________________________[__]_____________________________ 10

Validation: Min = 0 Max = 10

Shortname / Alias: prep_scale_6

#### Continue taking PrEP even if it means doing so interferes with your daily activities?

0 ________________________[__]_____________________________ 10

Validation: Min = 0 Max = 10

Shortname / Alias: prep_scale_7

#### Continue taking PrEP even when you are feeling discouraged about your sexual health?

0 ________________________[__]_____________________________ 10

Validation: Min = 0 Max = 10

Shortname / Alias: prep_scale_8

#### Continue taking PrEP even when getting to your clinic appointments is a major hassle?

0 ________________________[__]_____________________________ 10

Validation: Min = 0 Max = 10

Shortname / Alias: prep_scale_9

#### Continue taking PrEP even if people close to you tell you that they don't think it is doing any good?

0 ________________________[__]_____________________________ 10

## PrEP Agree/Disagree

Shortname / Alias: prep_agdisag_

#### ****Please indicate how much you agree or disagree with the following statements.****

|  | **Strongly agree** | **Agree** | **Neutral** | **Disagree** | **Strongly disagree** |
| --- | --- | --- | --- | --- | --- |
| If I use PrEP people will think less of me. | ( ) | ( ) | ( ) | ( ) | ( ) |
| I would feel ashamed to take PrEP pills in front of others. | ( ) | ( ) | ( ) | ( ) | ( ) |
| Someone taking PrEP should keep their pills hidden. | ( ) | ( ) | ( ) | ( ) | ( ) |
| People experience negative judgment because they take PrEP. | ( ) | ( ) | ( ) | ( ) | ( ) |
| I would have sex with someone who is taking PrEP. | ( ) | ( ) | ( ) | ( ) | ( ) |
| Someone taking PrEP would be seen by others as slutty. | ( ) | ( ) | ( ) | ( ) | ( ) |
| People taking PrEP receive praise for being responsible. | ( ) | ( ) | ( ) | ( ) | ( ) |
| My friends would be supportive of me taking PrEP. | ( ) | ( ) | ( ) | ( ) | ( ) |
| Someone taking PrEP would be treated unfairly by their doctors. | ( ) | ( ) | ( ) | ( ) | ( ) |
| People experience problems when they tell their sex partner(s) they are taking PrEP. | ( ) | ( ) | ( ) | ( ) | ( ) |
| I would feel proud to take PrEP every day. | ( ) | ( ) | ( ) | ( ) | ( ) |
| People taking PrEP experience verbal harassment. | ( ) | ( ) | ( ) | ( ) | ( ) |
| People on PrEP are taking care of their health. | ( ) | ( ) | ( ) | ( ) | ( ) |
| My family would be supportive of me taking PrEP. | ( ) | ( ) | ( ) | ( ) | ( ) |

## PrEP Importance Scale

### ****In the next few questions, we’re going to ask about how important some activities are to you and how confident you are that you can do them. Please use the scale provided to answer each question to the best of your ability.****

Validation: Min = 0 Max = 10

Shortname / Alias: importance_hivannualtest

#### ****On a scale of 0 to 10, with 0 being not at all important and 10 being very important, how**** important ****is it for you to test for HIV at least once per year?****

0 ________________________[__]_____________________________ 10

Validation: Min = 0 Max = 10

Shortname / Alias: confidence_hivannualtest

#### ****On a scale of 0 to 10, with 0 being not at all confident and 10 being very confident, how**** confident ****are you to test for HIV at least once per year?****

0 ________________________[__]_____________________________ 10

Validation: Min = 0 Max = 10

Shortname / Alias: importance_stiannualtest

#### ****On a scale of 0 to 10, with 0 being not at all important and 10 being very important, how**** important ****is it for you to test for STIs at least once per year?****

0 ________________________[__]_____________________________ 10

Validation: Min = 0 Max = 10

Shortname / Alias: confidence_stiannualtest

#### ****On a scale of 0 to 10, with 0 being not at all confident and 10 being very confident, how**** confident ****are you to test for STIs at least once per year?****

0 ________________________[__]_____________________________ 10

Validation: Min = 0 Max = 10

Shortname / Alias: importance_startprep

#### ****On a scale of 0 to 10, with 0 being not at all important and 10 being very important, how**** important ****is it for you to start taking PrEP to prevent HIV?****

0 ________________________[__]_____________________________ 10

Validation: Min = 0 Max = 10

Shortname / Alias: confidence_startprep

#### ****On a scale of 0 to 10, with 0 being not at all confident and 10 being very confident, how**** confident ****are you to start taking PrEP to prevent HIV?****

0 ________________________[__]_____________________________ 10

## DAST-10

### ****In the questions that follow we are going to ask about your drug use. This refers to any nonmedical drugs that you take or any prescriptions that you take in excess of the directions.****

**Logic: Show/hide trigger exists.**

Shortname / Alias: druguse_nonmedical

#### ****Have you used drugs other than those required for medical reasons?****

( ) Yes

( ) No

( ) Prefer not to answer

( ) Don't know

**Logic: Hidden unless: #148 Question "Have you used drugs other than those required for medical reasons?" is one of the following answers ("Yes")**

Shortname / Alias: druguse_mto

#### ****Do you use more than one drug at a time?****

( ) Yes

( ) No

( ) Prefer not to answer

( ) Don't know

**Logic: Hidden unless: #148 Question "Have you used drugs other than those required for medical reasons?" is one of the following answers ("Yes")**

Shortname / Alias: druguse_stop

#### ****Are you always able to stop using drugs when you want to?****

( ) Yes

( ) No

( ) Prefer not to answer

( ) Don't know

**Logic: Hidden unless: #148 Question "Have you used drugs other than those required for medical reasons?" is one of the following answers ("Yes")**

Shortname / Alias: druguse_blackout

#### ****Have you had "blackouts" or "flashbacks" as a result of drug use?****

( ) Yes

( ) No

( ) Prefer not to answer

( ) Don't know

**Logic: Hidden unless: #148 Question "Have you used drugs other than those required for medical reasons?" is one of the following answers ("Yes")**

Shortname / Alias: druguse_guilt

#### ****Do you ever feel bad or guilty about your drug use?****

( ) Yes

( ) No

( ) Prefer not to answer

( ) Don't know

**Logic: Hidden unless: #148 Question "Have you used drugs other than those required for medical reasons?" is one of the following answers ("Yes")**

Shortname / Alias: druguse_complain

#### ****Does your spouse (or parents) ever complain about your involvement with drugs?****

( ) Yes

( ) No

( ) Prefer not to answer

( ) Don't know

**Logic: Hidden unless: #148 Question "Have you used drugs other than those required for medical reasons?" is one of the following answers ("Yes")**

Shortname / Alias: druguse_neglect

#### ****Have you neglected your family because of your use of drugs?****

( ) Yes

( ) No

( ) Prefer not to answer

( ) Don't know

**Logic: Hidden unless: #148 Question "Have you used drugs other than those required for medical reasons?" is one of the following answers ("Yes")**

Shortname / Alias: druguse_illegal

#### ****Have you engaged in illegal activities in order to obtain drugs?****

( ) Yes

( ) No

( ) Prefer not to answer

( ) Don't know

**Logic: Hidden unless: #148 Question "Have you used drugs other than those required for medical reasons?" is one of the following answers ("Yes")**

Shortname / Alias: druguse_withdrawal

#### ****Have you ever experienced withdrawal symptoms (felt sick) when you stopped taking drugs?****

( ) Yes

( ) No

( ) Prefer not to answer

( ) Don't know

**Logic: Hidden unless: #148 Question "Have you used drugs other than those required for medical reasons?" is one of the following answers ("Yes")**

Shortname / Alias: druguse_medicalprob

#### ****Have you had medical problems as a result of your drug use (e.g., memory loss, hepatitis, convulsions, bleeding, etc.)?****

( ) Yes

( ) No

( ) Prefer not to answer

( ) Don't know

**Page entry logic:** This page will show when: #148 Question "**Have you used drugs other than those required for medical reasons?**" is one of the following answers ("Yes")

## Substance Use

### ****Now we’re going to ask some questions about your substance use history. Please remember that your answers will be kept private. You may skip any questions you are not comfortable answering. Please let us know which questions are not applicable to you. We need to ask you all of these questions, even if some may not apply to your situation.****

**Logic: Show/hide trigger exists.**

Shortname / Alias: drug_

#### ****In the past 12 months (since [question('value'), id='4']), which drugs that were not prescribed to you did you use? (Select all that you have taken in the past 12 months)****

[ ] Cannabis (marijuana, pot, grass, hash, etc.)

[ ] Cocaine (coke, crack, etc.)

[ ] Prescription stimulants just for the feeling, more than prescribed, or that were not prescribed for you (Ritalin, Adderall, diet pills, etc.)

[ ] Methamphetamine (meth, crystal, speed, ecstasy, molly, etc.)

[ ] Inhalants (nitrous, glue, paint thinner, poppers, whippets, etc.)

[ ] Sedatives just for the feeling, more than prescribed, or that were not prescribed for you (sleeping pills, Valium, Xanax, tranquilizers, benzos, etc.)

[ ] Hallucinogens (LSD, acid, mushrooms, PCP, Special K, ecstasy, etc.)

[ ] Street opioids (heroin, opium, etc.)

[ ] Prescription opioids just for the feeling, more than prescribed, or that were not prescribed for you (Fentanyl, Oxycodone, OxyContin, Percocet, Vicodin, methadone, Buprenorphine, etc.)

[ ] Any other drugs to get high; please specify:: _________________________________________________*

[ ] Prefer not to answer

[ ] Don't know

[ ] None of the above

## Substance Follow-Up Questions

**Logic: Hidden unless: #158 Question "In the past 12 months (since [question('value'), id='4']), which drugs that were not prescribed to you did you use? (Select all that you have taken in the past 12 months)" is one of the following answers ("Cannabis (marijuana, pot, grass, hash, etc.)","Cocaine (coke, crack, etc.)","Prescription stimulants just for the feeling, more than prescribed, or that were not prescribed for you (Ritalin, Adderall, diet pills, etc.)","Methamphetamine (meth, crystal, speed, ecstasy, molly, etc.)","Inhalants (nitrous, glue, paint thinner, poppers, whippets, etc.)","Sedatives just for the feeling, more than prescribed, or that were not prescribed for you (sleeping pills, Valium, Xanax, tranquilizers, benzos, etc.)","Hallucinogens (LSD, acid, mushrooms, PCP, Special K, ecstasy, etc.)","Street opioids (heroin, opium, etc.)","Prescription opioids just for the feeling, more than prescribed, or that were not prescribed for you (Fentanyl, Oxycodone, OxyContin, Percocet, Vicodin, methadone, Buprenorphine, etc.)","Any other drugs to get high; please specify:")**

Shortname / Alias: subst_freq

Piping: Piped From Question 158. (**In the past 12 months (since [question('value'), id='4']), which drugs that were not prescribed to you did you use? (Select all that you have taken in the past 12 months)**)

#### ****In the past 12 months (since [question('value'), id='4']), how often did you use [question('piped title')]?****

( ) Less than once a month

( ) Once a month

( ) More than once a month

( ) Once a week

( ) More than once a week

( ) Once a day

( ) More than once a day

( ) Prefer not to answer

( ) Don't know

## Injection Drug Use

**Logic: Show/hide trigger exists.**

Shortname / Alias: ever_inject

#### ****Have you ever in your life shot up or injected any drugs other than those prescribed for you?**** By shooting up, we mean anytime you might have used a needle to inject drugs in your veins, under the skin, or in the muscle.

( ) Yes

( ) No

( ) Prefer not to answer

( ) Don't know

**Logic: Show/hide trigger exists. Hidden unless: #159 Question "Have you ever in your life shot up or injected any drugs other than those prescribed for you? By shooting up, we mean anytime you might have used a needle to inject drugs in your veins, under the skin, or in the muscle." is one of the following answers ("Yes")**

Shortname / Alias: p12m_inject

#### ****In the past 12 months (since [question('value'), id='4']), about how often did you inject?****

( ) More than once a day

( ) Once a day

( ) More than once a week

( ) Once a week or less

( ) I did not inject in the past 12 months

( ) Prefer not to answer

( ) Don't know

**Logic: Show/hide trigger exists. Hidden unless: #160 Question "In the past 12 months (since [question('value'), id='4']), about how often did you inject?" is one of the following answers ("More than once a day","Once a day","More than once a week","Once a week or less")**

Shortname / Alias: p12m_needleshare

#### ****In the past 12 months, have you injected by using needles, syringes, or other drug preparation equipment (works) that had already been used by another person?****

( ) Yes

( ) No

( ) Prefer not to answer

( ) Don't know

**Logic: Hidden unless: #161 Question "In the past 12 months, have you injected by using needles, syringes, or other drug preparation equipment (works) that had already been used by another person?" is one of the following answers ("Yes")**

Shortname / Alias: shareneedle_hivpos

#### ****Did any of the people that shared their needles with you have a positive HIV status or an HIV status that was unknown to you?****

( ) Yes

( ) No

( ) Prefer not to answer

( ) Don't know

## AUDIT-C

### ****A standard drink is typically 12 ounces of regular beer, 8-9 ounces of malt liquor, 5 ounces of wine, or 1.5 ounces of distilled spirits (hard alcohol). See the picture below for reference.****

**Logic: Show/hide trigger exists.**

Shortname / Alias: alc_drink_freq

#### ****How often do you have a drink containing alcohol?****

( ) Never

( ) Monthly

( ) 2-4 times a month

( ) 2-3 times a week

( ) 4 or more times a week

( ) Prefer not to answer

**Logic: Hidden unless: #163 Question "How often do you have a drink containing alcohol?" is one of the following answers ("Monthly","2-4 times a month","2-3 times a week","4 or more times a week")**

Shortname / Alias: alc_drink_perday

#### ****When you consume alcohol, how many standard drinks do you have in one sitting?****

( ) 1 to 2

( ) 3 to 4

( ) 5 to 6

( ) 7 to 9

( ) 10 or more

**Logic: Hidden unless: #163 Question "How often do you have a drink containing alcohol?" is one of the following answers ("Monthly","2-4 times a month","2-3 times a week","4 or more times a week")**

Shortname / Alias: alc_6drink_freq

#### ****How often do you have six or more drinks on one occasion?****

( ) Daily or almost daily

( ) Weekly

( ) Monthly

( ) Less than monthly

( ) Never

## GIF5

###

## Stigma - PART 1

### ****Now we're going to ask you some questions about stigma, discrimination and violence that you may have experienced ever in your life and if so, whether they happened in the past 12 months (since [question('value'), id='4']). We know that experiences of mistreatment can be hard to discuss. Remember, you do not have to answer any question if you don't want to, and your responses will remain private and entirely confidential.**** ****The next set of questions is about whether experiences happened ever in your life, and if so, whether they happened in the past 12 months, (since [question('value'), id='4']).****

Shortname / Alias: stigma_exclude

#### ****Have you ever felt excluded from family activities because you have sex with men?****

( ) Yes, in the last 12 months

( ) Yes, but not in the last 12 months

( ) No

( ) Prefer not to answer

( ) Don't know

Shortname / Alias: stigma_discrimremark

#### ****Have you ever felt that family members have made discriminatory remarks or gossiped about you because you have sex with men?****

( ) Yes, in the last 12 months

( ) Yes, but not in the last 12 months

( ) No

( ) Prefer not to answer

( ) Don't know

Shortname / Alias: stigma_friend_reject

#### ****Have you ever felt rejected by your friends because you have sex with men?****

( ) Yes, in the last 12 months

( ) Yes, but not in the last 12 months

( ) No

( ) Prefer not to answer

( ) Don't know

Shortname / Alias: stigma_afraid_care

#### ****Have you ever felt afraid to seek out health care services because you worry someone may treat you poorly because you have sex with men?****

( ) Yes, in the last 12 months

( ) Yes, but not in the last 12 months

( ) No

( ) Prefer not to answer

( ) Don't know

Shortname / Alias: stigma_avoid_care

#### ****Have you ever avoided attending or going to health care services because you worry someone may treat you poorly because you have sex with men?****

( ) Yes, in the last 12 months

( ) Yes, but not in the last 12 months

( ) No

( ) Prefer not to answer

( ) Don't know

Shortname / Alias: stigma_care_gossip

#### ****Have you ever heard health care providers gossiping about you (talking about you) because you have sex with men?****

( ) Yes, in the last 12 months

( ) Yes, but not in the last 12 months

( ) No

( ) Prefer not to answer

( ) Don't know

Shortname / Alias: stigma_unwell_care

#### ****Have you ever felt that you were not treated well in a health center because someone knew that you have sex with men?****

( ) Yes, in the last 12 months

( ) Yes, but not in the last 12 months

( ) No

( ) Prefer not to answer

( ) Don't know

Shortname / Alias: stigma_healthcare_judge

#### ****Have you ever worried that a healthcare provider would judge you for having sex with men?****

( ) Yes, in the last 12 months

( ) Yes, but not in the last 12 months

( ) No

( ) Prefer not to answer

( ) Don't know

## Stigma - PART 2

Shortname / Alias: stigma_healthcare_treatdiff

#### ****Have you ever worried that a healthcare provider would treat you differently if you told them you have had sex with men?****

( ) Yes, in the last 12 months

( ) Yes, but not in the last 12 months

( ) No

( ) Prefer not to answer

( ) Don't know

Shortname / Alias: stigma_healthcare_uncomfort

#### ****Have you ever been concerned you would make a healthcare provider uncomfortable talking about having sex with men?****

( ) Yes, in the last 12 months

( ) Yes, but not in the last 12 months

( ) No

( ) Prefer not to answer

( ) Don't know

Shortname / Alias: stigma_police

#### ****Have you ever felt that the police refused to protect you because you have sex with men?****

( ) Yes, in the last 12 months

( ) Yes, but not in the last 12 months

( ) No

( ) Prefer not to answer

( ) Don't know

Shortname / Alias: stigma_scared_public

#### ****Have you ever felt scared to be in public places because you have sex with men?****

( ) Yes, in the last 12 months

( ) Yes, but not in the last 12 months

( ) No

( ) Prefer not to answer

( ) Don't know

Shortname / Alias: stigma_verbal_harass

#### ****Have you ever been verbally harassed and felt it was because you have sex with men?****

( ) Yes, in the last 12 months

( ) Yes, but not in the last 12 months

( ) No

( ) Prefer not to answer

( ) Don't know

**Logic: Show/hide trigger exists.**

Shortname / Alias: stigma_physical

#### ****Has someone ever physically hurt you (pushed, shoved, slapped, hit, kicked, choked or otherwise physically hurt you)?****

( ) Yes, in the last 12 months

( ) Yes, but not in the last 12 months

( ) No

( ) Prefer not to answer

( ) Don't know

**Logic: Hidden unless: #179 Question "Has someone ever physically hurt you (pushed, shoved, slapped, hit, kicked, choked or otherwise physically hurt you)?" is one of the following answers ("Yes, in the last 12 months","Yes, but not in the last 12 months")**

Shortname / Alias: stigma_physical_msm

#### ****Do you believe any of these experiences of physical violence was/were related to the fact that you have sex with men?****

( ) Yes

( ) No

( ) Prefer not to answer

( ) Don't know

**Logic: Show/hide trigger exists.**

Shortname / Alias: stigma_forcesex

#### ****Have you ever been forced to have sex when you did not want to?**** By forced, we mean physically forced, coerced to have sex, or penetrated with an object, when you did not want to.

( ) Yes, in the last 12 months

( ) Yes, but not in the last 12 months

( ) No

( ) Prefer not to answer

( ) Don't know

**Logic: Hidden unless: #181 Question "Have you ever been forced to have sex when you did not want to? By forced, we mean physically forced, coerced to have sex, or penetrated with an object, when you did not want to." is one of the following answers ("Yes, in the last 12 months","Yes, but not in the last 12 months")**

Shortname / Alias: stigma_forcesex_msm

#### ****Do you believe any of these experiences of sexual violence were related to the fact that you have sex with men?****

( ) Yes

( ) No

( ) Prefer not to answer

( ) Don't know

## Stigma: Outness

**Logic: Show/hide trigger exists.**

Shortname / Alias: stigma_outness_toldmsm

#### ****Have you ever told anyone that you are attracted to or have sex with men?****

( ) Yes

( ) No

( ) Prefer not to answer

( ) Don't know

**Logic: Hidden unless: #183 Question "Have you ever told anyone that you are attracted to or have sex with men?" is one of the following answers ("Yes")**

Shortname / Alias: told_msm_

#### ****Which of the following people have you told that you are attracted to or have sex with men?****

|  | **None** | **Some** | **All** | **Does not apply** |
| --- | --- | --- | --- | --- |
| Gay, lesbian, or bisexual friends | ( ) | ( ) | ( ) | ( ) |
| Friends who are not gay, lesbian, or bisexual | ( ) | ( ) | ( ) | ( ) |
| Family members | ( ) | ( ) | ( ) | ( ) |
| Health care providers | ( ) | ( ) | ( ) | ( ) |
| Employer | ( ) | ( ) | ( ) | ( ) |
| Fellow employees | ( ) | ( ) | ( ) | ( ) |

Validation: Min = 0 Max = 100

Shortname / Alias: lgbt_percentage

#### ****Among your friends and acquaintances, what percentage are LGBT?**** Provide your best guess.

0 ________________________[__]_____________________________ 100

## Stigma: Stigma & Tolerance

Shortname / Alias: p12m_msm_

#### ****During the past 12 months (since [question('value'), id='4']), have any of the following things happened to you because someone knew or assumes you were attracted to men?****

|  | **No** | **Yes** | **Prefer not to answer** | **Don't know** | **Does not apply** |
| --- | --- | --- | --- | --- | --- |
| You were called names or insulted | ( ) | ( ) | ( ) | ( ) | ( ) |
| You received poorer services than other people in restaurants, stores, other businesses or agencies | ( ) | ( ) | ( ) | ( ) | ( ) |
| You were treated unfairly at work or school | ( ) | ( ) | ( ) | ( ) | ( ) |
| You were denied or given lower quality health care | ( ) | ( ) | ( ) | ( ) | ( ) |
| You were physically attacked or injured | ( ) | ( ) | ( ) | ( ) | ( ) |

Shortname / Alias: area_tolerant

#### ****How strongly do you agree or disagree with the following statement: "Most people in my area are tolerant of gay and bisexual people."****

( ) Strongly agree

( ) Agree

( ) Neither agree nor disagree

( ) Disagree

( ) Strongly disagree

( ) Prefer not to answer

( ) Don't know

Shortname / Alias: patient_deceive

#### ****How strongly do you agree or disagree with the following statement: "Patients have sometimes been deceived or misled by health care organizations."****

( ) Strongly agree

( ) Agree

( ) Neither agree nor disagree

( ) Disagree

( ) Strongly disagree

( ) Prefer not to answer

( ) Don't know

## Stigma: Social & Online Behavior

Shortname / Alias: p12m_gaybar

#### ****In the past 12 months (since [question('value'), id='4']), how often have you gone to a place (not online) where gay men hang out, meet, or socialize? These could include bars, clubs, social organizations, parks, gay businesses, bookstores, sex clubs, etc.****

( ) More than once a day

( ) Once a day

( ) More than once a week

( ) Once a week

( ) More than once a month

( ) Once a month

( ) Less than once a month

( ) Prefer not to answer

( ) Don't know

**Logic: Show/hide trigger exists.**

Shortname / Alias: p12m_internet_

#### ****In the past 12 months, have you used any of the following kinds of internet sites to meet or socialize with gay men?**** Check all that apply.

[ ] Social network websites (such as Instagram or TikTok)

[ ] Dating websites directed towards gay men

[ ] Mobile phone apps (such as gay chat, dating, and hookup apps)

[ ] None of the above

[ ] Prefer not to answer

[ ] Don't know

**Logic: Hidden unless: #190 Question "In the past 12 months, have you used any of the following kinds of internet sites to meet or socialize with gay men? Check all that apply." is one of the following answers ("Social network websites (such as Instagram or TikTok)")**

Shortname / Alias: p12m_social_freq

#### ****In the past 12 months, how often did you use social network websites (such as Facebook) to meet or socialize with gay men?**** Check all that apply.

( ) More than once a day

( ) Once a day

( ) More than once a week

( ) Once a week

( ) More than once a month

( ) Once a month

( ) Less than once a month

( ) Prefer not to answer

( ) Don't know

**Logic: Hidden unless: #190 Question "In the past 12 months, have you used any of the following kinds of internet sites to meet or socialize with gay men? Check all that apply." is one of the following answers ("Dating websites directed towards gay men")**

Shortname / Alias: p12m_datingweb_freq

#### ****In the past 12 months, how often have you used dating websites directed towards gay men to meet or socialize with gay men?**** Check all that apply.

( ) More than once a day

( ) Once a day

( ) More than once a week

( ) Once a week

( ) More than once a month

( ) Once a month

( ) Less than once a month

( ) Prefer not to answer

( ) Don't know

**Logic: Hidden unless: #190 Question "In the past 12 months, have you used any of the following kinds of internet sites to meet or socialize with gay men? Check all that apply." is one of the following answers ("Mobile phone apps (such as gay chat, dating, and hookup apps)")**

Shortname / Alias: p12m_app_freq

#### ****In the past 12 months, how often did you use mobile phone apps (such as gay chat, dating, and hookup apps) to meet or socialize with gay men?**** Check all that apply.

( ) More than once a day

( ) Once a day

( ) More than once a week

( ) Once a week

( ) More than once a month

( ) Once a month

( ) Less than once a month

( ) Prefer not to answer

( ) Don't know

## Stigma: Discrimination (Shortened Everyday Discrimination Scale & Abrev. Major Experiences of Discrimination Scale)

Shortname / Alias: daytoday_

#### ****In your day-to-day life how often have any of the following things happened to you?****

|  | **Almost everyday** | **At least once a week** | **A few times a month** | **A few times a year** | **Less than once a year** | **Never** |
| --- | --- | --- | --- | --- | --- | --- |
| You are treated with less courtesy or respect than other people. | ( ) | ( ) | ( ) | ( ) | ( ) | ( ) |
| You receive poorer service than other people at restaurants or stores. | ( ) | ( ) | ( ) | ( ) | ( ) | ( ) |
| People act as if they think you are not smart. | ( ) | ( ) | ( ) | ( ) | ( ) | ( ) |
| People act as if they are afraid of you. | ( ) | ( ) | ( ) | ( ) | ( ) | ( ) |
| You are threatened or harassed. | ( ) | ( ) | ( ) | ( ) | ( ) | ( ) |

**Logic: Hidden unless: (((( Question "You are treated with less courtesy or respect than other people." is one of the following answers ("Almost everyday","At least once a week","A few times a month","A few times a year") OR Question "You receive poorer service than other people at restaurants or stores." is one of the following answers ("Almost everyday","At least once a week","A few times a month","A few times a year")) OR Question "People act as if they think you are not smart." is one of the following answers ("Almost everyday","At least once a week","A few times a month","A few times a year")) OR Question "People act as if they are afraid of you." is one of the following answers ("Almost everyday","At least once a week","A few times a month","A few times a year")) OR Question "You are threatened or harassed." is one of the following answers ("Almost everyday","At least once a week","A few times a month","A few times a year"))**

Shortname / Alias: main_reas_dtd_

#### ****What do you think is the main reason for these experiences?**** Check all that apply.

[ ] Your ancestry or national origins

[ ] Your gender

[ ] Your race

[ ] Your age

[ ] Your religion

[ ] Your height

[ ] Your weight

[ ] Some other aspect of your physical appearance

[ ] Your sexual orientation

[ ] Your education or income level

## Stigma: Discrimination - PART 2

### ****In the following questions, we are interested in your perceptions about the way other people have treated you. Can you tell me if any of the following has ever happened to you:****

**Logic: Show/hide trigger exists.**

Shortname / Alias: ever_unfair_fire

#### ****At any time in your life, have you ever been unfairly fired from a job or been unfairly denied a promotion?****

( ) Yes

( ) No

( ) Prefer not to answer

( ) Don't know

**Logic: Hidden unless: #196 Question "At any time in your life, have you ever been unfairly fired from a job or been unfairly denied a promotion?" is one of the following answers ("Yes")**

Shortname / Alias: main_reas_unfair_fire_

#### ****What do you think is the main reason for these experiences?****Check all that apply.

[ ] Your ancestry or national origins

[ ] Your gender

[ ] Your race

[ ] Your age

[ ] Your religion

[ ] Your height

[ ] Your weight

[ ] Some other aspect of your physical appearance

[ ] Your sexual orientation

[ ] Your education or income level

**Logic: Hidden unless: #196 Question "At any time in your life, have you ever been unfairly fired from a job or been unfairly denied a promotion?" is one of the following answers ("Yes")**

Shortname / Alias: last_time_unfair_fire

#### ****When was the last time this happened?****

( ) Past week

( ) Past month

( ) Past year

( ) More than a year ago

Validation: Min = 0 Max = 9999 Must be numeric Whole numbers only Positive numbers only Max character count = 4

**Logic: Hidden unless: #196 Question "At any time in your life, have you ever been unfairly fired from a job or been unfairly denied a promotion?" is one of the following answers ("Yes")**

Shortname / Alias: howmanytimes_unfair_fire

### ****How many times has this happened during your lifetime?****

_________________________________________________

**Logic: Show/hide trigger exists.**

Shortname / Alias: ever_unfair_hire

#### ****For unfair reasons, have you ever not been hired for a job?****

( ) Yes

( ) No

( ) Prefer not to answer

( ) Don't know

**Logic: Hidden unless: #200 Question "For unfair reasons, have you ever not been hired for a job?" is one of the following answers ("Yes")**

Shortname / Alias: main_reas_unfair_hire_

#### ****What do you think is the main reason for these experiences?****Check all that apply.

[ ] Your ancestry or national origins

[ ] Your gender

[ ] Your race

[ ] Your age

[ ] Your religion

[ ] Your height

[ ] Your weight

[ ] Some other aspect of your physical appearance

[ ] Your sexual orientation

[ ] Your education or income level

**Logic: Hidden unless: #200 Question "For unfair reasons, have you ever not been hired for a job?" is one of the following answers ("Yes")**

Shortname / Alias: last_time_unfair_hire

#### ****When was the last time this happened?****

( ) Past week

( ) Past month

( ) Past year

( ) More than a year ago

Validation: Min = 0 Max = 9999 Must be numeric Whole numbers only Positive numbers only Max character count = 4

**Logic: Hidden unless: #200 Question "For unfair reasons, have you ever not been hired for a job?" is one of the following answers ("Yes")**

Shortname / Alias: howmanytimes_unfair_hire

### ****How many times has this happened during your lifetime?****

_________________________________________________

## Stigma: Discrimination - PART 3

**Logic: Show/hide trigger exists.**

Shortname / Alias: ever_unfair_stop

#### ****Have you ever been unfairly stopped, searched, questioned, physically threatened or abused by the police?****

( ) Yes

( ) No

( ) Prefer not to answer

( ) Don't know

**Logic: Hidden unless: #204 Question "Have you ever been unfairly stopped, searched, questioned, physically threatened or abused by the police?" is one of the following answers ("Yes")**

Shortname / Alias: main_reas_unfair_stop_

#### ****What do you think is the main reason for these experiences?****Check all that apply.

[ ] Your ancestry or national origins

[ ] Your gender

[ ] Your race

[ ] Your age

[ ] Your religion

[ ] Your height

[ ] Your weight

[ ] Some other aspect of your physical appearance

[ ] Your sexual orientation

[ ] Your education or income level

**Logic: Hidden unless: #204 Question "Have you ever been unfairly stopped, searched, questioned, physically threatened or abused by the police?" is one of the following answers ("Yes")**

Shortname / Alias: last_time_unfair_stop

#### ****When was the last time this happened?****

( ) Past week

( ) Past month

( ) Past year

( ) More than a year ago

Validation: Min = 0 Max = 9999 Must be numeric Whole numbers only Positive numbers only Max character count = 4

**Logic: Hidden unless: #204 Question "Have you ever been unfairly stopped, searched, questioned, physically threatened or abused by the police?" is one of the following answers ("Yes")**

Shortname / Alias: howmanytimes_unfair_stop

### ****How many times has this happened during your lifetime?****

_________________________________________________

**Logic: Show/hide trigger exists.**

Shortname / Alias: ever_unfair_edu

#### ****Have you ever been unfairly discouraged by a teacher or advisor from continuing your education?****

( ) Yes

( ) No

( ) Prefer not to answer

( ) Don't know

**Logic: Hidden unless: #208 Question "Have you ever been unfairly discouraged by a teacher or advisor from continuing your education?" is one of the following answers ("Yes")**

Shortname / Alias: main_reas_unfair_edu_

#### ****What do you think is the main reason for these experiences?****Check all that apply.

[ ] Your ancestry or national origins

[ ] Your gender

[ ] Your race

[ ] Your age

[ ] Your religion

[ ] Your height

[ ] Your weight

[ ] Some other aspect of your physical appearance

[ ] Your sexual orientation

[ ] Your education or income level

**Logic: Hidden unless: #208 Question "Have you ever been unfairly discouraged by a teacher or advisor from continuing your education?" is one of the following answers ("Yes")**

Shortname / Alias: last_time_unfair_edu

#### ****When was the last time this happened?****

( ) Past week

( ) Past month

( ) Past year

( ) More than a year ago

Validation: Min = 0 Max = 9999 Must be numeric Whole numbers only Positive numbers only Max character count = 4

**Logic: Hidden unless: #208 Question "Have you ever been unfairly discouraged by a teacher or advisor from continuing your education?" is one of the following answers ("Yes")**

Shortname / Alias: howmanytimes_unfair_edu

### ****How many times has this happened during your lifetime?****

_________________________________________________

## Stigma: Discrimination - PART 4

**Logic: Show/hide trigger exists.**

Shortname / Alias: ever_unfair_move

#### ****Have you ever been unfairly prevented from moving into a neighborhood because the landlord or a realtor refused to sell or rent you a house or apartment?****

( ) Yes

( ) No

( ) Prefer not to answer

( ) Don't know

**Logic: Hidden unless: #212 Question "Have you ever been unfairly prevented from moving into a neighborhood because the landlord or a realtor refused to sell or rent you a house or apartment?" is one of the following answers ("Yes")**

Shortname / Alias: main_reas_unfair_move_

#### ****What do you think is the main reason for these experiences?****Check all that apply.

[ ] Your ancestry or national origins

[ ] Your gender

[ ] Your race

[ ] Your age

[ ] Your religion

[ ] Your height

[ ] Your weight

[ ] Some other aspect of your physical appearance

[ ] Your sexual orientation

[ ] Your education or income level

**Logic: Hidden unless: #212 Question "Have you ever been unfairly prevented from moving into a neighborhood because the landlord or a realtor refused to sell or rent you a house or apartment?" is one of the following answers ("Yes")**

Shortname / Alias: last_time_unfair_move

#### ****When was the last time this happened?****

( ) Past week

( ) Past month

( ) Past year

( ) More than a year ago

Validation: Min = 0 Max = 9999 Must be numeric Whole numbers only Positive numbers only Max character count = 4

**Logic: Hidden unless: #212 Question "Have you ever been unfairly prevented from moving into a neighborhood because the landlord or a realtor refused to sell or rent you a house or apartment?" is one of the following answers ("Yes")**

Shortname / Alias: howmanytimes_unfair_move

### ****How many times has this happened during your lifetime?****

_________________________________________________

**Logic: Show/hide trigger exists.**

Shortname / Alias: ever_unfair_loan

#### ****Have you ever been unfairly denied a bank loan?****

( ) Yes

( ) No

( ) Prefer not to answer

( ) Don't know

**Logic: Hidden unless: #216 Question "Have you ever been unfairly denied a bank loan?" is one of the following answers ("Yes")**

Shortname / Alias: main_reas_unfair_loan_

#### ****What do you think is the main reason for these experiences?****Check all that apply.

[ ] Your ancestry or national origins

[ ] Your gender

[ ] Your race

[ ] Your age

[ ] Your religion

[ ] Your height

[ ] Your weight

[ ] Some other aspect of your physical appearance

[ ] Your sexual orientation

[ ] Your education or income level

**Logic: Hidden unless: #216 Question "Have you ever been unfairly denied a bank loan?" is one of the following answers ("Yes")**

Shortname / Alias: last_time_unfair_loan

#### ****When was the last time this happened?****

( ) Past week

( ) Past month

( ) Past year

( ) More than a year ago

Validation: Min = 0 Max = 9999 Must be numeric Whole numbers only Positive numbers only Max character count = 4

**Logic: Hidden unless: #216 Question "Have you ever been unfairly denied a bank loan?" is one of the following answers ("Yes")**

Shortname / Alias: howmanytimes_unfair_loan

### ****How many times has this happened during your lifetime?****

_________________________________________________

## Mental Health: PHQ-9

### ****Now we're going to ask you some questions about your mood. When answering, please think about how often the following has occurred during the past 2 weeks.****

Shortname / Alias: phq9_

#### ****Over the past 2 weeks, how often have you been bothered by the following problems?****

|  | **Not at all** | **Several days** | **More than half the days** | **Nearly every day** | **Prefer not to answer** | **Don't know** |
| --- | --- | --- | --- | --- | --- | --- |
| Little interest or pleasure in doing things | ( ) | ( ) | ( ) | ( ) | ( ) | ( ) |
| Feeling down, depressed, or hopeless | ( ) | ( ) | ( ) | ( ) | ( ) | ( ) |
| Trouble falling or staying asleep, or sleeping too much | ( ) | ( ) | ( ) | ( ) | ( ) | ( ) |
| Feeling tired or having little energy | ( ) | ( ) | ( ) | ( ) | ( ) | ( ) |
| Poor appetite or overeating | ( ) | ( ) | ( ) | ( ) | ( ) | ( ) |
| Feeling bad about yourself – or that you are a failure or have let yourself or your family down | ( ) | ( ) | ( ) | ( ) | ( ) | ( ) |
| Trouble concentrating on things, such as reading the newspaper or watching television | ( ) | ( ) | ( ) | ( ) | ( ) | ( ) |
| Moving or speaking so slowly that other people could have noticed. Or the opposite – being so fidgety or restless that you have been moving around a lot more than usual | ( ) | ( ) | ( ) | ( ) | ( ) | ( ) |
| Thoughts that you would be better off dead, or of hurting yourself | ( ) | ( ) | ( ) | ( ) | ( ) | ( ) |

## Mental Health: GAD-7

Shortname / Alias: gad7_

#### ****Over the past 2 weeks, how often have you been bothered by the following problems?****

|  | **Not at all** | **Several days** | **More than half the days** | **Nearly every day** | **Prefer not to answer** | **Don't know** |
| --- | --- | --- | --- | --- | --- | --- |
| Feeling nervous, anxious, or on edge | ( ) | ( ) | ( ) | ( ) | ( ) | ( ) |
| Not being able to stop or control worrying | ( ) | ( ) | ( ) | ( ) | ( ) | ( ) |
| Worrying too much about different things | ( ) | ( ) | ( ) | ( ) | ( ) | ( ) |
| Trouble relaxing | ( ) | ( ) | ( ) | ( ) | ( ) | ( ) |
| Being so restless that it is hard to sit still | ( ) | ( ) | ( ) | ( ) | ( ) | ( ) |
| Becoming easily annoyed or irritable | ( ) | ( ) | ( ) | ( ) | ( ) | ( ) |
| Feeling afraid, as if something awful might happen | ( ) | ( ) | ( ) | ( ) | ( ) | ( ) |

## Mental Health: Resiliency & Self-Efficacy

Shortname / Alias: resiliency_

#### ****Please read the following questions and check the boxes that indicate how you feel about yourself.****

|  | **Not at all true** | **Hardly true** | **Moderately true** | **Exactly true** | **Prefer not to answer** | **Don't know** |
| --- | --- | --- | --- | --- | --- | --- |
| I can always manage to solve difficult problems if I try hard enough. | ( ) | ( ) | ( ) | ( ) | ( ) | ( ) |
| If someone opposes me, I can find the means and ways to get what I want. | ( ) | ( ) | ( ) | ( ) | ( ) | ( ) |
| It is easy for me to stick to my aims and accomplish my goals. | ( ) | ( ) | ( ) | ( ) | ( ) | ( ) |
| I am confident that I could deal efficiently with unexpected events. | ( ) | ( ) | ( ) | ( ) | ( ) | ( ) |
| Thanks to my resourcefulness, I know how to handle unforeseen situations. | ( ) | ( ) | ( ) | ( ) | ( ) | ( ) |
| I can solve most problems if I invest the necessary effort. | ( ) | ( ) | ( ) | ( ) | ( ) | ( ) |
| I can remain calm when facing difficulties because I can rely on my coping abilities. | ( ) | ( ) | ( ) | ( ) | ( ) | ( ) |
| When I am confronted with a problem, I can usually find several solutions. | ( ) | ( ) | ( ) | ( ) | ( ) | ( ) |
| If I am in trouble, I can usually think of a solution. | ( ) | ( ) | ( ) | ( ) | ( ) | ( ) |

## Mental Health: Emotional & Instrumental Support

Shortname / Alias: support1_

#### ****People sometimes look to others for companionship, assistance, or other types of support. Check the boxes to indicate how often each of the following kinds of support is available to you if you need it.****

|  | **Never** | **Rarely** | **Sometimes** | **Usually** | **Always** | **Prefer not to answer** | **Don't know** |
| --- | --- | --- | --- | --- | --- | --- | --- |
| I have someone who will listen to me when I need to talk. | ( ) | ( ) | ( ) | ( ) | ( ) | ( ) | ( ) |
| I have someone to confide in or talk to about myself or my problems. | ( ) | ( ) | ( ) | ( ) | ( ) | ( ) | ( ) |
| I have someone who makes me feel appreciated. | ( ) | ( ) | ( ) | ( ) | ( ) | ( ) | ( ) |
| I have someone to talk with when I have a bad day. | ( ) | ( ) | ( ) | ( ) | ( ) | ( ) | ( ) |

Shortname / Alias: support2_

####

|  | **Never** | **Rarely** | **Sometimes** | **Usually** | **Always** | **Prefer not to answer** | **Don't know** |
| --- | --- | --- | --- | --- | --- | --- | --- |
| I have someone to give me good advice about a crisis if I need it. | ( ) | ( ) | ( ) | ( ) | ( ) | ( ) | ( ) |
| I have someone to turn to for suggestions about how to deal with a problem. | ( ) | ( ) | ( ) | ( ) | ( ) | ( ) | ( ) |
| I have someone to give me information if I need it. | ( ) | ( ) | ( ) | ( ) | ( ) | ( ) | ( ) |
| I get useful advice about important things in life. | ( ) | ( ) | ( ) | ( ) | ( ) | ( ) | ( ) |

Shortname / Alias: support3_

####

|  | **Never** | **Rarely** | **Sometimes** | **Usually** | **Always** | **Prefer not to answer** | **Don't know** |
| --- | --- | --- | --- | --- | --- | --- | --- |
| Do you have someone to help you if you are confined to bed? | ( ) | ( ) | ( ) | ( ) | ( ) | ( ) | ( ) |
| Do you have someone to take you to the doctor if you need it? | ( ) | ( ) | ( ) | ( ) | ( ) | ( ) | ( ) |
| Do you have someone to help with your daily chores if you are sick? | ( ) | ( ) | ( ) | ( ) | ( ) | ( ) | ( ) |
| Do you have someone to run errands if you need it? | ( ) | ( ) | ( ) | ( ) | ( ) | ( ) | ( ) |

## Mental Health: Companionship & Social Isolation

Shortname / Alias: companion_

#### ****People sometimes look to others for companionship, assistance, or other types of support. Check the boxes to indicate how often each of the following kinds of support is available to you if you need it.****

|  | **Never** | **Rarely** | **Sometimes** | **Usually** | **Always** | **Prefer not to answer** | **Don't know** |
| --- | --- | --- | --- | --- | --- | --- | --- |
| Do you have someone with whom to have fun? | ( ) | ( ) | ( ) | ( ) | ( ) | ( ) | ( ) |
| Do you have someone with whom to relax? | ( ) | ( ) | ( ) | ( ) | ( ) | ( ) | ( ) |
| Do you have someone with whom you can do something enjoyable? | ( ) | ( ) | ( ) | ( ) | ( ) | ( ) | ( ) |
| Do you find companionship when you want it? | ( ) | ( ) | ( ) | ( ) | ( ) | ( ) | ( ) |

Shortname / Alias: iso_

#### ****Please check the following boxes to indicate how often the following statements apply to you.****

|  | **Never** | **Rarely** | **Sometimes** | **Usually** | **Always** | **Prefer not to answer** | **Don't know** |
| --- | --- | --- | --- | --- | --- | --- | --- |
| I feel left out. | ( ) | ( ) | ( ) | ( ) | ( ) | ( ) | ( ) |
| I feel that people barely know me. | ( ) | ( ) | ( ) | ( ) | ( ) | ( ) | ( ) |
| I feel isolated from others. | ( ) | ( ) | ( ) | ( ) | ( ) | ( ) | ( ) |
| I feel that people are around me but not with me. | ( ) | ( ) | ( ) | ( ) | ( ) | ( ) | ( ) |

## Incorrectly Answered Questions

### ****You are almost finished!**** We understand that having a survey without a “back button” can be difficult because it does not allow you to make corrections to previous responses.

Shortname / Alias: correction_essay

### If you would like to change anything about your responses, or questions we should have asked, please describe the question(s) and what the response(s) should be, using the space below.

____________________________________________

____________________________________________

____________________________________________

____________________________________________

## Closing Language and Gift Card Preference

### ****You're almost done! Just one final question.****

Shortname / Alias: gift_card_pref

#### ****Once we validate your survey, we will send your $50 electronic gift card to your email address. Please let us know which gift card you would prefer.*****

( ) Amazon

( ) Target

( ) Walmart

## Thank You!

### Thank you for completing the survey! We really appreciate your participation in the Combine Study. We will be in touch in the next couple of days to confirm your participation in the study and answer any remaining questions. If you have any questions in the meantime, you can contact us via email at [combine_rct@emory.edu](mailto:combine_rct@emory.edu).
