# Supplementary material for: Evaluating the Effectiveness of a Mobile HIV Prevention App to Increase HIV and Sexually Transmitted Infection Testing and Pre-Exposure Prophylaxis Initiation Among Rural Men Who Have Sex With Men in the Southern United States: Protocol for a Randomized Controlled Trial
Source: JMIR Res Protoc. 2025 Jul 23;14:e69540. doi: 10.2196/69540 (PMC12329388; doi:10.2196/69540)
Supplement: Multimedia Appendix 4 [file resprot_v14i1e69540_app4.docx]

# COMBINE RCT 6 Month Follow-Up

## Dates and Pulled Variables

## SGUID Check

## Landing Page / Intro

### Welcome back! Please complete this follow-up survey. It will take about 45 minutes to complete. Once we can validate your responses, you will receive a $50 eGift Card to your choice of Amazon, Target, or Walmart. If you have any questions, please email [combine_rct@emory.edu](mailto:combine_rct@emory.edu) or call 470-870-6809. We are going to be asking questions about various topics, including your background, life experience, healthcare, your sexual partners and relationships, and substance use. You are not required to answer any questions in this survey. If you do not answer a question, you may receive a prompt to be sure that you meant to leave the question unanswered before you move forward. This is a forward-only survey. When you finish a page, continue to the next page by clicking the "Next" button. You may not go backwards to pages you have already completed. We understand that having a survey without a “back button” can be difficult because it does not allow you to make corrections to your answers. There will be a question at the end of the survey to let us know of any corrections you would like to make. If you feel like you have made a mistake, you can also contact the study team ([combine_rct@emory.edu](mailto:combine_rct@emory.edu)). Your privacy is important to us. All information you provide in this survey will be held confidentially. Your answers will be used only for research purposes.

## GIF1

### ****Let's get to it!****

###

## Demographics

**Logic: Show/hide trigger exists.**

Shortname / Alias: last_survey_change_

#### ****Have you had a change in any of the following since your last survey? Please check all that apply.****

[ ] Gender identity

[ ] Education status

[ ] Employment status

[ ] Housing (e.g., you moved)

[ ] Health insurance

[ ] None of these

**Logic: Hidden unless: #1 Question "Have you had a change in any of the following since your last survey? Please check all that apply." is one of the following answers ("Gender identity")**

Shortname / Alias: gender_

#### ****How would you describe your gender identity now? Select all that apply.****

[ ] Male

[ ] Female

[ ] Trans woman, trans female, or transfeminine

[ ] Trans man, trans male, or transmasculine

[ ] Nonbinary, gender nonconforming, or genderqueer

[ ] Another gender

[ ] Prefer not to answer

[ ] Don't know

**Logic: Hidden unless: #1 Question "Have you had a change in any of the following since your last survey? Please check all that apply." is one of the following answers ("Education status")**

Shortname / Alias: hlevedu

#### ****What is the highest level of education you have completed?****

( ) Never attended school

( ) Less than high school

( ) Some high school

( ) High school diploma or GED

( ) Some college, Associate's Degree, or Technical Degree

( ) College graduate

( ) Post graduate or professional school

( ) Prefer not to answer

( ) Don't know

**Logic: Show/hide trigger exists. Hidden unless: #1 Question "Have you had a change in any of the following since your last survey? Please check all that apply." is one of the following answers ("Education status")**

Shortname / Alias: curr_enroll

#### ****Are you currently enrolled in school?****

( ) No

( ) Yes, full time

( ) Yes, part time

( ) Prefer not to answer

( ) Don't know

**Logic: Hidden unless: #4 Question "Are you currently enrolled in school?" is one of the following answers ("Yes, full time","Yes, part time")**

Shortname / Alias: curr_enroll_type

#### ****Which type of school are you currently enrolled in?****

( ) Technical school/trade school

( ) Undergraduate (two-year or four-year program)

( ) Graduate, post graduate or professional school

( ) Other:: _________________________________________________*

( ) Prefer not to answer

( ) Don't know

## Demographics - PART 2

**Logic: Hidden unless: #1 Question "Have you had a change in any of the following since your last survey? Please check all that apply." is one of the following answers ("Employment status")**

Shortname / Alias: curr_employ

#### ****Which best describes your current employment status?****

( ) Employed for wages full-time

( ) Employed for wages part-time

( ) Self-employed

( ) A homemaker

( ) Retired

( ) Not employed but searching for work

( ) Not employed and not actively seeking work

( ) Unable to work (disabled)

( ) Full-time student

( ) Prefer not to answer

( ) Don't know

Shortname / Alias: transport

#### ****Do you have access to a car, truck, or other form of transportation to get wherever you need to go?****

( ) Yes

( ) No

( ) Prefer not to answer

( ) Don't know

**Logic: Hidden unless: #1 Question "Have you had a change in any of the following since your last survey? Please check all that apply." is one of the following answers ("Housing (e.g., you moved)")**

Shortname / Alias: state

#### ****What state do you live in?****

( ) Alabama

( ) Alaska

( ) Arizona

( ) Arkansas

( ) California

( ) Colorado

( ) Connecticut

( ) Delaware

( ) Florida

( ) Georgia

( ) Hawaii

( ) Idaho

( ) Illinois

( ) Indiana

( ) Iowa

( ) Kansas

( ) Kentucky

( ) Louisiana

( ) Maine

( ) Maryland

( ) Massachusetts

( ) Michigan

( ) Minnesota

( ) Mississippi

( ) Missouri

( ) Montana

( ) Nebraska

( ) Nevada

( ) New Hampshire

( ) New Jersey

( ) New Mexico

( ) New York

( ) North Carolina

( ) North Dakota

( ) Ohio

( ) Oklahoma

( ) Oregon

( ) Pennsylvania

( ) Rhode Island

( ) South Carolina

( ) South Dakota

( ) Tennessee

( ) Texas

( ) Utah

( ) Vermont

( ) Virginia

( ) Washington

( ) Washington, D.C.

( ) West Virginia

( ) Wisconsin

( ) Wyoming

Validation: %s format expected Using custom RegEx pattern

**Logic: Hidden unless: #1 Question "Have you had a change in any of the following since your last survey? Please check all that apply." is one of the following answers ("Housing (e.g., you moved)")**

Shortname / Alias: zip_code

### ****What is your ZIP code?****

_________________________________________________

## Demographics - PART 3

Shortname / Alias: p6m_unstable_house

#### ****In the past 6 months (since [question('value'), id='3']), did you stay overnight with friends, relatives, or someone you didn’t know well because you didn’t have a regular, adequate, and safe place to stay at night?****

( ) Yes

( ) No

( ) Prefer not to answer

( ) Don't know

Shortname / Alias: p6m_homeless

#### ****In the past 6 months, were you ever homeless? That is, were you living on the street, in a shelter, in a hotel room, or in a car?****

( ) Yes

( ) No

( ) Prefer not to answer

( ) Don't know

**Logic: Show/hide trigger exists.**

Shortname / Alias: p6m_food_instability

#### ****In the past 6 months, did you or other adults in the household ever cut the size of your meals or skip meals because there wasn't enough money for food?****

( ) Yes

( ) No

( ) Prefer not to answer

( ) Don't know

**Logic: Hidden unless: #12 Question "In the past 6 months, did you or other adults in the household ever cut the size of your meals or skip meals because there wasn't enough money for food?" is one of the following answers ("Yes")**

Shortname / Alias: p6m_food_ins_freq

#### ****In the past 6 months, how often did you or other adults in the household ever cut the size of your meals or skip meals because there wasn't enough money for food?****

( ) Almost every month

( ) Some months but not every month

( ) Only 1 or 2 months

( ) Prefer not to answer

( ) Don't know

Shortname / Alias: p6m_jail

#### ****In the past 6 months, how many different times were you in jail, detention, or prison for longer than 24 hours?****

( ) 0

( ) 1

( ) 2

( ) 3

( ) 4

( ) 5+

( ) Prefer not to answer

( ) Don't know

## Health Care Access and Use

**Logic: Hidden unless: #1 Question "Have you had a change in any of the following since your last survey? Please check all that apply." is one of the following answers ("Health insurance")**

Shortname / Alias: health_ins_

#### ****What kind of health insurance or health care coverage do you currently have?**** Check all that apply.

[ ] I don't currently have any health insurance

[ ] A private health plan purchased through an employer or school

[ ] A private health plan purchased through an exchange (i.e. Obamacare)

[ ] Covered on parent's insurance policy

[ ] Medicaid or Medicare

[ ] Some other Medical Assistance program

[ ] TRICARE (CHAMPUS)

[ ] Veterans Administration coverage

[ ] Some other health care plan:: _________________________________________________*

[ ] Prefer not to answer

[ ] Don't know

**Logic: Show/hide trigger exists.**

Shortname / Alias: p6m_nurse

#### ****In the past 6 months (since [question('value'), id='3']), have you seen a doctor, nurse, or other health care provider about your own health?****

( ) Yes

( ) No

( ) Prefer not to answer

( ) Don't know

**Logic: Hidden unless: #16 Question "In the past 6 months (since [question('value'), id='3']), have you seen a doctor, nurse, or other health care provider about your own health?" is one of the following answers ("Yes")**

Shortname / Alias: provider_type_

#### ****What type of healthcare provider have you visited?**** Please check all that apply

[ ] Primary care provider

[ ] Urgent care clinic

[ ] Emergency room

[ ] Sexual health clinic

[ ] Mental health provider

[ ] Other:: _________________________________________________*

**Logic: Hidden unless: #16 Question "In the past 6 months (since [question('value'), id='3']), have you seen a doctor, nurse, or other health care provider about your own health?" is one of the following answers ("Yes")**

Shortname / Alias: visit_reason_

#### ****What was the reason for your visit(s)?**** Please check all that apply.

[ ] Physical exam/regular wellness visit

[ ] Sick visit

[ ] Sexual health/STI testing/HIV testing

[ ] Other:: _________________________________________________*

## Health Care Access and Use - PART 2

Shortname / Alias: reg_provider

#### ****Do you have a regular healthcare provider? That is, someone you see when you are sick or for routine physical exams.****

( ) Yes, I have a regular provider

( ) Yes, I have a regular clinic like an urgent care or CVS Minute Clinic

( ) No regular provider

( ) Prefer not to answer

( ) Don't know

Shortname / Alias: p6m_care_avoid

#### ****In the past 6 months (since [question('value'), id='3']), have you ever avoided visiting a healthcare provider because you were concerned about cost or your ability to pay?****

( ) Yes

( ) No

( ) Prefer not to answer

( ) Don't know

Shortname / Alias: p6m_told_provider_msm

#### ****Have you told a healthcare provider in the past 6 months that you are attracted to or have sex with men?****

( ) Yes

( ) No

( ) Prefer not to answer

( ) Don't know

## HIV Testing History

###

**Logic: Show/hide trigger exists.**

Shortname / Alias: p6m_hivtest

#### ****Have you had an HIV test the past 6 months (since [question('value'), id='3'])?****

( ) Yes

( ) No

( ) Prefer not to answer

( ) Don't know

**Logic: Hidden unless: #22 Question "Have you had an HIV test the past 6 months (since [question('value'), id='3'])?" is one of the following answers ("Yes")**

Shortname / Alias: p6m_num_hivtest

#### ****How many HIV tests have you taken in the past 6 months?****

( ) 1

( ) 2

( ) 3

( ) 4

( ) 5

( ) 6

( ) 7+

( ) Prefer not to answer

( ) Don't know

**Page entry logic:** This page will show when: #22 Question "**Have you had an HIV test the past 6 months (since [question('value'), id='3'])?**" is one of the following answers ("Yes")

## HIV Testing - PART 2

Shortname / Alias: HIV_wheretest

#### ****When you most recently got tested, where did you get tested?****

( ) Private doctor's office

( ) HIV counseling and testing site

( ) Public health clinic/community health clinic

( ) Street outreach program/mobile unit

( ) Sexually transmitted disease clinic

( ) Hospital (inpatient)

( ) Correctional facility (jail or prison)

( ) Emergency room

( ) At home

( ) Other, please specify:: _________________________________________________*

( ) Prefer not to answer

( ) Don't know

Shortname / Alias: distance_hivtest

#### ****How far did you travel to obtain your most recent HIV test?****

( ) Less than 10 miles

( ) 10 - 20 miles

( ) More than 20 miles

( ) N/A (e.g., at-home test shipped to home)

( ) Prefer not to answer

( ) Don't know

Shortname / Alias: time_hivtest

#### ****How long did it take you to travel to obtain your most recent HIV test?****

( ) 15 minutes or less

( ) 16 - 30 minutes

( ) 31 - 60 minutes

( ) More than one hour

( ) N/A (e.g., at-home test shipped to home)

( ) Prefer not to answer

( ) Don't know

Shortname / Alias: length_hivtest

#### ****How long did it take to complete your most recent HIV test visit?****

( ) 15 minutes or less

( ) 16 - 30 minutes

( ) 31 - 60 minutes

( ) More than one hour

( ) N/A (e.g., at home test shipped to home)

( ) Prefer not to answer

( ) Don't know

**Logic: Show/hide trigger exists.**

Shortname / Alias: result_test

#### ****What was the result of your most recent HIV test?****

( ) Negative

( ) Positive

( ) Never obtained results

( ) Indeterminate

( ) Prefer not to answer

( ) Don't know

**Page entry logic:** This page will show when: #28 Question "**What was the result of your most recent HIV test?**" is one of the following answers ("Positive")

## Positive HIV Test Email Action and Flag

**Page entry logic:** This page will show when: #22 Question "**Have you had an HIV test the past 6 months (since [question('value'), id='3'])?**" is one of the following answers ("Yes")

## HIV Testing - PART 3

**Logic: Show/hide trigger exists.**

Shortname / Alias: p6m_rapid_test

#### ****FDA-approved rapid self-tests allow people to complete an HIV test anonymously at home and receive their results in as little as 20 minutes. Have you used a self-test to test for HIV in the past 6 months (since [question('value'), id='3'])?****

( ) Yes

( ) No

( ) Prefer not to answer

( ) Don't know

**Logic: Hidden unless: #29 Question "FDA-approved rapid self-tests allow people to complete an HIV test anonymously at home and receive their results in as little as 20 minutes. Have you used a self-test to test for HIV in the past 6 months (since [question('value'), id='3'])?" is one of the following answers ("Yes")**

Shortname / Alias: where_self_test

#### ****Where did you get the most recent HIV self-test that you used?****

( ) Pharmacy

( ) A sex partner

( ) A friend or family member

( ) Research study or government program

( ) Online

( ) Combine Research Study

( ) Other, please specify:: _________________________________________________*

**Logic: Hidden unless: #28 Question "What was the result of your most recent HIV test?" is one of the following answers ("Negative","Never obtained results","Indeterminate","Prefer not to answer","Don't know")**

Shortname / Alias: HIV_testoften

#### ****Do you currently test for HIV regularly (after a given amount of time has passed)?****

( ) Yes

( ) No

( ) Prefer not to answer

( ) Don't know

**Logic: Hidden unless: #28 Question "What was the result of your most recent HIV test?" is one of the following answers ("Negative","Never obtained results","Indeterminate","Prefer not to answer","Don't know")**

Shortname / Alias: HIV_testfreq

#### ****About how often do you test for HIV? Please choose the option that best represents how often you test.****

( ) Every month

( ) Every 3 months

( ) Every 6 months

( ) Once a year

( ) Once every 2 years

( ) Other, please specify:: _________________________________________________*

## HIV Testing History - PART 4

Shortname / Alias: recom_test

#### ****As far as you know, what is the current recommendation for how often gay, bisexual, and other men who have sex with men should test for HIV?****

( ) Every month

( ) Every 3-6 months

( ) Once a year

( ) Less often than once a year

( ) Prefer not to answer

( ) Don't know

**Page entry logic:** This page will show when: #1 Question "**Have you had a change in any of the following since your last survey? Please check all that apply.**" is one of the following answers ("Housing (e.g., you moved)")

## Barrier to Care Scale (BACS)

Shortname / Alias: bacs_

#### ****Please indicate to what extent each of the following circumstances makes it difficult for you to receive the care, services, or opportunities you wish to obtain.****

|  | **No problem at all** | **Very slight problem** | **Somewhat of a problem** | **Major problem** |
| --- | --- | --- | --- | --- |
| Long distances to medical facilities and personnel. | ( ) | ( ) | ( ) | ( ) |
| Medical personnel (e.g. physicians, nurses) who decline to provide direct care to persons with HIV/AIDS. | ( ) | ( ) | ( ) | ( ) |
| The lack of health care professionals who are adequately trained and competent in AIDS care. | ( ) | ( ) | ( ) | ( ) |
| The lack of transportation to access the services I need. | ( ) | ( ) | ( ) | ( ) |
| The shortage of psychologists, social workers, and mental health counsellors who can help address mental health issues. | ( ) | ( ) | ( ) | ( ) |
| The lack of psychological support groups for persons with HIV/AIDS. | ( ) | ( ) | ( ) | ( ) |
| The level of knowledge about HIV/AIDS among residents in the community. | ( ) | ( ) | ( ) | ( ) |
| Community residents’ stigma against persons living with HIV/AIDS. | ( ) | ( ) | ( ) | ( ) |
| The lack of employment opportunities for people living with HIV/AIDS. | ( ) | ( ) | ( ) | ( ) |
| The lack of supportive and understanding work environments for people living with HIV/AIDS. | ( ) | ( ) | ( ) | ( ) |
| My personal financial resources. | ( ) | ( ) | ( ) | ( ) |
| Lack of adequate and affordable housing. | ( ) | ( ) | ( ) | ( ) |

## STI Testing History

### ****Now we're going to ask you about testing for sexually transmitted infections (STIs).****

**Logic: Show/hide trigger exists.**

Shortname / Alias: p6m_stitest

#### ****In the past 6 months (since [question('value'), id='3']), were you tested for the sexually transmitted infections gonorrhea, chlamydia, or syphilis?****

( ) Yes

( ) No

( ) Prefer not to answer

( ) Don't know

**Logic: Hidden unless: #35 Question "In the past 6 months (since [question('value'), id='3']), were you tested for the sexually transmitted infections gonorrhea, chlamydia, or syphilis?" is one of the following answers ("Yes")**

Shortname / Alias: p6m_totalstitest

#### ****In the past 6 months, how many times have you been tested for the sexually transmitted infections gonorrhea, chlamydia, or syphilis?****

( ) 1

( ) 2

( ) 3

( ) 4

( ) 5

( ) 6

( ) 7+

( ) Prefer not to answer

( ) Don't know

**Logic: Hidden unless: #35 Question "In the past 6 months (since [question('value'), id='3']), were you tested for the sexually transmitted infections gonorrhea, chlamydia, or syphilis?" is one of the following answers ("Yes")**

Shortname / Alias: sti_test_

#### ****In the past 6 months, when you were tested for the sexually transmitted infections gonorrhea, chlamydia, or syphilis, what samples did you provide for testing?**** Check all that apply.

[ ] I had my blood drawn

[ ] I gave a urine sample

[ ] I had my rectum (butt) swabbed

[ ] I had my throat swabbed

[ ] Prefer not to answer

[ ] Don't know

**Logic: Hidden unless: #35 Question "In the past 6 months (since [question('value'), id='3']), were you tested for the sexually transmitted infections gonorrhea, chlamydia, or syphilis?" is one of the following answers ("Yes")**

Shortname / Alias: pref_stitest_

#### ****When you were tested for the sexually transmitted infections gonorrhea, chlamydia, or syphilis in the past 6 months, how were you tested?**** Check all that apply.

[ ] I went to an STI clinic, healthcare center, or doctor's office where the provider collected the samples

[ ] I picked up the testing kit at an STI clinic, healthcare center, or doctor's office and I swabbed myself while at home or at another location

[ ] An STI clinic, healthcare center, or doctor's office arranged to have a testing kit mailed to me and I swabbed myself while at home or at another location

[ ] I ordered a testing kit online (such as from Amazon) and I swabbed myself at while at home or at another location

[ ] I ordered a STI test kit through the Combine Study

[ ] Prefer not to answer

[ ] Don't know

**Logic: Hidden unless: #35 Question "In the past 6 months (since [question('value'), id='3']), were you tested for the sexually transmitted infections gonorrhea, chlamydia, or syphilis?" is one of the following answers ("Yes")**

Shortname / Alias: distance_stitest

#### ****How far did you travel to obtain your most recent STI test?****

( ) Less than 10 miles

( ) 10 - 20 miles

( ) More than 20 miles

( ) N/A (e.g., at-home test shipped to home)

( ) Prefer not to answer

( ) Don't know

**Logic: Hidden unless: #35 Question "In the past 6 months (since [question('value'), id='3']), were you tested for the sexually transmitted infections gonorrhea, chlamydia, or syphilis?" is one of the following answers ("Yes")**

Shortname / Alias: time_stitest

#### ****How long did it take you to travel to obtain your most recent STI test?****

( ) 15 minutes or less

( ) 16 - 30 minutes

( ) 31 - 60 minutes

( ) More than one hour

( ) N/A (e.g., at-home test shipped to home)

( ) Prefer not to answer

( ) Don't know

**Logic: Hidden unless: #35 Question "In the past 6 months (since [question('value'), id='3']), were you tested for the sexually transmitted infections gonorrhea, chlamydia, or syphilis?" is one of the following answers ("Yes")**

Shortname / Alias: length_stitest

#### ****How long did it take to complete your most recent STI test visit?****

( ) 15 minutes or less

( ) 16 - 30 minutes

( ) 31 - 60 minutes

( ) More than one hour

( ) N/A (e.g., at home test shipped to home)

( ) Prefer not to answer

( ) Don't know

Shortname / Alias: sti_pref_test

#### ****Many STI clinics and healthcare centers provide in-person STI testing. Looking ahead, what is your preferred approach to getting tested for sexually transmitted infections?**** Choose one option to select your preferred location.

( ) Going to an STI clinic, healthcare center, or doctor’s office where the provider collects the sample

( ) Picking up the testing kit at an STI clinic, healthcare center, or doctor’s office and swabbing myself while at home or at another location

( ) Having an STI clinic, healthcare center, or doctor’s office mail me a testing kit and swabbing myself while at home or at another location

( ) Ordering a testing kit online (such as from Amazon) and swabbing myself while at home or at another location

( ) Something else:: _________________________________________________*

( ) Prefer not to answer

( ) Don't know

**Page entry logic:** This page will show when: #35 Question "**In the past 6 months (since [question('value'), id='3']), were you tested for the sexually transmitted infections gonorrhea, chlamydia, or syphilis?**" is one of the following answers ("Yes")

## Bacterial STI Diagnoses

Shortname / Alias: p6m_sti_diag_

#### ****In the past 6 months (since [question('value'), id='3']), has a doctor, nurse, or other health care provider told you that you had any of the following?**** Check all that apply.

[ ] Gonorrhea

[ ] Chlamydia

[ ] Syphilis

[ ] None of the above

[ ] Prefer not to answer

[ ] Don't know

## PrEP Module

### ****Pre-exposure prophylaxis (PrEP) is taking an antiretroviral medicine, either as a pill (also known as Truvada or Descovy) or as an injection (also known as Apretude), to reduce a person's chance of getting HIV.****

###

**Logic: Show/hide trigger exists.**

Shortname / Alias: current_prep

#### ****Are you currently taking PrEP?****

( ) Yes

( ) No

( ) Prefer not to answer

( ) Don't know

**Logic: Show/hide trigger exists. Hidden unless: #44 Question "Are you currently taking PrEP?" is one of the following answers ("Yes")**

Shortname / Alias: prescript_curr_prep

#### ****Which PrEP prescription medication are you currently taking?****

( ) Truvada/emtricitabine-tenofovir (TDF)

( ) Descovy

( ) Apretude

( ) Prefer not to answer

( ) Don't know

**Logic: Hidden unless: #45 Question "Which PrEP prescription medication are you currently taking?" is one of the following answers ("Apretude")**

Shortname / Alias: apretude_location

#### ****Where do you go to get your Apretude injections?****

( ) Private doctor's office

( ) STI Clinic

( ) Health Center

( ) Pharmacy

( ) At home

( ) Other, please specify:: _________________________________________________*

( ) Prefer not to answer

( ) Don't know

**Logic: Hidden unless: #45 Question "Which PrEP prescription medication are you currently taking?" is one of the following answers ("Apretude")**

Shortname / Alias: apretude_num

#### ****In the past 6 months (since [question('value'), id='3']), how many Apretude injections have you received?****

( ) 1

( ) 2

( ) 3

( ) 4

( ) 5

( ) 6 or more

( ) Prefer not to answer

( ) Don't know

**Logic: Show/hide trigger exists. Hidden unless: #45 Question "Which PrEP prescription medication are you currently taking?" is one of the following answers ("Truvada/emtricitabine-tenofovir (TDF)","Descovy")**

Shortname / Alias: prep_dose_p30d

#### ****In the past 30 days, about how many doses of PrEP did you take?****

( ) Less than 15

( ) 16-29

( ) 30

**Logic: Hidden unless: #48 Question "In the past 30 days, about how many doses of PrEP did you take?" is one of the following answers ("Less than 15","16-29")**

Shortname / Alias: intend_prep_freq

#### ****How frequently do you intend to take PrEP?****

( ) Daily

( ) Only when I have sex

( ) Some other schedule, please specify:: _________________________________________________*

**Logic: Hidden unless: #45 Question "Which PrEP prescription medication are you currently taking?" is one of the following answers ("Apretude")**

Shortname / Alias: apretude_months

#### ****How many months in a row have you been taking Apretude?****

( ) Less than 2 months

( ) 2-6 months

( ) 7-12 months

( ) More than 12 months

( ) Prefer not to answer

( ) Don't know

**Logic: Hidden unless: #45 Question "Which PrEP prescription medication are you currently taking?" is one of the following answers ("Truvada/emtricitabine-tenofovir (TDF)")**

Shortname / Alias: truvada_months

#### ****How many months in a row have you been taking Truvada?****

( ) Less than 2 months

( ) 2-6 months

( ) 7-12 months

( ) More than 12 months

( ) Prefer not to answer

( ) Don't know

**Logic: Hidden unless: #45 Question "Which PrEP prescription medication are you currently taking?" is one of the following answers ("Descovy")**

Shortname / Alias: descovy_months

#### ****How many months in a row have you been taking Descovy?****

( ) Less than 2 months

( ) 2-6 months

( ) 7-12 months

( ) More than 12 months

( ) Prefer not to answer

( ) Don't know

**Logic: Show/hide trigger exists. Hidden unless: #44 Question "Are you currently taking PrEP?" is one of the following answers ("No")**

Shortname / Alias: p6m_prep

#### ****In the past 6 months (since [question('value'), id='3']), have you taken PrEP?****

( ) Yes

( ) No

( ) Prefer not to answer

( ) Don't know

**Logic: Hidden unless: #53 Question "In the past 6 months (since [question('value'), id='3']), have you taken PrEP?" is one of the following answers ("Yes")**

Shortname / Alias: p6m_prep_type_

#### ****In the past 6 months, which PrEP prescription medications did you take?**** Check all that apply.

[ ] Truvada (name brand or generic)

[ ] Descovy

[ ] Apretude

[ ] Prefer not to answer

[ ] Don't know

**Logic: Hidden unless: (#44 Question "Are you currently taking PrEP?" is one of the following answers ("No") AND #53 Question "In the past 6 months (since [question('value'), id='3']), have you taken PrEP?" is one of the following answers ("Yes"))**

Shortname / Alias: prep_stop_reas_

#### ****Which of the following describes the reason(s) why you stopped using PrEP the last time you were on it?**** Check all that apply.

[ ] The cost was too high

[ ] I lost my job and/or insurance

[ ] I experienced and/or was concerned about side-effects

[ ] I could not remember to take the pill every day

[ ] I started a monogamous relationship with an HIV-negative partner

[ ] I stopped being sexually active

[ ] I prefer to use other methods to protect myself from HIV

[ ] I was worried that people will think that I have HIV when they see me taking the pill

[ ] I was worried that people will know that I have sex with men or transgender people

[ ] I was worried people will think I am very sexually active because I am on PrEP

[ ] I was worried about my privacy with someone that I live with

[ ] I was worried about my privacy on my parent’s health insurance plan

[ ] I was unable or did not want to participate in the recommended sexually transmitted infection (gonorrhea, chlamydia, and syphilis) testing during clinic visits for PrEP care

[ ] Another reason, please specify:: _________________________________________________*

[ ] Prefer not to answer

[ ] Don't know

**Page entry logic:** This page will show when: #44 Question "**Are you currently taking PrEP?**" is one of the following answers ("Yes")

## Current PrEP Flag

**Page entry logic:** This page will show when: ((#50 Question "**How many months in a row have you been taking Apretude?**" is one of the following answers ("7-12 months","More than 12 months") OR #51 Question "**How many months in a row have you been taking Truvada?**" is one of the following answers ("7-12 months","More than 12 months")) OR #52 Question "**How many months in a row have you been taking Descovy?**" is one of the following answers ("7-12 months","More than 12 months"))

## Longer than 7 months PrEP flag

**Page entry logic:** This page will show when: ((#44 Question "**Are you currently taking PrEP?**" is one of the following answers ("Yes") OR #53 Question "**In the past 6 months (since [question('value'), id='3']), have you taken PrEP?**" is one of the following answers ("Yes")) AND #22 Question "**Have you had an HIV test the past 6 months (since [question('value'), id='3'])?**" is one of the following answers ("Yes"))

## PrEP Check - HIV testing

**Page entry logic:** This page will show when: ((#44 Question "**Are you currently taking PrEP?**" is one of the following answers ("Yes") OR #53 Question "**In the past 6 months (since [question('value'), id='3']), have you taken PrEP?**" is one of the following answers ("Yes")) AND #35 Question "**In the past 6 months (since [question('value'), id='3']), were you tested for the sexually transmitted infections gonorrhea, chlamydia, or syphilis?**" is one of the following answers ("Yes"))

## PrEP Check - STI testing

**Page entry logic:** This page will show when: ((#50 Question "**How many months in a row have you been taking Apretude?**" is one of the following answers ("Less than 2 months","2-6 months","7-12 months") OR #51 Question "**How many months in a row have you been taking Truvada?**" is one of the following answers ("Less than 2 months","2-6 months","7-12 months")) OR #52 Question "**How many months in a row have you been taking Descovy?**" is one of the following answers ("Less than 2 months","2-6 months","7-12 months"))

## PrEP Module - PART 2

**Logic: Show/hide trigger exists.**

Shortname / Alias: p6m_other_prep

#### ****In the past 6 months (since [question('value'), id='3']) have you taken any other PrEP medication?****

( ) Yes

( ) No

( ) Prefer not to answer

( ) Don't know

**Logic: Show/hide trigger exists. Hidden unless: #56 Question "In the past 6 months (since [question('value'), id='3']) have you taken any other PrEP medication?" is one of the following answers ("Yes")**

Shortname / Alias: p6m_prep_other_

#### ****In the past 6 months, which other PrEP prescription medications did you take? Choose all that apply.****

[ ] Truvada (name brand or generic)

[ ] Descovy

[ ] Apretude

[ ] Prefer not to answer

[ ] Don't know

**Logic: Hidden unless: #57 Question "In the past 6 months, which other PrEP prescription medications did you take? Choose all that apply." is one of the following answers ("Apretude")**

Shortname / Alias: prevprep_apretude_location

#### ****Where did you go to get your Apretude injections?****

( ) Private doctor's office

( ) STI Clinic

( ) Health Center

( ) Pharmacy

( ) At home

( ) Other, please specify:: _________________________________________________*

( ) Prefer not to answer

( ) Don't know

**Page entry logic:** This page will show when: #56 Question "**In the past 6 months (since [question('value'), id='3']) have you taken any other PrEP medication?**" is one of the following answers ("Yes")

## PrEP Module - PART 3

Shortname / Alias: prep_change_reas_

#### ****Which of the following describes the reason(s) why you changed the PrEP medication you were using? Choose all that apply.****

[ ] I couldn’t afford it

[ ] I just wanted to try something different

[ ] I experienced or was concerned about side effects

[ ] I found the injection to be painful or uncomfortable

[ ] I found it difficult to keep my injection appointments

[ ] I could not remember to take the pills

[ ] I was worried that people will think that I have HIV when they see me taking the pill or going to clinic for the injection

[ ] I was worried people will think I am very sexually active because I am on PrEP

[ ] I was worried about my privacy with someone that I live with

[ ] I was worried about my privacy on my parent’s or partner’s health insurance plan

[ ] Another reason, please specify:: _________________________________________________*

## PrEP Opinion

### ****Please indicate how much you agree or disagree with the following statements.****

Shortname / Alias: prep_opinion_gay

#### ****Taking PrEP is a sign of being gay.****

( ) Disagree

( ) Somewhat disagree

( ) Neutral

( ) Somewhat agree

( ) Agree

( ) Don't know

Shortname / Alias: prep_opinion_top

#### ****I do not need PrEP if I am a "top".****

( ) Disagree

( ) Somewhat disagree

( ) Neutral

( ) Somewhat agree

( ) Agree

( ) Don't know

Shortname / Alias: prep_opinion_partners

#### ****I would feel comfortable telling my sexual partners if I take PrEP.****

( ) Disagree

( ) Somewhat disagree

( ) Neutral

( ) Somewhat agree

( ) Agree

( ) Don't know

Shortname / Alias: prep_opinion_providers

#### ****I would feel comfortable telling my medical providers if I take PrEP.****

( ) Disagree

( ) Somewhat disagree

( ) Neutral

( ) Somewhat agree

( ) Agree

( ) Don't know

Shortname / Alias: prep_opinion_friends

#### ****I would feel comfortable telling my friends if I used PrEP.****

( ) Disagree

( ) Somewhat disagree

( ) Neutral

( ) Somewhat agree

( ) Agree

( ) Don't know

## PrEP Adherence Self-Efficacy Scale

### ****We are going to ask you about situations that could occur while taking PrEP. For the following questions we will ask you to tell us how confident you are that you could to do the following things.****

### ****Use this response scale ranging from 0 ("could not do at all") to 10 ("certainly could do").****

Shortname / Alias: prep_scale_

### ****How confident are you that you could:****

Validation: Min = 0 Max = 10

Shortname / Alias: prep_scale_1

#### Stick to taking PrEP even when side effects begin to interfere with daily activities? Common side effects can include headache, nausea, stomach pain, and fatigue.

0 ________________________[__]_____________________________ 10

Validation: Min = 0 Max = 10

Shortname / Alias: prep_scale_2

#### Integrate taking PrEP into your daily routine?

0 ________________________[__]_____________________________ 10

Validation: Min = 0 Max = 10

Shortname / Alias: prep_scale_3

#### Integrate taking PrEP into your daily routine even if it means taking medications or doing other things in front of people who don't know you are taking PrEP?

0 ________________________[__]_____________________________ 10

Validation: Min = 0 Max = 10

Shortname / Alias: prep_scale_4

#### Stick to your PrEP schedule even when your daily routine is disrupted?

0 ________________________[__]_____________________________ 10

Validation: Min = 0 Max = 10

Shortname / Alias: prep_scale_5

#### Stick to your PrEP schedule when you aren't feeling well?

0 ________________________[__]_____________________________ 10

Validation: Min = 0 Max = 10

Shortname / Alias: prep_scale_6

#### Continue taking PrEP even if it means doing so interferes with your daily activities?

0 ________________________[__]_____________________________ 10

Validation: Min = 0 Max = 10

Shortname / Alias: prep_scale_7

#### Continue taking PrEP even when you are feeling discouraged about your sexual health?

0 ________________________[__]_____________________________ 10

Validation: Min = 0 Max = 10

Shortname / Alias: prep_scale_8

#### Continue taking PrEP even when getting to your clinic appointments is a major hassle?

0 ________________________[__]_____________________________ 10

Validation: Min = 0 Max = 10

Shortname / Alias: prep_scale_9

#### Continue taking PrEP even if people close to you tell you that they don't think it is doing any good?

0 ________________________[__]_____________________________ 10

## PrEP Importance Scale

### ****In the next few questions, we’re going to ask about how important some activities are to you and how confident you are that you can do them. Please use the scale provided to answer each question to the best of your ability.****

Validation: Min = 0 Max = 10

Shortname / Alias: importance_hivannualtest

#### ****On a scale of 0 to 10, with 0 being not at all important and 10 being very important, how**** important ****is it for you to test for HIV at least once per year?****

0 ________________________[__]_____________________________ 10

Validation: Min = 0 Max = 10

Shortname / Alias: confidence_hivannualtest

#### ****On a scale of 0 to 10, with 0 being not at all confident and 10 being very confident, how**** confident ****are you to test for HIV at least once per year?****

0 ________________________[__]_____________________________ 10

Validation: Min = 0 Max = 10

Shortname / Alias: importance_stiannualtest

#### ****On a scale of 0 to 10, with 0 being not at all important and 10 being very important, how**** important ****is it for you to test for STIs at least once per year?****

0 ________________________[__]_____________________________ 10

Validation: Min = 0 Max = 10

Shortname / Alias: confidence_stiannualtest

#### ****On a scale of 0 to 10, with 0 being not at all confident and 10 being very confident, how**** confident ****are you to test for STIs at least once per year?****

0 ________________________[__]_____________________________ 10

Validation: Min = 0 Max = 10

Shortname / Alias: importance_startprep

#### ****On a scale of 0 to 10, with 0 being not at all important and 10 being very important, how**** important ****is it for you to start taking PrEP to prevent HIV?****

0 ________________________[__]_____________________________ 10

Validation: Min = 0 Max = 10

Shortname / Alias: confidence_startprep

#### ****On a scale of 0 to 10, with 0 being not at all confident and 10 being very confident, how**** confident ****are you to start taking PrEP to prevent HIV?****

0 ________________________[__]_____________________________ 10

**Page entry logic:** This page will show when: (arm is exactly equal to "2" OR arm is exactly equal to "3")

## Uptake of Self-Testing

### ****In this next section, we want to know more about your response to having free HIV/STI self-testing kits available to order.****

###

**Logic: Show/hide trigger exists.**

Shortname / Alias: order_self_kit

#### ****Have you ordered one of the free self-testing kits that we offer?****

( ) Yes

( ) No

( ) Prefer not to answer

( ) Don't know

**Logic: Hidden unless: #73 Question "Have you ordered one of the free self-testing kits that we offer?" is one of the following answers ("Yes")**

Shortname / Alias: self_kit_type_

#### ****Which type of self-test kit did you order? Select all that apply.****

[ ] HIV

[ ] STI

**Logic: Hidden unless: #73 Question "Have you ordered one of the free self-testing kits that we offer?" is one of the following answers ("Yes")**

Shortname / Alias: self_kit_order_reas

#### ****What was the main reason for your order?****

( ) I test regularly and it was time to test

( ) To understand the illness or symptoms that I have/had

( ) Advice from health care provider or pharmacist

( ) I recently had a possible exposure to HIV

( ) Encouraged by sex partner

( ) To see what it was like to use a self-test

( ) Other, please specify:: _________________________________________________*

( ) Prefer not to answer

( ) Don't know

**Logic: Show/hide trigger exists. Hidden unless: #73 Question "Have you ordered one of the free self-testing kits that we offer?" is one of the following answers ("Yes")**

Shortname / Alias: self_kit_

#### ****Which type of self-test kit did you order?**** The HIV kit is an OraQuick test, which is only an oral swab from inside your mouth. The STI kit would have asked for multiple collections, such as a urine sample, blood prick, rectal swab, and an oral swab.

[ ] HIV Kit (OraQuick)

[ ] STI Kit

[ ] Both

[ ] Prefer not to answer

[ ] Don't know

**Logic: Hidden unless: #76 Question "Which type of self-test kit did you order?
The HIV kit is an OraQuick test, which is only an oral swab from inside your mouth. The STI kit would have asked for multiple collections, such as a urine sample, blood prick, rectal swab, and an oral swab." is one of the following answers ("HIV Kit (OraQuick)","Both")**

Shortname / Alias: hiv_kit_time

#### ****How long did it take to complete the HIV kit in its entirety?**** By entirety, we mean reading the instructions, collecting the sample, and waiting for the results.

( ) I have not used the kit yet

( ) Less than 10 min

( ) 10 - 30 min

( ) 31 - 60 min

( ) 61 - 90 min

( ) 91 - 120 min

( ) More than 120 min

( ) Prefer not to answer

( ) Don't know

**Logic: Hidden unless: #76 Question "Which type of self-test kit did you order?
The HIV kit is an OraQuick test, which is only an oral swab from inside your mouth. The STI kit would have asked for multiple collections, such as a urine sample, blood prick, rectal swab, and an oral swab." is one of the following answers ("STI Kit","Both")**

Shortname / Alias: sti_kit_time

#### ****How long did it take to complete the STI kit in its entirety?**** By entirety, we mean reading the instructions, collecting the sample, and dropping the samples off for processing.

( ) I have not used the kit yet

( ) Less than 10 min

( ) 10 - 30 min

( ) 31 - 60 min

( ) 61 - 90 min

( ) 91 - 120 min

( ) More than 120 min

( ) Prefer not to answer

( ) Don't know

**Logic: Hidden unless: #73 Question "Have you ordered one of the free self-testing kits that we offer?" is one of the following answers ("No")**

Shortname / Alias: self_kit_no_order

#### ****Which of these best describes why you have not ordered an HIV or STI self-testing kit?****

( ) I am at low risk for HIV/STI infection

( ) I was afraid of finding out that I have HIV/an STI

( ) Didn't have time

( ) Prefer to test at a healthcare provider's office

( ) Other, please specify:: _________________________________________________*

( ) No particular reason

( ) Prefer not to answer

( ) Don't know

**Page entry logic:** This page will show when: (arm is exactly equal to "1" OR arm is exactly equal to "3")

## Motivational Interview Feedback - PART 1

### ****In this next section, we would like your feedback on the motivational interview (MI) that you took part in at the beginning of the study.****

**Logic: Show/hide trigger exists.**

Shortname / Alias: mi_remember

#### ****Do you remember participating in a discussion with a member of the research team at the beginning of the study to review the app and make a plan to use its features?****

( ) Yes

( ) No

( ) I was not able to schedule the call with staff

( ) I did not want to participant in the call with staff

( ) Don't know

**Logic: Hidden unless: #80 Question "Do you remember participating in a discussion with a member of the research team at the beginning of the study to review the app and make a plan to use its features?" is one of the following answers ("Yes")**

Shortname / Alias: mi_challenge_

#### ****Despite the self-scheduling aspect, we understand there are several factors that may have made it challenging to schedule the discussion. Did you encounter any of the following while scheduling your discussion?****

[ ] Had to alter work schedule (work from home, take day off, take a longer lunch break, etc.)

[ ] Lost (did not receive) any wages, earnings, gifts, or in-kind reimbursement for services

[ ] Had to arrange for anyone in your household (children and/or adults) to be cared for

[ ] Other, please specify:: _________________________________________________*

[ ] Prefer not to answer

[ ] Don't know

**Logic: Hidden unless: #80 Question "Do you remember participating in a discussion with a member of the research team at the beginning of the study to review the app and make a plan to use its features?" is one of the following answers ("Yes")**

Shortname / Alias: mi_app_explore

#### ****Was it helpful to explore the app during the discussion?****

( ) Yes

( ) No

( ) Prefer not to answer

( ) Don't know

**Logic: Show/hide trigger exists. Hidden unless: #80 Question "Do you remember participating in a discussion with a member of the research team at the beginning of the study to review the app and make a plan to use its features?" is one of the following answers ("Yes")**

Shortname / Alias: mi_app_discuss

#### ****Is there anything about the app that you wish you had been able to discuss with the research team member?****

( ) Yes

( ) No

( ) Prefer not to answer

( ) Don't know

**Logic: Hidden unless: #83 Question "Is there anything about the app that you wish you had been able to discuss with the research team member?" is one of the following answers ("Yes")**

Shortname / Alias: mi_app_discuss_topic

### ****What app-related topic do you wish had been discussed?****

_________________________________________________

**Logic: Hidden unless: #80 Question "Do you remember participating in a discussion with a member of the research team at the beginning of the study to review the app and make a plan to use its features?" is one of the following answers ("Yes")**

Shortname / Alias: mi_discuss_hiv_comfort

#### ****Were you comfortable discussing your sexual health and experiences with HIV prevention with the research team member?****

( ) Yes

( ) No

( ) Prefer not to answer

( ) Don't know

**Page entry logic:** This page will show when: #80 Question "**Do you remember participating in a discussion with a member of the research team at the beginning of the study to review the app and make a plan to use its features?**" is one of the following answers ("Yes")

## Motivational Interview Feedback - PART 2

Shortname / Alias: mi_prior_knowledge

#### ****Prior to the discussion, did you know that certain behaviors (such as condomless sex without PrEP adherence, recent STI diagnosis, and engaging in sex while drunk or high) can increase your risk for getting HIV?****

( ) Yes

( ) No

( ) Prefer not to answer

( ) Don't know

Shortname / Alias: mi_prev_strat_

#### ****What prevention strategies have you used since the discussion to help protect yourself from HIV or other STIs? Choose all that apply.****

[ ] Regular HIV/STI Testing (e.g., at least once per year)

[ ] PrEP

[ ] PEP

[ ] Condoms

[ ] None of the above

[ ] Prefer not to answer

[ ] Don't know

Shortname / Alias: barrier_

#### ****Which of the following barriers would you identify that make it challenging to protect your sexual health? Choose all that apply.****

[ ] Transportation

[ ] Cost, affordability

[ ] Not knowing where to get HIV/STI test

[ ] Forgetting to test

[ ] Not knowing about the need for regular HIV testing

[ ] Not knowing about PrEP/PEP or how to take/get PrEP/PEP

[ ] Fear or experience of stigma or discrimination

[ ] None of the above

[ ] Prefer not to answer

[ ] Don't know

Shortname / Alias: discussion_address

#### ****Did the discussion help you address or work through one or more barriers that you identified?****

( ) Yes

( ) No

( ) Prefer not to answer

( ) Don't know

**Logic: Show/hide trigger exists.**

Shortname / Alias: action_plan_ft

#### ****Have you been able to follow through with your action plan since the discussion?****

( ) Yes

( ) No

( ) Prefer not to answer

( ) Don't know

**Logic: Hidden unless: #90 Question "Have you been able to follow through with your action plan since the discussion?" is one of the following answers ("No")**

Shortname / Alias: prevent_plan_

#### ****Can you explain why or what factors have prevented you from following through with your action plan?****

[ ] Not needed to do anything from my action plan yet (e.g., plan on testing every year and it is not time to test again yet)

[ ] Transportation

[ ] Cost, affordability

[ ] Not knowing where to get HIV/STI test

[ ] Forgetting to test

[ ] Not knowing about the need for regular HIV testing

[ ] Not knowing about PrEP/PEP or how to take/get PrEP/PEP

[ ] Fear or experience of stigma or discrimination

[ ] Other, please specify:: _________________________________________________*

[ ] None of the above

[ ] Prefer not to answer

[ ] Don't know

Shortname / Alias: action_plan_goals

#### ****Was creating an action plan helpful to achieving your sexual health goals?****

( ) Yes

( ) No

( ) Prefer not to answer

( ) Don't know

Validation: Min = 0 Max = 10

Shortname / Alias: action_plan_stick

#### ****On a scale from 0 to 10, with 0 being not confident at all and 10 being extremely confident, how confident are you in continuing to stick to your action plan?****

0 ________________________[__]_____________________________ 10

## App Features

Shortname / Alias: best_app_feature

#### ****What has been the most useful app feature or the feature you've liked the most?****

( ) Quizzes

( ) Provider Locations

( ) Reminders

( ) Ordering Features

( ) HIV/STI Information

( ) Other, please specify:: _________________________________________________*

( ) Don't know

( ) None of the above

## Systems Usability Scale

###

Shortname / Alias: sus

#### ****Please indicate how much you agree or disagree with the following statements.****

|  | **Strongly disagree** | **Disagree** | **Neutral** | **Agree** | **Strongly agree** |
| --- | --- | --- | --- | --- | --- |
| **I think that I would like to use the Combine study app frequently.** | ( ) | ( ) | ( ) | ( ) | ( ) |
| **I found the Combine study app unnecessarily complex.** | ( ) | ( ) | ( ) | ( ) | ( ) |
| **I thought the Combine study app was easy to use.** | ( ) | ( ) | ( ) | ( ) | ( ) |
| **I think that I would need the support of a technical person to be able to use the Combine study app.** | ( ) | ( ) | ( ) | ( ) | ( ) |
| **I found the various functions in the Combine study app to be well integrated.** | ( ) | ( ) | ( ) | ( ) | ( ) |
| **I thought there was too much inconsistency in the Combine study app.** | ( ) | ( ) | ( ) | ( ) | ( ) |
| **I would imagine that most people would learn to use the Combine study app very quickly.** | ( ) | ( ) | ( ) | ( ) | ( ) |
| **I found the Combine study app very cumbersome to use.** | ( ) | ( ) | ( ) | ( ) | ( ) |
| **I felt very confident using the Combine study app.** | ( ) | ( ) | ( ) | ( ) | ( ) |
| **I needed to learn a lot of things before I could get going with the Combine study app.** | ( ) | ( ) | ( ) | ( ) | ( ) |

## Ordering

**Logic: Show/hide trigger exists. Hidden unless: (arm is exactly equal to "2" OR arm is exactly equal to "3")**

Shortname / Alias: order_clkit_23

#### ****Have you ordered condoms, lube, HIV test kits, or STI test kits through the Combine study app?****

( ) Yes

( ) No

( ) Prefer not to answer

( ) Don't know

**Logic: Show/hide trigger exists. Hidden unless: (arm is exactly equal to "0" OR arm is exactly equal to "1")**

Shortname / Alias: order_cl_01

#### ****Have you ordered condoms or lube through the Combine study app?****

( ) Yes

( ) No

( ) Prefer not to answer

( ) Don't know

**Logic: Hidden unless: (#96 Question "Have you ordered condoms, lube, HIV test kits, or STI test kits through the Combine study app?" is one of the following answers ("Yes") OR #97 Question "Have you ordered condoms or lube through the Combine study app?" is one of the following answers ("Yes"))**

Shortname / Alias: order_difficult

#### ****Did you have any difficulty placing an order?****

( ) Yes, please specify:: _________________________________________________*

( ) No

( ) Prefer not to answer

( ) Don't know

**Logic: Hidden unless: (#96 Question "Have you ordered condoms, lube, HIV test kits, or STI test kits through the Combine study app?" is one of the following answers ("Yes") OR #97 Question "Have you ordered condoms or lube through the Combine study app?" is one of the following answers ("Yes"))**

Shortname / Alias: order_motivate_

#### ****What were your motivations for ordering? Please select all that apply.****

[ ] Prompted by quiz in Combine study app

[ ] Exploring ordering section in Combine study app

[ ] Reminder in the Combine study app prompted me to order

[ ] I needed condoms, lube, or test kits and the app was a convenient place to get them

[ ] I needed condoms or lube and the app was a convenient place to get them

[ ] I made a plan after my discussion with study staff about how to use the app

[ ] Other, please specify:: _________________________________________________*

**Logic: Hidden unless: #96 Question "Have you ordered condoms, lube, HIV test kits, or STI test kits through the Combine study app?" is one of the following answers ("No")**

Shortname / Alias: noorderreas_clkit_23_

#### ****What were your reasons for not ordering condoms, lube, HIV test kits, or STI test kits?****

[ ] Not interested

[ ] Concerns about privacy

[ ] App was too difficult to use

[ ] I was not aware that these products were available for ordering

[ ] Other, please specify:: _________________________________________________*

**Logic: Hidden unless: #97 Question "Have you ordered condoms or lube through the Combine study app?" is one of the following answers ("No")**

Shortname / Alias: noorderreas_cl_01_

#### ****What were your reasons for not ordering condoms or lube?****

[ ] Not interested

[ ] Concerns about privacy

[ ] App was too difficult to use

[ ] I was not aware that these products were available for ordering

[ ] Other, please specify:: _________________________________________________*

## Quizzes

**Logic: Show/hide trigger exists.**

Shortname / Alias: usequiz_

#### ****Have you taken any of the quizzes in the app? Check all that apply.****

[ ] Condom Quiz

[ ] Mental Health

[ ] Paying for PrEP

[ ] PEP Screener

[ ] PrEP Screener

[ ] Substance Use

[ ] Testing Options/Preferences

[ ] None of the above

**Logic: Hidden unless: #102 Question "Have you taken any of the quizzes in the app? Check all that apply." is one of the following answers ("Condom Quiz","Mental Health","Paying for PrEP","PEP Screener","PrEP Screener","Substance Use","Testing Options/Preferences")**

Shortname / Alias: quiz_difficult

#### ****Did you have any difficulty using the quizzes in the app?****

( ) Yes, please specify:: _________________________________________________*

( ) No

( ) Prefer not to answer

( ) Don't know

**Logic: Hidden unless: #102 Question "Have you taken any of the quizzes in the app? Check all that apply." is one of the following answers ("Condom Quiz","Mental Health","Paying for PrEP","PEP Screener","PrEP Screener","Substance Use","Testing Options/Preferences")**

Shortname / Alias: quiz_motivation_

#### ****What were your motivations for taking a quiz? Check all that apply.****

[ ] Wanted to try it out

[ ] Reminder in the Combine study app prompted me

[ ] Linked to a quiz from a different section in the app

[ ] Exploring quiz section in Combine study app

[ ] I made a plan after my discussion with study staff about how to use the app

[ ] Other, please specify:: _________________________________________________*

**Logic: Hidden unless: #102 Question "Have you taken any of the quizzes in the app? Check all that apply." is one of the following answers ("Condom Quiz","Mental Health","Paying for PrEP","PEP Screener","PrEP Screener","Substance Use","Testing Options/Preferences")**

Shortname / Alias: quiz_result_help

#### ****Were the results of the quizzes you took helpful?****

( ) Yes

( ) No

( ) Prefer not to answer

( ) Don't know

**Logic: Hidden unless: #102 Question "Have you taken any of the quizzes in the app? Check all that apply." is exactly equal to ("None of the above")**

Shortname / Alias: noquiz_reas_

#### ****What were your reasons for not using any quizzes? Check all that apply.****

[ ] Not interested

[ ] Concerns about privacy

[ ] App was too difficult to use

[ ] I was not aware that the app offered quizzes

[ ] Other, please specify:: _________________________________________________*

## Locators

**Logic: Show/hide trigger exists.**

Shortname / Alias: locator_

#### ****Have you used any of the locators in the app? Check all that apply.****

[ ] PrEP Locator

[ ] Services Locator

[ ] Behavioral Health Locator

[ ] HIV Services Locator

[ ] None of the above

**Logic: Hidden unless: #107 Question "Have you used any of the locators in the app? Check all that apply." is one of the following answers ("PrEP Locator","Services Locator","Behavioral Health Locator","HIV Services Locator")**

Shortname / Alias: locator_difficulty

#### ****Did you have any difficulty using the locators in the app? (i.e., technical difficulties or lack of providers in area, etc.)****

( ) Yes, please specify:: _________________________________________________*

( ) No

( ) Prefer not to answer

( ) Don't know

**Logic: Hidden unless: #107 Question "Have you used any of the locators in the app? Check all that apply." is one of the following answers ("PrEP Locator","Services Locator","Behavioral Health Locator","HIV Services Locator")**

Shortname / Alias: locator_motivate_

#### ****What were your motivations for using a locator? Check all that apply.****

[ ] Reminder in the Combine study app prompted me

[ ] Linked to a locator from a quiz in the app

[ ] Linked to a locator from a different section in the app

[ ] Exploring the different features of the app

[ ] I made a plan after my discussion with study staff about how to use the app

[ ] Other, please specify:: _________________________________________________*

**Logic: Hidden unless: #107 Question "Have you used any of the locators in the app? Check all that apply." is one of the following answers ("PrEP Locator","Services Locator","Behavioral Health Locator","HIV Services Locator")**

Shortname / Alias: locator_helpful

#### ****Were the locators helpful?****

( ) Yes

( ) No

( ) Prefer not to answer

( ) Don't know

**Logic: Hidden unless: #107 Question "Have you used any of the locators in the app? Check all that apply." is one of the following answers ("None of the above")**

Shortname / Alias: nolocator_reas_

#### ****What were your reasons for not using the locators in the app? Check all that apply.****

[ ] Not interested

[ ] Concerns about privacy

[ ] App was too difficult to use

[ ] I was not aware that these locators were available in the app

[ ] Other, please specify:: _________________________________________________*

## Health Resources

**Logic: Show/hide trigger exists.**

Shortname / Alias: hlth_resource_

#### ****Have you viewed the health resources in the app? Please select all that apply.****

[ ] FAQs

[ ] HIV Information

[ ] STIs

[ ] PrEP

[ ] PEP

[ ] Condoms

[ ] Mental Health

[ ] Insurance

[ ] None of the above

**Logic: Hidden unless: #112 Question "Have you viewed the health resources in the app? Please select all that apply." is one of the following answers ("FAQs","HIV Information","STIs","PrEP","PEP","Condoms","Mental Health","Insurance")**

Shortname / Alias: difficulty_hlth_resource

#### ****Did you have any difficulty finding the health resources section in the app?****

( ) Yes

( ) No

( ) Prefer not to answer

( ) Don't know

**Logic: Hidden unless: #112 Question "Have you viewed the health resources in the app? Please select all that apply." is one of the following answers ("FAQs","HIV Information","STIs","PrEP","PEP","Condoms","Mental Health","Insurance")**

Shortname / Alias: motiv_hlthresource_

#### ****What were your motivations for viewing the health resources? Check all that apply.****

[ ] Reminder in the Combine study app prompted me

[ ] Linked to the resources from a different section in the app

[ ] Exploring the different features of the app

[ ] I made a plan after my discussion with study staff about how to use the app

[ ] Other, please specify:: _________________________________________________*

**Logic: Show/hide trigger exists. Hidden unless: #112 Question "Have you viewed the health resources in the app? Please select all that apply." is one of the following answers ("FAQs","HIV Information","STIs","PrEP","PEP","Condoms","Mental Health","Insurance")**

Shortname / Alias: helpful_hlthresource

#### ****Were the health resources helpful?****

( ) Yes

( ) No

( ) Prefer not to answer

( ) Don't know

**Logic: Hidden unless: #115 Question "Were the health resources helpful?" is one of the following answers ("No")**

Shortname / Alias: improve_hlthresource

### ****What could be done to improve the health resources section?****

____________________________________________

____________________________________________

____________________________________________

____________________________________________

**Logic: Hidden unless: #112 Question "Have you viewed the health resources in the app? Please select all that apply." is one of the following answers ("None of the above")**

Shortname / Alias: nohlth_resource_

#### ****What were your reasons for not using the health resources in the app? Check all that apply.****

[ ] Not interested

[ ] Concerns about privacy

[ ] App was too difficult to use

[ ] I was not aware that these health resources were available in the app

[ ] Other, please specify:: _________________________________________________*

**Page entry logic:** This page will show when: #115 Question "**Were the health resources helpful?**" is one of the following answers ("Yes")

## Health Resources - PART 2

Shortname / Alias: value_hlthresource

#### ****What was the most valuable section or topic?****

( ) FAQs

( ) HIV Information

( ) STIs

( ) PrEP

( ) PEP

( ) Condoms

( ) Mental Health

( ) Insurance

( ) No preference

## Preferences for Accessing App

Shortname / Alias: prefer_access

#### ****How would you prefer to access the information in the Combine study app?****

( ) Dedicated smartphone app like the Combine study app

( ) Website with login instead of app

( ) Other:: _________________________________________________*

( ) No preference

Shortname / Alias: trust_app_

#### ****Who would you trust to provide an app like the Combine study app? Check all that apply.****

[ ] Local health department

[ ] A community-based organization that offers HIV testing

[ ] A pharmacy

[ ] Other:: _________________________________________________*

[ ] No preference

Shortname / Alias: pref_healthcare_

#### ****How do you prefer to obtain your healthcare services? Check all that apply.****

[ ] Smartphone/apps/telehealth

[ ] In person

[ ] Other:: _________________________________________________*

[ ] No preference

## Sexual Behavior

### ****Next, we're going to ask you some questions about having sex. Please remember your answers will be kept private. While we acknowledge that people may have distinct names or ways of referring to their sexual body parts, we will be using standard terms because we need to be sure we are collecting the same information from everyone who takes the survey. It's important for the study that your answers be as accurate as possible. You may skip any questions you are not comfortable answering. Please let us know which questions are not applicable to you. We need to ask you all of these questions, even if some may not apply to your situation.**** Let's get started!

Shortname / Alias: sexlife_satis

#### ****How satisfied are you with your current sex life?****

( ) Very satisfied

( ) Satisfied

( ) Neutral

( ) Dissatisfied

( ) Very dissatisfied

( ) Prefer not to answer

( ) Don't know

## Sexual Behavior - Cis Male

### ****Please be aware of the time period we are asking about before you answer the question.****

**Logic: Show/hide trigger exists.**

Shortname / Alias: p6msex_men_

#### ****In the past 6 months (since [question('value'), id='3']), what types of sex have you had with cisgender men (assigned male sex at birth and identifies as male)?****

[ ] Oral sex (mouth on the penis)

[ ] Anal sex (penis in the butt)

[ ] I have not had any type of sex with a cisgender man in the past 6 months

Validation: Min = 1 Max = 999 Must be numeric Whole numbers only Positive numbers only Max character count = 3

**Logic: Show/hide trigger exists. Hidden unless: #123 Question "In the past 6 months (since [question('value'), id='3']), what types of sex have you had with cisgender men (assigned male sex at birth and identifies as male)?" is one of the following answers ("Oral sex (mouth on the penis)","Anal sex (penis in the butt)")**

Shortname / Alias: p6m_male_partnum

### ****During the last 6 months, with how many cisgender male partner(s) did you have anal or oral sex?*****

_________________________________________________

**Page entry logic:** This page will show when: (#123 Question "**In the past 6 months (since [question('value'), id='3']), what types of sex have you had with cisgender men (assigned male sex at birth and identifies as male)?**" is one of the following answers ("Oral sex (mouth on the penis)","Anal sex (penis in the butt)") AND #124 Question "**During the last 6 months, with how many cisgender male partner(s) did you have anal or oral sex?**" is greater than or equal to "1")

## Additional Questions - Cis Male Partners

**Logic: Hidden by default**

### ****The number of cisgender male partners without condom use you reported is greater than the total number of cisgender male partners you reported in the past 6 months. Please correct this.****

Validation: Min = 0 Max = 999 Must be numeric Whole numbers only Positive numbers only Max character count = 3

**Logic: Hidden unless: #124 Question "During the last 6 months, with how many cisgender male partner(s) did you have anal or oral sex?"**

Shortname / Alias: p6m_m_analsex

### ****Of the [question('value'), id='116'] cisgender male partner(s) you had sex with in the last 6 months (since [question('value'), id='3']), how many did you have anal sex with?****

_________________________________________________

Shortname / Alias: cism_exchange

#### ****Were any of the cisgender male sex partners an exchange partner - this is, a partner that you have sex with in exchange for money, drugs, food, or something else of value?****

( ) Yes

( ) No

( ) Prefer not to answer

( ) Don't know

**Page entry logic:** This page will show when: (#123 Question "**In the past 6 months (since [question('value'), id='3']), what types of sex have you had with cisgender men (assigned male sex at birth and identifies as male)?**" is one of the following answers ("Oral sex (mouth on the penis)","Anal sex (penis in the butt)") AND #124 Question "**During the last 6 months, with how many cisgender male partner(s) did you have anal or oral sex?**" is greater than or equal to "1")

## Cis Male Validation

## Sexual Behavior - Cis Female

### ****Please be aware of the time period we are asking about before you answer the question.****

**Logic: Show/hide trigger exists.**

Shortname / Alias: p6msex_women_

#### ****In the past 6 months (since [question('value'), id='3']), what types of sex have you had with cisgender women (assigned female sex at birth and identifies as female)?****

[ ] Oral sex (mouth on the penis)

[ ] Anal sex (penis in the butt)

[ ] Vaginal sex (penis in the vagina)

[ ] I have not had any type of sex with a cisgender woman in the past 6 months

Validation: Min = 1 Max = 999 Must be numeric Whole numbers only Positive numbers only Max character count = 3

**Logic: Show/hide trigger exists. Hidden unless: #127 Question "In the past 6 months (since [question('value'), id='3']), what types of sex have you had with cisgender women (assigned female sex at birth and identifies as female)?" is one of the following answers ("Anal sex (penis in the butt)","Vaginal sex (penis in the vagina)")**

Shortname / Alias: p6m_female_partnum

### ****During the last 6 months, with how many cisgender female partner(s) did you have vaginal or anal sex?****

_________________________________________________

**Page entry logic:** This page will show when: (#127 Question "**In the past 6 months (since [question('value'), id='3']), what types of sex have you had with cisgender women (assigned female sex at birth and identifies as female)?**" is one of the following answers ("Anal sex (penis in the butt)","Vaginal sex (penis in the vagina)") AND #128 Question "**During the last 6 months, with how many cisgender female partner(s) did you have vaginal or anal sex?**" is greater than or equal to "1")

## Additional Questions - Cis Female Partners

**Logic: Hidden by default**

### ****The number of cisgender female partners without condom use you reported is greater than the total number of cisgender female partners you reported in the past 6 months. Please correct this.****

Validation: Min = 0 Max = 999 Must be numeric Whole numbers only Positive numbers only Max character count = 3

**Logic: Hidden unless: #128 Question "During the last 6 months, with how many cisgender female partner(s) did you have vaginal or anal sex?"**

Shortname / Alias: p6m_fm_analsex

### ****Of the [question('value'), id='119'] cisgender female partner(s) you had sex with in the last 6 months (since [question('value'), id='3']), how many did you have vaginal or anal sex with without using a condom?****

_________________________________________________

**Logic: Hidden unless: #128 Question "During the last 6 months, with how many cisgender female partner(s) did you have vaginal or anal sex?"**

Shortname / Alias: p6m_fm_anal_sex_nocondom

#### ****When you had sex with your cisgender female partner(s) in the last 6 months (since [question('value'), id='3']), did you ever have vaginal or anal sex without a condom?****

( ) Yes

( ) No

( ) Prefer not to answer

( ) Don't know

Shortname / Alias: cisf_exchange

#### ****Were any of these cisgender female sex partners an exchange partner - that is, a partner that you have sex with in exchange for money, drugs, food, or something else of value?****

( ) Yes

( ) No

( ) Prefer not to answer

( ) Don't know

**Page entry logic:** This page will show when: (#127 Question "**In the past 6 months (since [question('value'), id='3']), what types of sex have you had with cisgender women (assigned female sex at birth and identifies as female)?**" is one of the following answers ("Anal sex (penis in the butt)","Vaginal sex (penis in the vagina)") AND #128 Question "**During the last 6 months, with how many cisgender female partner(s) did you have vaginal or anal sex?**" is greater than or equal to "1")

## Cis Female Validation

## Sexual Behavior - Trans Female

### ****Please be aware of the time period we are asking about before you answer the question.****

**Logic: Show/hide trigger exists.**

Shortname / Alias: p6msex_twomen_

#### ****In the past 6 months (since [question('value'), id='3']), what types of sex have you had with transgender female or transfeminine people (assigned male sex at birth and identifies as transfeminine or female)?****

[ ] Oral sex (mouth on the penis)

[ ] Anal sex (penis in the butt)

[ ] Vaginal sex (penis in the vagina)

[ ] I have not had any type of sex with a transgender woman in the past 6 months

Validation: Min = 1 Max = 999 Must be numeric Whole numbers only Positive numbers only Max character count = 3

**Logic: Show/hide trigger exists. Hidden unless: #132 Question "In the past 6 months (since [question('value'), id='3']), what types of sex have you had with transgender female or transfeminine people (assigned male sex at birth and identifies as transfeminine or female)?" is one of the following answers ("Anal sex (penis in the butt)","Vaginal sex (penis in the vagina)")**

Shortname / Alias: p6m_tfemale_partnum

### ****During the last 6 months, with how many trans feminine individuals did you have vaginal or anal sex?****

_________________________________________________

**Page entry logic:** This page will show when: (#132 Question "**In the past 6 months (since [question('value'), id='3']), what types of sex have you had with transgender female or transfeminine people (assigned male sex at birth and identifies as transfeminine or female)?**" is one of the following answers ("Anal sex (penis in the butt)","Vaginal sex (penis in the vagina)") AND #133 Question "**During the last 6 months, with how many trans feminine individuals did you have vaginal or anal sex?**" is greater than or equal to "1")

## Additional Questions - Trans Female

**Logic: Hidden by default**

### ****The number of trans feminine partners without condom use you reported is greater than the total number of trans feminine partners you reported in the past 6 months. Please correct this.****

Validation: Min = 0 Max = 999 Must be numeric Whole numbers only Positive numbers only Max character count = 3

**Logic: Hidden unless: #133 Question "During the last 6 months, with how many trans feminine individuals did you have vaginal or anal sex?"**

Shortname / Alias: p6m_tfm_analsex

### ****Of the [question('value'), id='120'] trans feminine partner(s) you had sex with in the last 6 months (since [question('value'), id='3']), how many did you have vaginal or anal sex with without using a condom?****

_________________________________________________

**Logic: Hidden unless: #133 Question "During the last 6 months, with how many trans feminine individuals did you have vaginal or anal sex?"**

Shortname / Alias: p6m_tf_anal_sex_nocondom

#### ****When you had sex with your trans feminine partner(s) in the last 6 months (since [question('value'), id='3']), did you ever have vaginal or anal sex without a condom?****

( ) Yes

( ) No

( ) Prefer not to answer

( ) Don't know

Shortname / Alias: transf_exchange

#### ****Were any of these trans feminine individuals an exchange partner - that is, a partner that you have sex with in exchange for money, drugs, food, or something else of value?****

( ) Yes

( ) No

( ) Prefer not to answer

( ) Don't know

**Page entry logic:** This page will show when: (#132 Question "**In the past 6 months (since [question('value'), id='3']), what types of sex have you had with transgender female or transfeminine people (assigned male sex at birth and identifies as transfeminine or female)?**" is one of the following answers ("Anal sex (penis in the butt)","Vaginal sex (penis in the vagina)") AND #133 Question "**During the last 6 months, with how many trans feminine individuals did you have vaginal or anal sex?**" is greater than or equal to "1")

## Trans Female Validation

## Sexual Behavior - Trans Male

### ****Please be aware of the time period we are asking about before you answer the question.****

**Logic: Show/hide trigger exists.**

Shortname / Alias: p6msex_tmen_

#### ****In the past 6 months (since [question('value'), id='3']), what types of sex have you had with transgender male or transmasculine people (assigned female sex at birth and identifies as transmasculine or male)?****

[ ] Oral sex (mouth on the penis)

[ ] Anal sex (penis in the butt)

[ ] Vaginal sex (penis in the vagina)

[ ] I have not had any type of sex with a transgender man in the past 6 months

Validation: Min = 1 Max = 999 Must be numeric Whole numbers only Positive numbers only Max character count = 3

**Logic: Show/hide trigger exists. Hidden unless: #137 Question "In the past 6 months (since [question('value'), id='3']), what types of sex have you had with transgender male or transmasculine people (assigned female sex at birth and identifies as transmasculine or male)?" is one of the following answers ("Anal sex (penis in the butt)","Vaginal sex (penis in the vagina)")**

Shortname / Alias: p6m_tmale_partnum

### ****During the last 6 months, with how many trans masculine individuals did you have penetrative sex?****

_________________________________________________

**Page entry logic:** This page will show when: (#137 Question "**In the past 6 months (since [question('value'), id='3']), what types of sex have you had with transgender male or transmasculine people (assigned female sex at birth and identifies as transmasculine or male)?**" is one of the following answers ("Anal sex (penis in the butt)","Vaginal sex (penis in the vagina)") AND #138 Question "**During the last 6 months, with how many trans masculine individuals did you have penetrative sex?**" is greater than or equal to "1")

## Additional Questions - Trans Male

**Logic: Hidden by default**

### ****The number of trans masculine partners without condom use you reported is greater than the total number of trans masculine partners you reported in the past 6 months. Please correct this.****

Validation: Min = 0 Max = 999 Must be numeric Whole numbers only Positive numbers only Max character count = 3

**Logic: Hidden unless: #138 Question "During the last 6 months, with how many trans masculine individuals did you have penetrative sex?"**

Shortname / Alias: p6m_tm_analsex

### ****Of the [question('value'), id='121'] trans masculine partner(s) you had sex with in the last 6 months (since [question('value'), id='3']), how many did you have penetrative sex with without using a condom?****

_________________________________________________

**Logic: Hidden unless: #138 Question "During the last 6 months, with how many trans masculine individuals did you have penetrative sex?"**

Shortname / Alias: p6m_tm_anal_sex_nocondom

#### ****When you had sex with your trans masculine partner(s) in the last 6 months (since [question('value'), id='3']), did you ever have vaginal or anal sex without a condom?****

( ) Yes

( ) No

( ) Prefer not to answer

( ) Don't know

Shortname / Alias: transm_exchange

#### ****Were any of these trans masculine individuals an exchange partner - that is, a partner that you have sex with in exchange for money, drugs, food, or something else of value?****

( ) Yes

( ) No

( ) Prefer not to answer

( ) Don't know

**Page entry logic:** This page will show when: (#137 Question "**In the past 6 months (since [question('value'), id='3']), what types of sex have you had with transgender male or transmasculine people (assigned female sex at birth and identifies as transmasculine or male)?**" is one of the following answers ("Anal sex (penis in the butt)","Vaginal sex (penis in the vagina)") AND #138 Question "**During the last 6 months, with how many trans masculine individuals did you have penetrative sex?**" is greater than or equal to "1")

## Trans Male Validation

## Sexual Behavior - Non-Binary

### ****Please be aware of the time period we are asking about before you answer the question.****

**Logic: Show/hide trigger exists.**

Shortname / Alias: p6msex_nonbin_

#### ****In the past 6 months (since [question('value'), id='3']), what types of sex have you had with non-binary/gender non-conforming/genderqueer/two-spirit people?****

[ ] Oral sex (mouth on the penis)

[ ] Anal sex (penis in the butt)

[ ] Vaginal sex (penis in the vagina)

[ ] I have not had any type of sex with a non-binary/gender non-conforming/genderqueer/two-spirit person in the past 6 months

Validation: Min = 1 Max = 999 Must be numeric Whole numbers only Positive numbers only Max character count = 3

**Logic: Show/hide trigger exists. Hidden unless: #142 Question "In the past 6 months (since [question('value'), id='3']), what types of sex have you had with non-binary/gender non-conforming/genderqueer/two-spirit people?" is one of the following answers ("Anal sex (penis in the butt)","Vaginal sex (penis in the vagina)")**

Shortname / Alias: p6m_nonbin_partnum

### ****During the last 6 months, with how many non-binary/genderqueer/two-spirit individuals did you have penetrative sex?****

_________________________________________________

**Page entry logic:** This page will show when: (#142 Question "**In the past 6 months (since [question('value'), id='3']), what types of sex have you had with non-binary/gender non-conforming/genderqueer/two-spirit people?**" is one of the following answers ("Anal sex (penis in the butt)","Vaginal sex (penis in the vagina)") AND #143 Question "**During the last 6 months, with how many non-binary/genderqueer/two-spirit individuals did you have penetrative sex?**" is greater than or equal to "1")

## Additional Questions - Non-Binary

**Logic: Hidden by default**

### ****The number of non-binary/genderqueer/two-spirit partners without condom use you reported is greater than the total number of non-binary/genderqueer/two-spirit partners you reported in the past 6 months. Please correct this.****

Validation: Min = 0 Max = 999 Must be numeric Whole numbers only Positive numbers only Max character count = 3

**Logic: Hidden unless: #143 Question "During the last 6 months, with how many non-binary/genderqueer/two-spirit individuals did you have penetrative sex?"**

Shortname / Alias: p6m_nb_analsex

### ****Of the [question('value'), id='122'] non-binary/genderqueer/two-spirit partner(s) you had sex with in the last 6 months (since [question('value'), id='3']), how many did you have penetrative sex with without using a condom?****

_________________________________________________

**Logic: Hidden unless: #143 Question "During the last 6 months, with how many non-binary/genderqueer/two-spirit individuals did you have penetrative sex?"**

Shortname / Alias: p6m_nb_anal_sex_nocondom

#### ****When you had sex with your non-binary/genderqueer/two-spirit partner(s) in the last 6 months (since [question('value'), id='3']), did you ever have vaginal or anal sex without a condom?****

( ) Yes

( ) No

( ) Prefer not to answer

( ) Don't know

Shortname / Alias: nb_exchange

#### ****Were any of these non-binary/genderqueer/two-spirit individuals an exchange partner - that is, a partner that you have sex with in exchange for money, drugs, food, or something else of value?****

( ) Yes

( ) No

( ) Prefer not to answer

( ) Don't know

**Page entry logic:** This page will show when: (#142 Question "**In the past 6 months (since [question('value'), id='3']), what types of sex have you had with non-binary/gender non-conforming/genderqueer/two-spirit people?**" is one of the following answers ("Anal sex (penis in the butt)","Vaginal sex (penis in the vagina)") AND #143 Question "**During the last 6 months, with how many non-binary/genderqueer/two-spirit individuals did you have penetrative sex?**" is greater than or equal to "1")

## Non-Binary Validation

## Additional sex questions - Exchange

**Logic: Hidden unless: ((((#126 Question "Were any of the cisgender male sex partners an exchange partner - this is, a partner that you have sex with in exchange for money, drugs, food, or something else of value?" is one of the following answers ("Yes") OR #131 Question "Were any of these cisgender female sex partners an exchange partner - that is, a partner that you have sex with in exchange for money, drugs, food, or something else of value?" is one of the following answers ("Yes")) OR #136 Question "Were any of these trans feminine individuals an exchange partner - that is, a partner that you have sex with in exchange for money, drugs, food, or something else of value?" is one of the following answers ("Yes")) OR #141 Question "Were any of these trans masculine individuals an exchange partner - that is, a partner that you have sex with in exchange for money, drugs, food, or something else of value?" is one of the following answers ("Yes")) OR #146 Question "Were any of these non-binary/genderqueer/two-spirit individuals an exchange partner - that is, a partner that you have sex with in exchange for money, drugs, food, or something else of value?" is one of the following answers ("Yes"))**

Shortname / Alias: exchange_type

#### ****You said you exchanged sex for money, drugs, food, or something else of value. Do you receive goods/money, give goods/money or both?****

( ) I received goods and/or money

( ) I gave goods and/or money

( ) Both

( ) Prefer not to answer

( ) Don't know

## Partner calculating

**Page entry logic:** This page will show when: #124 Question "**During the last 6 months, with how many cisgender male partner(s) did you have anal or oral sex?**" is exactly equal to "1"

## 1 Cis Male Partner

Shortname / Alias: one_cism_main_part

#### ****Earlier you told us you have had one male partner in the past 6 months (since [question('value'), id='3']). Is/was this partner a main partner? A main partner is someone that you feel committed to above all others -- this is someone you might call your boyfriend, significant other, or life partner.****

( ) Yes

( ) No

( ) Prefer not to answer

( ) Don't know

Shortname / Alias: one_cism_ongoing

#### ****Next, think of whether you are currently in an active, ongoing sexual relationship with your male partner. By that, we mean that you generally have oral or anal sex at least once per month and you expect to continue doing so for some time. Is the relationship with this partner active and ongoing?****

( ) Yes

( ) No

( ) Prefer not to answer

( ) Don't know

**Logic: Hidden unless: (#149 Question "Next, think of whether you are currently in an active, ongoing sexual relationship with your male partner. By that, we mean that you generally have oral or anal sex at least once per month and you expect to continue doing so for some time. Is the relationship with this partner active and ongoing?" is one of the following answers ("No") AND #148 Question "Earlier you told us you have had one male partner in the past 6 months (since [question('value'), id='3']). Is/was this partner a main partner? A main partner is someone that you feel committed to above all others -- this is someone you might call your boyfriend, significant other, or life partner." is one of the following answers ("No"))**

Shortname / Alias: onepart_onetime

#### ****Was this a one-time partner?****

( ) Yes

( ) No

( ) Prefer not to answer

( ) Don't know

**Logic: Show/hide trigger exists. Hidden unless: ((#148 Question "Earlier you told us you have had one male partner in the past 6 months (since [question('value'), id='3']). Is/was this partner a main partner? A main partner is someone that you feel committed to above all others -- this is someone you might call your boyfriend, significant other, or life partner." is one of the following answers ("Yes") OR #149 Question "Next, think of whether you are currently in an active, ongoing sexual relationship with your male partner. By that, we mean that you generally have oral or anal sex at least once per month and you expect to continue doing so for some time. Is the relationship with this partner active and ongoing?" is one of the following answers ("Yes")) OR #150 Question "Was this a one-time partner?" is one of the following answers ("No"))**

Shortname / Alias: p6m_one_cism_freq

#### ****In the last 6 months, how often did you have anal sex with this partner? Please give us your best estimate.****

( ) No anal sex

( ) Less than once per month

( ) Once per month

( ) 2-3 times per month

( ) Once per week

( ) More than once per week

**Logic: Show/hide trigger exists. Hidden unless: #151 Question "In the last 6 months, how often did you have anal sex with this partner? Please give us your best estimate." is one of the following answers ("Less than once per month","Once per month","2-3 times per month","Once per week","More than once per week")**

Shortname / Alias: one_cism_sextype_

#### ****In the last 6 months, which of the following did you do with this partner?**** Check all that apply.

[ ] Receptive anal sex (you bottomed)

[ ] Insertive anal sex (you topped)

**Logic: Hidden unless: #152 Question "In the last 6 months, which of the following did you do with this partner? Check all that apply." is one of the following answers ("Receptive anal sex (you bottomed)")**

Shortname / Alias: one_cism_recept_cond

#### ****In the past 6 months, when you had receptive anal sex (you bottomed) with this partner, how often were you fully protected by a condom? This means you or your partner used a condom the entire time you had sex, and the condom did not break or fall off.****

( ) Never

( ) Rarely

( ) Sometimes

( ) Often

( ) Always

( ) Prefer not to answer

( ) Don't know

**Logic: Hidden unless: #152 Question "In the last 6 months, which of the following did you do with this partner? Check all that apply." is one of the following answers ("Insertive anal sex (you topped)")**

Shortname / Alias: one_cism_insert_cond

#### ****In the past 6 months, when you had insertive anal sex (you topped) with this partner, how often were you fully protected by a condom? This means you or your partner used a condom the entire time you had sex, and the condom did not break or fall off.****

( ) Never

( ) Rarely

( ) Sometimes

( ) Often

( ) Always

( ) Prefer not to answer

( ) Don't know

**Page entry logic:** This page will show when: #124 Question "**During the last 6 months, with how many cisgender male partner(s) did you have anal or oral sex?**" is greater than "1"

## >1 Cis Male Partner

**Logic: Hidden by default**

### ****The number of active and ongoing partners is greater than the total number of cisgender male partners.****

Validation: Min = 0 Max = 999 Must be numeric Whole numbers only Positive numbers only Max character count = 3 Min character count = 1

Shortname / Alias: mto_cism_active

### ****Earlier you told us you have had [question('value'), id='116'] cisgender male partners in the past 6 months (since [question('value'), id='3']). How many of your partners are active and ongoing? By that, we mean that you generally have oral or anal sex at least once per month and you expect to continue doing so for some time.****

_________________________________________________

**Logic: Show/hide trigger exists.**

Shortname / Alias: mto_cism_mainpart

#### ****Are/were any of these [question('value'), id='116'] partners a main partner? A main partner is someone that you feel committed to above all others - this is someone you might call your boyfriend, significant other, or spouse.****

( ) Yes

( ) No

( ) Prefer not to answer

( ) Don't know

**Logic: Hidden unless: #156 Question "Are/were any of these [question('value'), id='116'] partners a main partner? A main partner is someone that you feel committed to above all others - this is someone you might call your boyfriend, significant other, or spouse." is one of the following answers ("Yes")**

Shortname / Alias: mto_cism_caspart

#### ****Are/were any of these [question('value'), id='116'] partners a non-main (casual) partner?****

( ) Yes

( ) No

( ) Prefer not to answer

( ) Don't know

**Page entry logic:** This page will show when: #124 Question "**During the last 6 months, with how many cisgender male partner(s) did you have anal or oral sex?**" is greater than "1"

## Partner answer validation

**Page entry logic:** This page will show when: #156 Question "**Are/were any of these [question('value'), id='116'] partners a main partner? A main partner is someone that you feel committed to above all others - this is someone you might call your boyfriend, significant other, or spouse.**" is one of the following answers ("Yes")

## >1 Cis Male At Least 1 Main

### ****For the next few questions we are interested in hearing about your main partner(s) over the past 6 months (since [question('value'), id='3']).****

**Logic: Show/hide trigger exists.**

Shortname / Alias: p6m_main_anal

#### ****In the last 6 months, how often did you have anal sex with your main partner(s)?****

( ) No anal sex

( ) Less than once per month

( ) Once per month

( ) 2-3 times per month

( ) Once per week

( ) More than once per week

**Logic: Show/hide trigger exists. Hidden unless: #158 Question "In the last 6 months, how often did you have anal sex with your main partner(s)?" is one of the following answers ("Less than once per month","Once per month","2-3 times per month","Once per week","More than once per week")**

Shortname / Alias: main_cism_sextype_

#### ****In the last 6 months, which of the following did you do with your main partner(s)?**** Check all that apply.

[ ] Receptive anal sex (you bottomed with him)

[ ] Insertive anal sex (you topped with him)

**Logic: Hidden unless: #159 Question "In the last 6 months, which of the following did you do with your main partner(s)? Check all that apply." is one of the following answers ("Receptive anal sex (you bottomed with him)")**

Shortname / Alias: main_cism_recept_cond

#### ****In the past 6 months, when you had receptive anal sex (you bottomed) with your main partner(s), how often were you fully protected by a condom? This means you or your partner(s) used a condom the entire time you had sex, and the condom did not break or fall off.****

( ) Never

( ) Rarely

( ) Sometimes

( ) Often

( ) Always

( ) Prefer not to answer

( ) Don't know

**Logic: Hidden unless: #159 Question "In the last 6 months, which of the following did you do with your main partner(s)? Check all that apply." is one of the following answers ("Insertive anal sex (you topped with him)")**

Shortname / Alias: main_cism_insert_cond

#### ****In the past 6 months, when you had insertive anal sex (you topped) with your main partner(s), how often were you fully protected by a condom? This means you or your partner(s) used a condom the entire time you had sex, and the condom did not break or fall off.****

( ) Never

( ) Rarely

( ) Sometimes

( ) Often

( ) Always

( ) Prefer not to answer

( ) Don't know

**Page entry logic:** This page will show when: ((#157 Question "**Are/were any of these [question('value'), id='116'] partners a non-main (casual) partner?**" is not one of the following answers ("No","Prefer not to answer","Don't know") OR #156 Question "**Are/were any of these [question('value'), id='116'] partners a main partner? A main partner is someone that you feel committed to above all others - this is someone you might call your boyfriend, significant other, or spouse.**" is not one of the following answers ("Yes")) AND #124 Question "**During the last 6 months, with how many cisgender male partner(s) did you have anal or oral sex?**" is greater than "1")

## >1 Cis Male At Least 1 Casual

### ****For the next few questions we are interested in hearing about your non-main partners over the past 6 months (since [question('value'), id='3']).****

**Logic: Show/hide trigger exists.**

Shortname / Alias: p6m_cas_anal

#### ****In the last 6 months, how often did you have anal sex with your non-main partner(s)?****

( ) No anal sex

( ) Less than once per month

( ) Once per month

( ) 2-3 times per month

( ) Once per week

( ) More than once per week

( ) I did not have any non-main partners

**Logic: Show/hide trigger exists. Hidden unless: #162 Question "In the last 6 months, how often did you have anal sex with your non-main partner(s)?" is one of the following answers ("Less than once per month","Once per month","2-3 times per month","Once per week","More than once per week")**

Shortname / Alias: cas_cism_sextype_

#### ****In the last 6 months, which of the following did you do with your non-main partner(s)?**** Check all that apply.

[ ] Receptive anal sex (you bottomed with him)

[ ] Insertive anal sex (you topped with him)

**Logic: Hidden unless: #163 Question "In the last 6 months, which of the following did you do with your non-main partner(s)? Check all that apply." is one of the following answers ("Receptive anal sex (you bottomed with him)")**

Shortname / Alias: cas_cism_recept_cond

#### ****In the past 6 months, when you had receptive anal sex (you bottomed) with your non-main partner(s), how often were you fully protected by a condom? This means you or your partner used a condom the entire time you had sex, and the condom did not break or fall off.****

( ) Never

( ) Rarely

( ) Sometimes

( ) Often

( ) Always

( ) Prefer not to answer

( ) Don't know

**Logic: Hidden unless: #163 Question "In the last 6 months, which of the following did you do with your non-main partner(s)? Check all that apply." is one of the following answers ("Insertive anal sex (you topped with him)")**

Shortname / Alias: cas_cism_insert_cond

#### ****In the past 6 months, when you had insertive anal sex (you topped) with your non-main partner(s), how often were you fully protected by a condom? This means you or your partner(s) used a condom the entire time you had sex, and the condom did not break or fall off.****

( ) Never

( ) Rarely

( ) Sometimes

( ) Often

( ) Always

( ) Prefer not to answer

( ) Don't know

**Page entry logic:** This page will show when: ((((#123 Question "**In the past 6 months (since [question('value'), id='3']), what types of sex have you had with cisgender men (assigned male sex at birth and identifies as male)?**" is one of the following answers ("Anal sex (penis in the butt)") OR #127 Question "**In the past 6 months (since [question('value'), id='3']), what types of sex have you had with cisgender women (assigned female sex at birth and identifies as female)?**" is one of the following answers ("Anal sex (penis in the butt)")) OR #132 Question "**In the past 6 months (since [question('value'), id='3']), what types of sex have you had with transgender female or transfeminine people (assigned male sex at birth and identifies as transfeminine or female)?**" is one of the following answers ("Anal sex (penis in the butt)")) OR #137 Question "**In the past 6 months (since [question('value'), id='3']), what types of sex have you had with transgender male or transmasculine people (assigned female sex at birth and identifies as transmasculine or male)?**" is one of the following answers ("Anal sex (penis in the butt)")) OR #142 Question "**In the past 6 months (since [question('value'), id='3']), what types of sex have you had with non-binary/gender non-conforming/genderqueer/two-spirit people?**" is one of the following answers ("Anal sex (penis in the butt)"))

## HIV-Positive Partners

**Logic: Show/hide trigger exists.**

Shortname / Alias: known_hiv_pos_analsex

#### ****In the past 6 months (since [question('value'), id='3']), have you had anal sex with any partners who you knew to be living with HIV?****

( ) Yes

( ) No

( ) Prefer not to answer

( ) Don't know

**Logic: Hidden unless: #166 Question "In the past 6 months (since [question('value'), id='3']), have you had anal sex with any partners who you knew to be living with HIV?" is one of the following answers ("Yes")**

Shortname / Alias: HIVpos_findout

#### ****When did you find out or when were you told that your sex partner was living with HIV?****

( ) Before having sex

( ) After having sex

**Logic: Hidden by default**

### ****The number of HIV-positive partners that you entered is greater than your total number of partners ([question('value'), id='420']) in the last 6 months.****

Validation: Min = 0 Max = 999 Must be numeric Whole numbers only Positive numbers only Max character count = 3 Min character count = 1

**Logic: Hidden unless: #166 Question "In the past 6 months (since [question('value'), id='3']), have you had anal sex with any partners who you knew to be living with HIV?" is one of the following answers ("Yes")**

Shortname / Alias: HIVpos_totpart

### ****In the past 6 months, how many of your partners were living with HIV?****

_________________________________________________

**Logic: Show/hide trigger exists. Hidden unless: #166 Question "In the past 6 months (since [question('value'), id='3']), have you had anal sex with any partners who you knew to be living with HIV?" is one of the following answers ("Yes")**

Shortname / Alias: hivpos_sextype_

#### ****In the last 6 months, which of the following did you do with partner(s) who were living with HIV?****Check all that apply.

[ ] Receptive anal sex (you bottomed)

[ ] Insertive anal sex (you topped)

**Logic: Hidden unless: #169 Question "In the last 6 months, which of the following did you do with partner(s) who were living with HIV? Check all that apply." is one of the following answers ("Receptive anal sex (you bottomed)")**

Shortname / Alias: hivpos_recept_cond

#### ****In the past 6 months, when you had receptive anal sex (you bottomed) with a partner who was living with HIV, how often were you fully protected by a condom? This means you or your partner used a condom the entire time you had sex, and the condom did not break or fall off.****

( ) Never

( ) Rarely

( ) Sometimes

( ) Often

( ) Always

( ) Prefer not to answer

( ) Don't know

**Logic: Hidden unless: #169 Question "In the last 6 months, which of the following did you do with partner(s) who were living with HIV? Check all that apply." is one of the following answers ("Insertive anal sex (you topped)")**

Shortname / Alias: hivpos_insert_cond

#### ****In the past 6 months, when you had insertive anal sex (you topped) with a partner who was living with HIV, how often were you fully protected by a condom? This means you or your partner used a condom the entire time you had sex, and the condom did not break or fall off.****

( ) Never

( ) Rarely

( ) Sometimes

( ) Often

( ) Always

( ) Prefer not to answer

( ) Don't know

**Logic: Show/hide trigger exists. Hidden unless: #166 Question "In the past 6 months (since [question('value'), id='3']), have you had anal sex with any partners who you knew to be living with HIV?" is one of the following answers ("Yes")**

Shortname / Alias: hivpart_untransmit

#### ****In the past 6 months, did any of your partner(s) living with HIV tell you that they were untransmittable or had an undetectable viral load (i.e., U=U)?****

( ) Yes

( ) No

( ) Prefer not to answer

( ) Don't know

**Logic: Hidden unless: #172 Question "In the past 6 months, did any of your partner(s) living with HIV tell you that they were untransmittable or had an undetectable viral load (i.e., U=U)?" is one of the following answers ("Yes")**

Shortname / Alias: all_hiv_part_untransmit

#### ****In the past 6 months, did all of your partner(s) living with HIV tell you that they were untransmittable or had an undetectable viral load (i.e., U=U)?****

( ) Yes

( ) No

( ) Prefer not to answer

( ) Don't know

**Page entry logic:** This page will show when: #166 Question "**In the past 6 months (since [question('value'), id='3']), have you had anal sex with any partners who you knew to be living with HIV?**" is one of the following answers ("Yes")

## HIV Partner Checking

**Page entry logic:** This page will show when: ((((#123 Question "**In the past 6 months (since [question('value'), id='3']), what types of sex have you had with cisgender men (assigned male sex at birth and identifies as male)?**" is not exactly equal to ("I have not had any type of sex with a cisgender man in the past 6 months") OR #127 Question "**In the past 6 months (since [question('value'), id='3']), what types of sex have you had with cisgender women (assigned female sex at birth and identifies as female)?**" is not exactly equal to ("I have not had any type of sex with a cisgender woman in the past 6 months")) OR #132 Question "**In the past 6 months (since [question('value'), id='3']), what types of sex have you had with transgender female or transfeminine people (assigned male sex at birth and identifies as transfeminine or female)?**" is not exactly equal to ("I have not had any type of sex with a transgender woman in the past 6 months")) OR #137 Question "**In the past 6 months (since [question('value'), id='3']), what types of sex have you had with transgender male or transmasculine people (assigned female sex at birth and identifies as transmasculine or male)?**" is not exactly equal to ("I have not had any type of sex with a transgender man in the past 6 months")) OR #142 Question "**In the past 6 months (since [question('value'), id='3']), what types of sex have you had with non-binary/gender non-conforming/genderqueer/two-spirit people?**" is not exactly equal to ("I have not had any type of sex with a non-binary/gender non-conforming/genderqueer/two-spirit person in the past 6 months"))

## Sexual Behavior: Unknown HIV Serostatus

**Logic: Show/hide trigger exists.**

Shortname / Alias: unknown_hiv_analsex

#### ****In the past 6 months (since [question('value'), id='3']), did you have anal sex WITHOUT a condom with a partner whose HIV status you did not know?****

( ) Yes

( ) No

( ) Prefer not to answer

( ) Don't know

**Logic: Show/hide trigger exists. Hidden unless: #174 Question "In the past 6 months (since [question('value'), id='3']), did you have anal sex WITHOUT a condom with a partner whose HIV status you did not know?" is one of the following answers ("Yes")**

Shortname / Alias: hivunk_sextype_

#### ****In the last 6 months, which of the following did you do with partner(s) whose HIV status you did not know?**** Check all that apply.

[ ] Receptive anal sex (you bottomed)

[ ] Insertive anal sex (you topped)

**Logic: Hidden unless: #175 Question "In the last 6 months, which of the following did you do with partner(s) whose HIV status you did not know? Check all that apply." is one of the following answers ("Receptive anal sex (you bottomed)")**

Shortname / Alias: hivunk_recept_cond

#### ****In the past 6 months, when you had receptive anal sex (you bottomed) with a partner whose HIV status you did not know, how often were you fully protected by a condom? This means you or your partner used a condom the entire time you had sex, and the condom did not break or fall off.****

( ) Never

( ) Rarely

( ) Sometimes

( ) Often

( ) Always

( ) Prefer not to answer

( ) Don't know

**Logic: Hidden unless: #175 Question "In the last 6 months, which of the following did you do with partner(s) whose HIV status you did not know? Check all that apply." is one of the following answers ("Insertive anal sex (you topped)")**

Shortname / Alias: hivunk_insert_cond

#### ****In the past 6 months, when you had insertive anal sex (you topped) with a partner whose HIV status you did not know, how often were you fully protected by a condom? This means you or your partner used a condom the entire time you had sex, and the condom did not break or fall off.****

( ) Never

( ) Rarely

( ) Sometimes

( ) Often

( ) Always

( ) Prefer not to answer

( ) Don't know

Shortname / Alias: alc_intercourse

#### ****In the past 6 months, how often did you drink alcohol or use drugs before you had sexual intercourse?****

( ) Never

( ) Rarely

( ) Sometimes

( ) Often

( ) Always

## GIF8

###

## DAST-10

### ****In the questions that follow we are going to ask about your drug use. This refers to any nonmedical drugs that you take or any prescriptions that you take in excess of the directions.****

**Logic: Show/hide trigger exists.**

Shortname / Alias: druguse_nonmedical

#### ****Have you used drugs other than those required for medical reasons in the past 6 months (since [question('value'), id='3'])?****

( ) Yes

( ) No

( ) Prefer not to answer

( ) Don't know

**Logic: Hidden unless: #179 Question "Have you used drugs other than those required for medical reasons in the past 6 months (since [question('value'), id='3'])?" is one of the following answers ("Yes")**

Shortname / Alias: druguse_mto

#### ****Do you use more than one drug at a time?****

( ) Yes

( ) No

( ) Prefer not to answer

( ) Don't know

**Logic: Hidden unless: #179 Question "Have you used drugs other than those required for medical reasons in the past 6 months (since [question('value'), id='3'])?" is one of the following answers ("Yes")**

Shortname / Alias: druguse_stop

#### ****Are you always able to stop using drugs when you want to?****

( ) Yes

( ) No

( ) Prefer not to answer

( ) Don't know

**Logic: Hidden unless: #179 Question "Have you used drugs other than those required for medical reasons in the past 6 months (since [question('value'), id='3'])?" is one of the following answers ("Yes")**

Shortname / Alias: druguse_blackout

#### ****Have you had "blackouts" or "flashbacks" as a result of drug use?****

( ) Yes

( ) No

( ) Prefer not to answer

( ) Don't know

**Logic: Hidden unless: #179 Question "Have you used drugs other than those required for medical reasons in the past 6 months (since [question('value'), id='3'])?" is one of the following answers ("Yes")**

Shortname / Alias: druguse_guilt

#### ****Do you ever feel bad or guilty about your drug use?****

( ) Yes

( ) No

( ) Prefer not to answer

( ) Don't know

**Logic: Hidden unless: #179 Question "Have you used drugs other than those required for medical reasons in the past 6 months (since [question('value'), id='3'])?" is one of the following answers ("Yes")**

Shortname / Alias: druguse_complain

#### ****Does your spouse (or parents) ever complain about your involvement with drugs?****

( ) Yes

( ) No

( ) Prefer not to answer

( ) Don't know

**Logic: Hidden unless: #179 Question "Have you used drugs other than those required for medical reasons in the past 6 months (since [question('value'), id='3'])?" is one of the following answers ("Yes")**

Shortname / Alias: druguse_neglect

#### ****Have you neglected your family because of your use of drugs?****

( ) Yes

( ) No

( ) Prefer not to answer

( ) Don't know

**Logic: Hidden unless: #179 Question "Have you used drugs other than those required for medical reasons in the past 6 months (since [question('value'), id='3'])?" is one of the following answers ("Yes")**

Shortname / Alias: druguse_illegal

#### ****Have you engaged in illegal activities in order to obtain drugs?****

( ) Yes

( ) No

( ) Prefer not to answer

( ) Don't know

**Logic: Hidden unless: #179 Question "Have you used drugs other than those required for medical reasons in the past 6 months (since [question('value'), id='3'])?" is one of the following answers ("Yes")**

Shortname / Alias: druguse_withdrawal

#### ****Have you ever experienced withdrawal symptoms (felt sick) when you stopped taking drugs?****

( ) Yes

( ) No

( ) Prefer not to answer

( ) Don't know

**Logic: Hidden unless: #179 Question "Have you used drugs other than those required for medical reasons in the past 6 months (since [question('value'), id='3'])?" is one of the following answers ("Yes")**

Shortname / Alias: druguse_medicalprob

#### ****Have you had medical problems as a result of your drug use (e.g., memory loss, hepatitis, convulsions, bleeding, etc.)?****

( ) Yes

( ) No

( ) Prefer not to answer

( ) Don't know

**Page entry logic:** This page will show when: #179 Question "**Have you used drugs other than those required for medical reasons in the past 6 months (since [question('value'), id='3'])?**" is one of the following answers ("Yes")

## Substance Use

### ****Now we’re going to ask some questions about your substance use history. Please remember that your answers will be kept private. You may skip any questions you are not comfortable answering. Please let us know which questions are not applicable to you. We need to ask you all of these questions, even if some may not apply to your situation.****

**Logic: Show/hide trigger exists.**

Shortname / Alias: drug_

#### ****In the past 6 months (since [question('value'), id='3']), which drugs that were not prescribed to you did you use? (Select all that you have taken in the past 6 months)****

[ ] Cannabis (marijuana, pot, grass, hash, etc.)

[ ] Cocaine (coke, crack, etc.)

[ ] Prescription stimulants just for the feeling, more than prescribed, or that were not prescribed for you (Ritalin, Adderall, diet pills, etc.)

[ ] Methamphetamine (meth, crystal, speed, ecstasy, molly, etc.)

[ ] Inhalants (nitrous, glue, paint thinner, poppers, whippets, etc.)

[ ] Sedatives just for the feeling, more than prescribed, or that were not prescribed for you (sleeping pills, Valium, Xanax, tranquilizers, benzos, etc.)

[ ] Hallucinogens (LSD, acid, mushrooms, PCP, Special K, ecstasy, etc.)

[ ] Street opioids (heroin, opium, etc.)

[ ] Prescription opioids just for the feeling, more than prescribed, or that were not prescribed for you (Fentanyl, Oxycodone, OxyContin, Percocet, Vicodin, methadone, Buprenorphine, etc.)

[ ] Any other drugs to get high; please specify:: _________________________________________________*

[ ] Prefer not to answer

[ ] Don't know

[ ] None of the above

## Substance Follow-Up Questions

**Logic: Hidden unless: #189 Question "In the past 6 months (since [question('value'), id='3']), which drugs that were not prescribed to you did you use? (Select all that you have taken in the past 6 months)" is one of the following answers ("Cannabis (marijuana, pot, grass, hash, etc.)","Cocaine (coke, crack, etc.)","Prescription stimulants just for the feeling, more than prescribed, or that were not prescribed for you (Ritalin, Adderall, diet pills, etc.)","Methamphetamine (meth, crystal, speed, ecstasy, molly, etc.)","Inhalants (nitrous, glue, paint thinner, poppers, whippets, etc.)","Sedatives just for the feeling, more than prescribed, or that were not prescribed for you (sleeping pills, Valium, Xanax, tranquilizers, benzos, etc.)","Hallucinogens (LSD, acid, mushrooms, PCP, Special K, ecstasy, etc.)","Street opioids (heroin, opium, etc.)","Prescription opioids just for the feeling, more than prescribed, or that were not prescribed for you (Fentanyl, Oxycodone, OxyContin, Percocet, Vicodin, methadone, Buprenorphine, etc.)","Any other drugs to get high; please specify:")**

Shortname / Alias: subst_freq

Piping: Piped From Question 189. (**In the past 6 months (since [question('value'), id='3']), which drugs that were not prescribed to you did you use? (Select all that you have taken in the past 6 months)**)

#### ****In the past 6 months (since [question('value'), id='3']), how often did you use [question('piped title')]?****

( ) Less than once a month

( ) Once a month

( ) More than once a month

( ) Once a week

( ) More than once a week

( ) Once a day

( ) More than once a day

( ) Prefer not to answer

( ) Don't know

**Page entry logic:** This page will show when: #179 Question "**Have you used drugs other than those required for medical reasons in the past 6 months (since [question('value'), id='3'])?**" is one of the following answers ("Yes")

## Injection Drug Use

**Logic: Show/hide trigger exists.**

Shortname / Alias: p6m_inject

#### ****In the past 6 months (since [question('value'), id='3']), have you shot up or injected any drugs other than those prescribed for you?**** By shooting up, we mean anytime you might have used a needle to inject drugs in your veins, under the skin, or in the muscle.

( ) Yes

( ) No

( ) Prefer not to answer

( ) Don't know

**Logic: Show/hide trigger exists. Hidden unless: #190 Question "In the past 6 months (since [question('value'), id='3']), have you shot up or injected any drugs other than those prescribed for you? By shooting up, we mean anytime you might have used a needle to inject drugs in your veins, under the skin, or in the muscle." is one of the following answers ("Yes")**

Shortname / Alias: p6m_inject_freq

#### ****In the past 6 months, about how often did you inject?****

( ) More than once a day

( ) Once a day

( ) More than once a week

( ) Once a week or less

( ) Prefer not to answer

( ) Don't know

**Logic: Show/hide trigger exists. Hidden unless: #191 Question "In the past 6 months, about how often did you inject?" is one of the following answers ("More than once a day","Once a day","More than once a week","Once a week or less")**

Shortname / Alias: p6m_needleshare

#### ****In the past 6 months, have you injected by using needles, syringes, or other drug preparation equipment (works) that had already been used by another person?****

( ) Yes

( ) No

( ) Prefer not to answer

( ) Don't know

**Logic: Hidden unless: #192 Question "In the past 6 months, have you injected by using needles, syringes, or other drug preparation equipment (works) that had already been used by another person?" is one of the following answers ("Yes")**

Shortname / Alias: shareneedle_hivpos

#### ****Did any of the people that shared their needles with you have a positive HIV status or an HIV status that was unknown to you?****

( ) Yes

( ) No

( ) Prefer not to answer

( ) Don't know

## AUDIT-C

### ****A standard drink is typically 12 ounces of regular beer, 8-9 ounces of malt liquor, 5 ounces of wine, or 1.5 ounces of distilled spirits (hard alcohol). See the picture below for reference.****

**Logic: Show/hide trigger exists.**

Shortname / Alias: alc_drink_freq

#### ****How often do you have a drink containing alcohol?****

( ) Never

( ) Monthly

( ) 2-4 times a month

( ) 2-3 times a week

( ) 4 or more times a week

( ) Prefer not to answer

**Logic: Hidden unless: #194 Question "How often do you have a drink containing alcohol?" is one of the following answers ("Monthly","2-4 times a month","2-3 times a week","4 or more times a week")**

Shortname / Alias: alc_drink_perday

#### ****When you consume alcohol, how many standard drinks do you have in one sitting?****

( ) 1 to 2

( ) 3 to 4

( ) 5 to 6

( ) 7 to 9

( ) 10 or more

**Logic: Hidden unless: #194 Question "How often do you have a drink containing alcohol?" is one of the following answers ("Monthly","2-4 times a month","2-3 times a week","4 or more times a week")**

Shortname / Alias: alc_6drink_freq

#### ****How often do you have six or more drinks on one occasion?****

( ) Daily or almost daily

( ) Weekly

( ) Monthly

( ) Less than monthly

( ) Never

## GIF5

###

## Mental Health: PHQ-9

### ****Now we're going to ask you some questions about your mood. When answering, please think about how often the following has occurred during the past 2 weeks.****

Shortname / Alias: phq9_

#### ****Over the past 2 weeks, how often have you been bothered by the following problems?****

|  | **Not at all** | **Several days** | **More than half the days** | **Nearly every day** | **Prefer not to answer** | **Don't know** |
| --- | --- | --- | --- | --- | --- | --- |
| **Little interest or pleasure in doing things** | ( ) | ( ) | ( ) | ( ) | ( ) | ( ) |
| **Feeling down, depressed, or hopeless** | ( ) | ( ) | ( ) | ( ) | ( ) | ( ) |
| **Trouble falling or staying asleep, or sleeping too much** | ( ) | ( ) | ( ) | ( ) | ( ) | ( ) |
| **Feeling tired or having little energy** | ( ) | ( ) | ( ) | ( ) | ( ) | ( ) |
| **Poor appetite or overeating** | ( ) | ( ) | ( ) | ( ) | ( ) | ( ) |
| **Feeling bad about yourself – or that you are a failure or have let yourself or your family down** | ( ) | ( ) | ( ) | ( ) | ( ) | ( ) |
| **Trouble concentrating on things, such as reading the newspaper or watching television** | ( ) | ( ) | ( ) | ( ) | ( ) | ( ) |
| **Moving or speaking so slowly that other people could have noticed. Or the opposite – being so fidgety or restless that you have been moving around a lot more than usual** | ( ) | ( ) | ( ) | ( ) | ( ) | ( ) |
| **Thoughts that you would be better off dead, or of hurting yourself** | ( ) | ( ) | ( ) | ( ) | ( ) | ( ) |

## Mental Health: GAD-7

Shortname / Alias: gad7_

#### ****Over the past 2 weeks, how often have you been bothered by the following problems?****

|  | **Not at all** | **Several days** | **More than half the days** | **Nearly every day** | **Prefer not to answer** | **Don't know** |
| --- | --- | --- | --- | --- | --- | --- |
| **Feeling nervous, anxious, or on edge** | ( ) | ( ) | ( ) | ( ) | ( ) | ( ) |
| **Not being able to stop or control worrying** | ( ) | ( ) | ( ) | ( ) | ( ) | ( ) |
| **Worrying too much about different things** | ( ) | ( ) | ( ) | ( ) | ( ) | ( ) |
| **Trouble relaxing** | ( ) | ( ) | ( ) | ( ) | ( ) | ( ) |
| **Being so restless that it is hard to sit still** | ( ) | ( ) | ( ) | ( ) | ( ) | ( ) |
| **Becoming easily annoyed or irritable** | ( ) | ( ) | ( ) | ( ) | ( ) | ( ) |
| **Feeling afraid, as if something awful might happen** | ( ) | ( ) | ( ) | ( ) | ( ) | ( ) |

## Mental Health: Resiliency & Self-Efficacy

Shortname / Alias: resiliency_

#### ****Please read the following questions and check the boxes that indicate how you feel about yourself.****

|  | **Not at all true** | **Hardly true** | **Moderately true** | **Exactly true** | **Prefer not to answer** | **Don't know** |
| --- | --- | --- | --- | --- | --- | --- |
| **I can always manage to solve difficult problems if I try hard enough.** | ( ) | ( ) | ( ) | ( ) | ( ) | ( ) |
| **If someone opposes me, I can find the means and ways to get what I want.** | ( ) | ( ) | ( ) | ( ) | ( ) | ( ) |
| **It is easy for me to stick to my aims and accomplish my goals.** | ( ) | ( ) | ( ) | ( ) | ( ) | ( ) |
| **I am confident that I could deal efficiently with unexpected events.** | ( ) | ( ) | ( ) | ( ) | ( ) | ( ) |
| **Thanks to my resourcefulness, I know how to handle unforeseen situations.** | ( ) | ( ) | ( ) | ( ) | ( ) | ( ) |
| **I can solve most problems if I invest the necessary effort.** | ( ) | ( ) | ( ) | ( ) | ( ) | ( ) |
| **I can remain calm when facing difficulties because I can rely on my coping abilities.** | ( ) | ( ) | ( ) | ( ) | ( ) | ( ) |
| **When I am confronted with a problem, I can usually find several solutions.** | ( ) | ( ) | ( ) | ( ) | ( ) | ( ) |
| **If I am in trouble, I can usually think of a solution.** | ( ) | ( ) | ( ) | ( ) | ( ) | ( ) |

## Mental Health: Emotional & Instrumental Support

Shortname / Alias: support1_

#### ****People sometimes look to others for companionship, assistance, or other types of support. Check the boxes to indicate how often each of the following kinds of support is available to you if you need it.****

|  | **Never** | **Rarely** | **Sometimes** | **Usually** | **Always** | **Prefer not to answer** | **Don't know** |
| --- | --- | --- | --- | --- | --- | --- | --- |
| **I have someone who will listen to me when I need to talk.** | ( ) | ( ) | ( ) | ( ) | ( ) | ( ) | ( ) |
| **I have someone to confide in or talk to about myself or my problems.** | ( ) | ( ) | ( ) | ( ) | ( ) | ( ) | ( ) |
| **I have someone who makes me feel appreciated.** | ( ) | ( ) | ( ) | ( ) | ( ) | ( ) | ( ) |
| **I have someone to talk with when I have a bad day.** | ( ) | ( ) | ( ) | ( ) | ( ) | ( ) | ( ) |

Shortname / Alias: support2_

####

|  | **Never** | **Rarely** | **Sometimes** | **Usually** | **Always** | **Prefer not to answer** | **Don't know** |
| --- | --- | --- | --- | --- | --- | --- | --- |
| **I have someone to give me good advice about a crisis if I need it.** | ( ) | ( ) | ( ) | ( ) | ( ) | ( ) | ( ) |
| **I have someone to turn to for suggestions about how to deal with a problem.** | ( ) | ( ) | ( ) | ( ) | ( ) | ( ) | ( ) |
| **I have someone to give me information if I need it.** | ( ) | ( ) | ( ) | ( ) | ( ) | ( ) | ( ) |
| **I get useful advice about important things in life.** | ( ) | ( ) | ( ) | ( ) | ( ) | ( ) | ( ) |

Shortname / Alias: support3_

####

|  | **Never** | **Rarely** | **Sometimes** | **Usually** | **Always** | **Prefer not to answer** | **Don't know** |
| --- | --- | --- | --- | --- | --- | --- | --- |
| **Do you have someone to help you if you are confined to bed?** | ( ) | ( ) | ( ) | ( ) | ( ) | ( ) | ( ) |
| **Do you have someone to take you to the doctor if you need it?** | ( ) | ( ) | ( ) | ( ) | ( ) | ( ) | ( ) |
| **Do you have someone to help with your daily chores if you are sick?** | ( ) | ( ) | ( ) | ( ) | ( ) | ( ) | ( ) |
| **Do you have someone to run errands if you need it?** | ( ) | ( ) | ( ) | ( ) | ( ) | ( ) | ( ) |

## Mental Health: Companionship & Social Isolation

Shortname / Alias: companion_

#### ****People sometimes look to others for companionship, assistance, or other types of support. Check the boxes to indicate how often each of the following kinds of support is available to you if you need it.****

|  | **Never** | **Rarely** | **Sometimes** | **Usually** | **Always** | **Prefer not to answer** | **Don't know** |
| --- | --- | --- | --- | --- | --- | --- | --- |
| **Do you have someone with whom to have fun?** | ( ) | ( ) | ( ) | ( ) | ( ) | ( ) | ( ) |
| **Do you have someone with whom to relax?** | ( ) | ( ) | ( ) | ( ) | ( ) | ( ) | ( ) |
| **Do you have someone with whom you can do something enjoyable?** | ( ) | ( ) | ( ) | ( ) | ( ) | ( ) | ( ) |
| **Do you find companionship when you want it?** | ( ) | ( ) | ( ) | ( ) | ( ) | ( ) | ( ) |

Shortname / Alias: iso_

#### ****Please check the following boxes to indicate how often the following statements apply to you.****

|  | **Never** | **Rarely** | **Sometimes** | **Usually** | **Always** | **Prefer not to answer** | **Don't know** |
| --- | --- | --- | --- | --- | --- | --- | --- |
| **I feel left out.** | ( ) | ( ) | ( ) | ( ) | ( ) | ( ) | ( ) |
| **I feel that people barely know me.** | ( ) | ( ) | ( ) | ( ) | ( ) | ( ) | ( ) |
| **I feel isolated from others.** | ( ) | ( ) | ( ) | ( ) | ( ) | ( ) | ( ) |
| **I feel that people are around me but not with me.** | ( ) | ( ) | ( ) | ( ) | ( ) | ( ) | ( ) |

## Incorrectly Answered Questions

### ****You are almost finished!**** We understand that having a survey without a “back button” can be difficult because it does not allow you to make corrections to previous responses.

Shortname / Alias: correction_essay

### If you would like to change anything about your responses, or questions we should have asked, please describe the question(s) and what the response(s) should be, using the space below.

____________________________________________

____________________________________________

____________________________________________

____________________________________________

## Closing Language and Gift Card Preference

### ****You're almost done! Just one final question.****

Shortname / Alias: gift_card_pref

#### ****Once we validate your survey, we will send your $50 electronic gift card to your email address. Please let us know which gift card you would prefer.*****

( ) Amazon

( ) Target

( ) Walmart

## Thank You!

### Thank you for completing the survey! We really appreciate your participation in the Combine Study. Please allow us a couple of days to send your electronic gift card. If you have any questions in the meantime, you can contact us via email at [combine_rct@emory.edu](mailto:combine_rct@emory.edu).
